# Definition of Fiducial Points in the Normal Seismocardiogram

**DOI:** 10.1038/s41598-018-33675-6

**Published:** 2018-10-18

**Authors:** Kasper Sørensen, Samuel E. Schmidt, Ask S. Jensen, Peter Søgaard, Johannes J. Struijk

**Affiliations:** 1 Aalborg University, Department of Health Science and Technology, Aalborg, 9220 Denmark; 2 Aalborg University Hospital, Department of Cardiology, Aalborg, 9000 Denmark

## Abstract

The purpose of this work is to define fiducial points in the seismocardiogram (SCG) and to correlate them with physiological events identified in ultrasound images. For 45 healthy subjects the SCG and the electrocardiogram (ECG) were recorded simultaneously at rest. Immediately following the SCG and ECG recordings ultrasound images of the heart were also obtained at rest. For all subjects a mean SCG signal was calculated and all fiducial points (peaks and valleys) were identified and labeled in the same way across all signals. Eight physiologic events, including the valve openings and closings, were annotated from ultrasound as well and the fiducial points were correlated with those physiologic events. A total of 42 SCG signals were used in the data analysis. The smallest mean differences (±SD) between the eight events found in the ultrasound images and the fiducial points, together with their correlation coefficients (r) were: atrial systolic onset: −2 (±16) ms, r = 0.75 (p < 0.001); peak atrial inflow: 13 (±19) ms, r = 0.63 (p < 0.001); mitral valve closure: 4 (±11) ms, r = 0.71 (p < 0.01); aortic valve opening: −3 (±11) ms, r = 0.60 (p < 0.001); peak systolic inflow: 13 (±23) ms, r = 0.42 (p < 0.01); aortic valve closure: −5 (±12) ms, r = 0.94 (p < 0.001); mitral valve opening: −7 (±19) ms, r = 0.87 (p < 0.001) and peak early ventricular filling: −18 (±28 ms), r = 0.79 (p < 0.001). In conclusion eight physiologic events characterizeing the cardiac cycle, are associated with reproducible, well-defined fiducial points in the SCG.

## Introduction

Seismocardiography (SCG) is a technique of measuring the vibrations produced by the beating heart, where those vibrations are recorded from the chest using an accelerometer. The technique was introduced by Mounsey in 1957^1^ using an accelerometer developed for ballistocardiography (BCG) by Elliott *et al*.^2^. The first use of the word “seismocardiography” was in 1961 by Bozhenko^3^ and later by Baevski in 1964^4^ who investigated the use of SCG in relation to space flights, as an alternative to BCG, the latter being a measure of the recoil forces of the body as response to the intermittent blood flow^5^ and which, at the time, could not be used in space^6^.

After a period of relative silence, in 1990 Zanetti and Salerno introduced the technology in the USA^7,8^, but even though SCG promised to provide measures of cardiac contractivity and timing of events in the cardiac cycle, the technique never made it to the clinic and was again largely abandoned throughout the 1990s^9^. This was in part due to the advances in echocardiography and other medical imaging techniques^9^ and to the fact that the accelerometers used were large and heavy (up to 1 kg^10^) and thus not easy to use.

The emergence of micro electro mechanical systems (MEMS) technology has made it possible to produce much smaller and lighter accelerometers, capable of recording with a high sensitivity, which transformed SCG from a measuring method mainly used in supine position, to a wearable technology^9,11^. Wearable systems have been studied in relation to changes in the SCG related to posture, walking and the assessment of heart failure. In 2016 De Rienzo *et al*. found that the pre-ejection period (PEP) and left ventricular ejection time (LVET), estimated from the SCG, were significantly different between supine and standing positions^12^ with recordings from the MagIC-SCG vest developed in 2010^11^.

SCG is thus being revised as an easy to use, non-invasive measuring technique of the cardiac performance and health status. The signal is deterministic and even though it is more detailed than, for instance, the electrocardiogram (ECG), the SCG fiducial points related to characteristic events in the cardiac cycle can be found among subjects. Especially the Aortic Valve Opening (AO) point has been investigated extensively and is consistent among most studies^7,8,13,14^, although there is some disagreement as well^15,16^. Accurate determination of events, including AO, could make SCG a diagnostic tool for cardiac disease, comparable with the ECG. In addition, SCG could become an important tool in e.g., arterial pulse transit time (PTT) analysis for the assessment of vascular status^17–20^.

However, the relation between SCG fiducial points and the physiological events in the cardiac cycle still lack sufficient evidence. Moreover, the literature is inconsistent on the location and even the definition of the fiducial points, which limits the use of the SCG. In some studies echocardiography was used to correlate some features in the SCG with cardiac events^7,21^ and, even though this imaging technique is limited in temporal resolution, echocardiography is indeed a helpful tool to determine the occurrence of cardiac events.

The aims of this study were to define all waves in the SCG that are common among normal subjects, to define the fiducial points related to those waves, and to relate fiducial points and intervals between fiducial points to events in the cardiac cycle as observed in echocardiograms of the same subjects.

## Methods

In this study we collected SCGs and ultrasound data from a total of 45 healthy subjects without known heart diseases and correlated the fiducial points in the SCG with physiological events from the ultrasound images. The following sections describe the approach in detail.

### Subjects

The subjects were recruited via posters and flyers placed at Aalborg University (Denmark), and in local shopping malls in the city of Aalborg.

Forty-five subjects were recruited. Inclusion criteria were: Healthy male and females at the age of 20–80 years. Exclusion criteria were:

- Diagnosed with any cardiovascular disease
- Receiving cardiovascular related medications such as medication for hypertension and hyperlipidaemia
- Inability to co-operate during the trial.

### Ethical Considerations

The study protocol was approved by the scientific ethical committee of Northern Jutland (N-20120069). Subjects signed a written inform consent before participating in the study. All methods were performed in accordance with relevant guidelines and regulations.

### Experimental Design

For each subject basic demographic data (height, weight, age and sex) was recorded before the trial started. The subject was placed in a supine position and two accelerometers were attached to the subject’s skin with double adhesive tape. The accelerometers were Silicon Designs 1521 placed in a 3D-printed plastic housing (19 mm wide 21 mm long and 11 high, weighing 5 grams).

Three-lead ECG recording was performed with four electrodes, placed on the left and right shoulder and on the left and right iliac crests. The accelerometers and ECG were connected to an iWorx 228 data acquisition system and the signals were sampled at 5000 Hz to a PC using LabScribe software. Ultrasound images were recorded with a Vivid E9 (GE Healthcare, Milwaukee). The ECG electrodes from the Vivid E9 were placed in the same locations as the electrodes used to obtain ECG via the iWorx 228. This ensured alignment of the data recorded via iWorx and the echocardiograph using the ECG signals recorded on the two devices. The Vivid E9 ultrasound system was tested for a potential delay between the ECG signal and the image. This delay was found to be less than one frame (with an acquisition rate of 70 frames per second).

The subject lay supine for three minutes while recording baseline measurements of ECG and SCG. One accelerometer was placed in the xiphoid process for recording of SCG signal while one was placed in the inter costal space 4 (IC4). As described later, the signal recorded in IC4 was used in the signal processing step as a proxy for heart sound. Afterwards the three ultrasound images were recorded: Apical 4 and 5-chamber view with Pulsed Wave Doppler (PWD) and M-mode Tissue Doppler Image (TDI) of the mitral valve leaflets. During the echocardiography the iWorx system was still recording. Following the echocardiographic procedure data was recorded for 5 more minutes before the trial ended.

### Signal Processing of SCG Signals

All recorded data from the iWorx system was exported and processed in MATLAB (2016b. The MathWorks, Inc.). The signals were segmented into beats using the method described by Jensen *et al*.^22^. This method uses ECG lead I or II to divide the signal into individual heart beats based on the ECG R-peak. A mean SCG beat was calculated as well as a mean ECG beat, based on these individual beats, using the approach described in the following.

For the annotation process a mean SCG beat for each subject was calculated, where the systolic part and the diastolic part were separately aligned and averaged to consider the variable duration of the systole. The SCG signals were first filtered forward-backward with a 1^st^ order low-pass Butterworth filter with a cutoff frequency of 90 Hz and then forward-backward filtered with a 3^rd^ order high-pass Butterworth filter at 0.05 Hz. Using the algorithm described in^22^ allowed for alignment of the beats used to calculate the mean beat with respect to either the R-peak of the ECG or the diastolic complex. Annotation of fiducial points in the systolic complex was thus done with the beats aligned to the R-peak of the ECG. For fiducial points in the diastolic complex the SCG signals were aligned in time to second heart sound (S2). The heart sounds were obtained by filtering the acceleration signal from IC4 with a Butterworth bandpass filter of 1^st^ order and a cutoff frequency of 50 and 500 Hz. S2 was located as the second largest peak in the autocorrelation of the median heart sound.

The lag between the individual beats and the peak of the envelope was calculated using the cross correlation and by aligning the beats with respect to the median heart sound. A new median beat was then computed as well as the envelope to find the peak of the S2 peak again and to realign the individual beats once more using the cross correlation. This ensured that all beats were aligned to a median heart sound, that was not influenced by the initial alignment to the R-peak^22^.

As part of the segmentation method noisy beats were rejected^22^. This ensured that the mean beat is composed of beats with similar characteristics.

**Figure 1:**
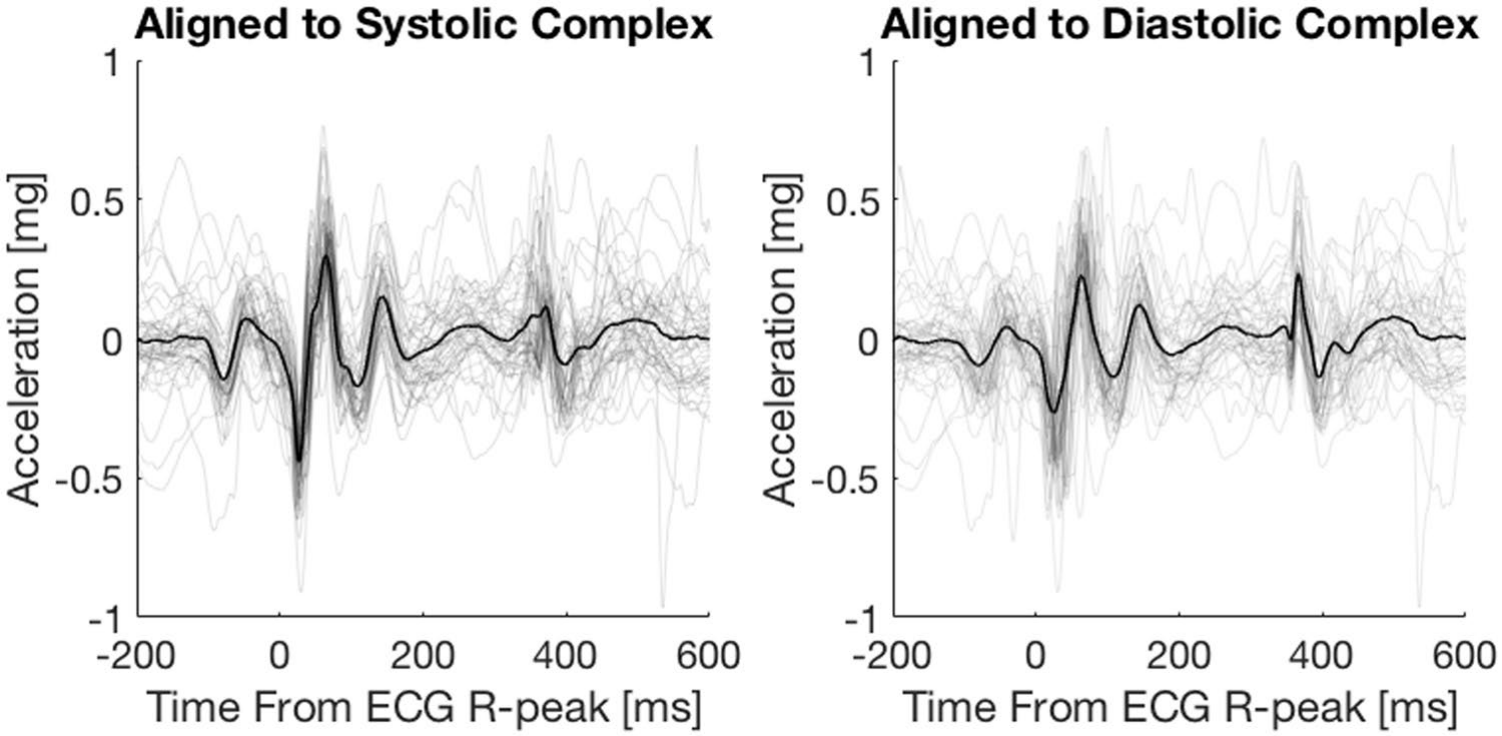
A seismocardiographic signal from one subject aligned to the ECG R-peak (systolic complex) and to the diastolic complex respectively. Individual beats are plotted in grey, the mean beats are plotted in black.

Because of the variability of the S1-S2 interval this procedure gave significantly different averages as compared with alignment based on the R-peak. The autocorrelation alignment thus ensured that no events were neglected to be marked: see Fig. 1, where the same signal is aligned to the systolic (ECG R-peak) complex and the diastolic complex respectively. The mean beat is visualized in black whereas the individual beats are visualized in grey.

### Annotation of Fiducial Points in the SCG signals

**Figure 2:**
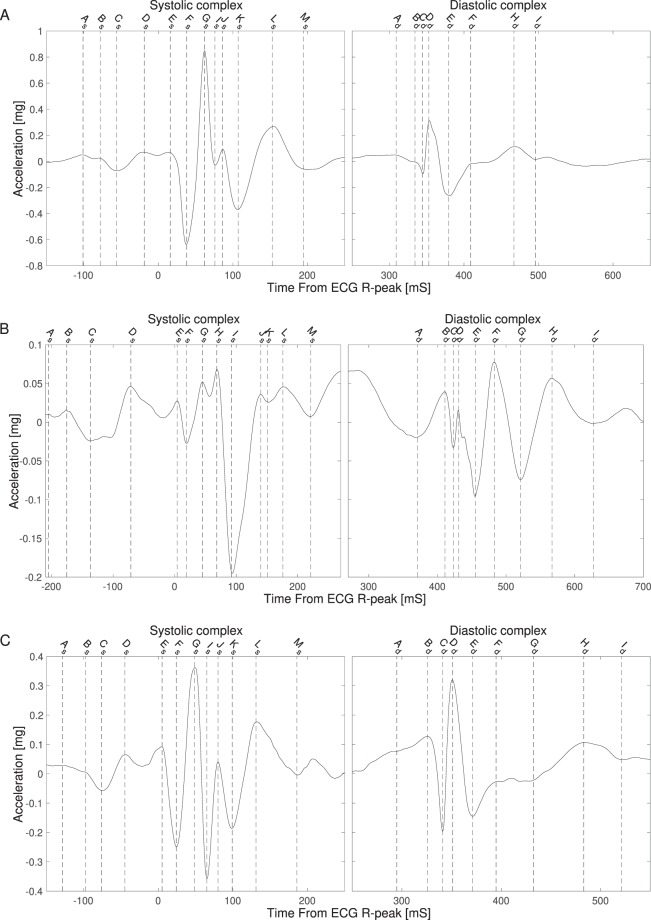
SCG signals from subject 09 (**A**), 15 (**B**) and 22 (**C**) with labeling of the fiducial points in the two complexes.

All SCG signals were manually annotated to ensure that fiducial points of the same characteristics were labeled the same way. During the labeling of the fiducial points the operator was blinded for the corresponding ultrasound images. A custom tool was developed in MATLAB for this purpose. The SCG signals were annotated in a process of two steps. First, all significant peaks and valleys in all mean beats were marked. In the second step the different events in the SCG signals were labeled using a simple naming scheme of the letters from A to M followed by subscript to indicate the systolic (s) or the diastolic (d) complex. Thus, As is fiducial point A in the systolic complex and Ad is fiducial point A in the diastolic complex. In this step fiducial points of similar characteristics were labeled with the same letter across the subjects. Thus, after this step the mean beats for some subjects could have fewer points labeled than for other subjects, depending on how many points could be identified in each subject. To a large extent the annotation process was iterative, and the operator went through the mean beats multiple times. Figure 2A–C shows three different mean beats each with a different number of events marked.

### Identification of common patterns in the SCG signal

#### Systolic Complex

The labeling of the fiducial points started with the first clear negative deflection of the main complex, following the ECG R-peak, the latter being defined as *t* = 0. This negative peak was labeled F_s_ (see Fig. 2). The small positive peak prior to the F-point was then labeled E_s_. It should be noted that at times this point was not presented as a distinct peak but as a shoulder. The positive peak prior to the E-peak was labeled D_s_, thus skipping the negative deflection between E_s_ and D_s_. The valley prior to D_s_ was labeled C_s_ and the two peaks prior to C_s_ were labeled B_s_ and A_s_. Thus, no valleys prior to C_s_ were labeled. Fiducial point A was not present in most of the signals, but it is shown in all signals in Fig. 2 as the first peak in the signal.

Moving to the right from fiducial point F_s_, the following highest peak in the systolic complex was identified. The valley following this peak was labeled I_s_. Between the two fiducial points F_s_ and I_s_ one, two or three peaks were present across all subjects. If one peak was present it was labeled G_s_. If two peaks were present, the first peak was labeled G_s_ and the following peak labeled H_s_, but only if there was more than 15 ms between the two peaks. If there was not a least 15 ms differences between the two points, the last peak was labeled G_s_ and no peak was labeled H_s_. In the case of three peaks only the two peaks closest to fiducial point F_s_ were considered. The same labeling convention as just described was used for those two peaks.

In three cases a shoulder near the peak of the systolic complex was labeled as fiducial point G_s_ (see SCG signals for subject N02, N11 and N31 in the online Supplementary Material).

The peak following the fiducial point I_s_ was labeled J_s_. The last valley marking the end of the systolic period was labeled M_s_. The peak and valley before M_s_ were labeled L_s_ and K_s_ respectively.

#### Diastolic Complex

In the diastolic complex, the first valley was labeled A_d_. This point was not always present as a distinct valley in which case the onset of the positive acceleration in the diastolic complex was labeled A_d_, see Fig. 2C.

The following peak was labeled B_d_. This fiducial point was in some cases present as the shoulder of a steep negative acceleration towards the valley labeled C_d_ (see Fig. 2C). The peak following C_d_ was labeled D_d_. Following D_d_ the deepest valley was labeled E_d_ and the following peaks and valleys were labeled F_d_, G_d_, H_d_ and finally I_d_.

### Ultrasound Image Processing

Processing of the images was done in the free DICOM Medical Images Viewer HOROS. The Pulsed Wave Doppler 4-chamber view of the mitral valve was used to measure the time difference between beginning and peak of both the E and A wave with respect to the R-peak of the ECG trace printed in the bottom of the ultrasound image.

The E wave corresponds to the early diastolic phase; thus, the beginning of the wave marks the onset of flow through the mitral valve. The start of the E wave will therefore be interpreted as the mitral valve opening. The following A wave is related to the late diastolic phase and is caused by the atrial contraction forcing blood into the ventricles^23,24^.

Using the Pulsed Wave Doppler 5-chamber view, the onset, top and end of the aortic blood flow profile was measured with respect to the R-peak. The onset of the flow profile from these images indicates when the aortic valve starts to open. Following the ejection profile there is a sharp closing click as the aortic valve closes^23,24^.

The closing-artifact describing mitral valve closure was not identifiable in most Pulsed Wave Doppler images. Thus, only tissue Doppler images were used to find the mitral valve closure point. The M-mode Tissue Doppler image of the Mitral Valve leaflets was used to find atrial systole (AS), MC, AO, aortic valve closure (AC) and mitral valve opening (MO), in accordance with^25^. All timings were related to the R-peak of the ECG trace on the image. All beats available in the images were analyzed and timings noted.

During the process of measuring the timings the operators were blinded for the corresponding SCG signals. Thus, operator bias was minimized.

### Validation of Time Points Location in the Ultrasound Images Among Operators

The Pulsed Wave Doppler images and the Tissue Doppler images were analyzed by two different operators respectively. To ensure that the operators agreed on where to place the markers for the different events, the following points were compared:

- Onset of the A-wave in the 4-chamber PWD Images and Atrial Systole found in the TDI Images
- Onset of the aortic flow profile found in the 5-chamber view PWD versus the AO from TDI
- End systolic flow profile from the 5-chamber view and the AC from the TDI
- Onset of the E-wave from the 4-chamber PWD Images and the Mitral Valve Opening from TDI.

Differences in timing >15 ms between the two images were reevaluated by the operators, although a reevaluation of a particular point did not always yield in a better agreement. For each of these four physiological events a mean time was calculated between the two image modalities to mitigate potential errors. Thus, each time point for e.g. Aortic Valve Opening consists of the mean of the times from both the TDI and PWD images.

### Statistical Analysis

All the time points of the fiducial points in the SCG and of physiological events in the ultrasound images were tested for normality by visualizing the histogram and probability plot of the data points. As normal distribution was observed among all data points Pearson’s Linear Correlation Coefficient was used to calculate the correlation between the fiducial points in the SCG and the timing of the physiological events found in the ultrasound images. All events from the systolic complex of the SCG signal were correlated with all physiological events during that complex and likewise for the diastolic complex. P-values < 0.05 was considered significant for rejecting the null hypothesis of no correlation between fiducial points and physiological events.

Besides the correlation, the mean differences between the fiducial points from the SCG and their corresponding time points from the ultrasound images were calculated together with their standard deviations. The time difference in combination with the correlation should give an indication as to which fiducial point from the SCG best corresponds to a certain physiological event found in the ultrasound images. For these points the beta coefficients for the linear regression is presented.

## Results

A total of 45 subjects participated in the study. One subject from the original 45 subjects decided to withdraw. Two subjects were excluded due to abnormal SCG signals with unidentifiable fiducial points. Thus, a total of 42 SCG signals were included in the study. For one subject the signal recording prior to the ultrasound scanning was interrupted by noise thus the signal recording following the ultrasound scanning was used instead. The mean age for the included subjects was 46.8 (±17.4) years with a mean weight of 72.9 (±12) kg and mean height of 174.9 (±8) cm and 52% of the subjects were female. The mean RR-interval was 950 (±177) ms.

Table 1 lists the number of the fiducial points labeled across the SCG signals. Tables 2 and 3 list how many physiological events were found in the pulsed wave Doppler images, and the Tissue Doppler images respectively. Ultrasound Images were included for all the 42 subjects.

**Table 1:** Fiducial points, the number of points marked and percentage of the number of subjects.

Fiducial Point	Number	Percentage of total
A_s_	11	26.2
B_s_	41	97.6
C_s_	42	100
D_s_	42	100
E_s_	42	100
F_s_	42	100
G_s_	42	100
H_s_	10	23.8
I_s_	41	97.6
J_s_	40	95.2
K_s_	42	100
L_s_	41	97.6
M_s_	41	97.6
A_d_	36	85.7
B_d_	42	100
C_d_	42	100
D_d_	42	100
E_d_	42	100
F_d_	36	85.7
G_d_	36	85.7
H_d_	41	97,6
I_d_	41	97,6

**Table 2:** Physiologic events found in the pulsed wave Doppler ultrasound images.

Event	Used images	Percentage of all images
Onset of Systolic Flow	42	100
Peak of Systolic Flow	42	100
Onset of A-wave	40	95.2
Peak of A-wave	40	95.2
End of Systolic Flow	42	100
Onset of E-wave	40	95.2
Peak of E-wave	40	95.2

**Table 3:** Physiologic events found in the Tissue Doppler ultrasound images.

Event	Used images	Percentage of all images
Atrial systolic	37	88.1
Mitral valve opening	41	97.6
Aortic valve opening	39	92.9
Aortic valve closure	39	92.9
Mitral valve opening	39	92.9

### Systolic complex

Table 4 shows the correlation between fiducial points in the systolic complex of the SCG and events found in the ultrasound images. Only results for which more than 15 points were used in the correlation calculation are included in Tables 4 and 5. Thus, two fiducial points were excluded: point A_s_ and point H_s_.

**Table 4:** Correlations, Difference and number of points between ultrasound images and seismocardiography Systolic complex.

Fiducial Point	Onset Atrial Systole			Peak A-Wave			Mitral Valve Closure			Aortic Valve Opening			Peak Systolic Inflow		
	R	Diff (±SD) [ms]	Points	R	Diff (±SD) [ms]	Points	R	Diff (±SD) [ms]	Points	R	Diff (±SD) [ms]	Points	R	Diff (±SD) [ms]	Points
B_s_	0.75**	−2 (±16)^†^	35	0.69**	52 (±18)	39	0.45*	132 (±21)	40	0.07	169 (±26)	38	0.14	242 (±30)	41
C_s_	0.78**	−40 (±15)	36	0.63**	13 (±19)	40	0.46*	93 (±20)	41	0.11	129 (±25)	39	0.11	202 (±30)	42
D_s_	0.76**	−81 (±16)	36	0.60**	−27 (±20)	40	0.55**	51 (±19)	41	0.28	88 (±23)	39	0.23	160 (±28)	42
E_s_	0.68**	−129 (±16)	36	0.50*	−75 (±19)	40	0.71**	4 (±11)^†^	41	0.37	40 (±16)	39	0.31	113 (±23)	42
F_s_	0.67**	−149 (±17)	36	0.60**	−96 (±17)	40	0.77**	−17 (±9)	41	0.49*	19 (±13)	39	0.33	93 (±22)	42
G_s_	0.47*	−172 (±20)	36	0.44*	−119 (±19)	40	0.62**	−39 (±11)	41	0.60**	−3 (±11)^†^	39	0.40*	70 (±21)	42
I_s_	0.23	−196 (±24)	35	0.33	−143 (±22)	39	0.52**	−64 (±14)	40	0.42*	−27 (±14)	38	0.51**	46 (±20)	41
J_s_	−0.01	−209 (±28)	34	0.12	−156 (±26)	38	0.47*	−76 (±16)	39	0.53**	−40 (±14)	37	0.51**	32 (±20)	40
K_s_	0.13	−230 (±28)	36	0.20	−176 (±27)	40	0.54**	−97 (±17)	41	0.57**	−61 (±16)	39	0.42*	13 (±23)	42
L_s_	0.22	−267 (±26)	35	0.22	−214 (±26)	39	0.50**	−135 (±17)	40	0.69**	−99 (±14)	38	0.57**	−24 (±20)	41
M_s_	0.09	−319 (±34)	35	0.08	−266 (±34)	39	0.34	−185 (±27)	40	0.38	−150 (±26)	38	0.35	−75 (±29)	41

R = Pearsons Correlation (*p < 0.01; **p < 0.001). Diff: Mean difference between the time of occurrence of the fiducial point in the seismocardiogram and the physiologic event found in the ultrasound images (^†^indicates no significant difference in time). Points: The sets of ultrasound images and fiducial points used in the correlation and difference calculations.

**Table 5:** Correlations, Difference and number of points between Ultrasound Images And Seismocardiography in the Diastolic complex.

Fiducial Point	Aortic Valve Closure			Mitral Valve Opening			E-wave Peak		
	R	Diff (±SD) [ms]	Points	R	Diff (±SD) [ms]	Points	R	Diff (±SD) [ms]	Points
A_d_	0.87**	24 (±20)	33	0.81**	108 (±24)	32	0.80**	167 (±27)	33
B_d_	0.94**	−5 (±12)^†^	39	0.90**	79 (±17)	38	0.86**	139 (±22)	40
C_d_	0.93**	−25 (±12)	39	0.92**	58 (±15)	38	0.87**	118 (±21)	40
D_d_	0.93**	−38 (±12)	39	0.93**	46 (±15)	38	0.87**	106 (±21)	40
E_d_	0.90**	−64 (±14)	39	0.90**	19 (±17)	38	0.85**	79 (±23)	40
F_d_	0.85**	−92 (±18)	33	0.87**	−7 (±19)^†^	32	0.79**	53 (±27)	34
G_d_	0.86**	−111 (±19)	33	0.90**	−26 (±17)	32	0.81**	33 (±27)	34
H_d_	0.78**	−161 (±23)	38	0.86**	−77 (±20)	37	0.79**	−18 (±28)	39
I_d_	0.73**	−216 (±28)	38	0.76**	−132 (±28)	37	0.64**	−75 (±38)	39

R = Pearsons Correlation (*p < 0.01; **p < 0.001). Diff: Mean difference between the time of occurrence of the fiducial point in the seismocardiogram and the physiologic event found in the ultrasound images (^†^indicates no significant difference in time). Points: The sets of ultrasound images and fiducial points used in the correlation and difference calculations.

#### Onset of A-wave and Atrial Systole

The lowest difference between A-wave-onset and AS in the echocardiograms and any fiducial point in the SCG was found for point B_s_ with a mean difference of −2 ms (±16 ms). The correlation between the SCG fiducial point and the ultrasound image time location is 0.75 with p < 0.001.

#### Peak A-wave

The shortest time difference between the peak of the A-wave and the fiducial points was found for point C_s_ with a mean difference of 13 ms (±19 ms) and a correlation of 0.63 (p < 0.001).

#### Mitral Valve Closure

The mean difference between the fiducial points and the MC event in the images is lowest for fiducial point E_s_ with a mean difference = 4 ms (±11 ms). The correlation for this fiducial point is 0.71 with a p-value < 0.001.

#### Onset systolic profile and Aortic valve Opening

**Figure 3:**
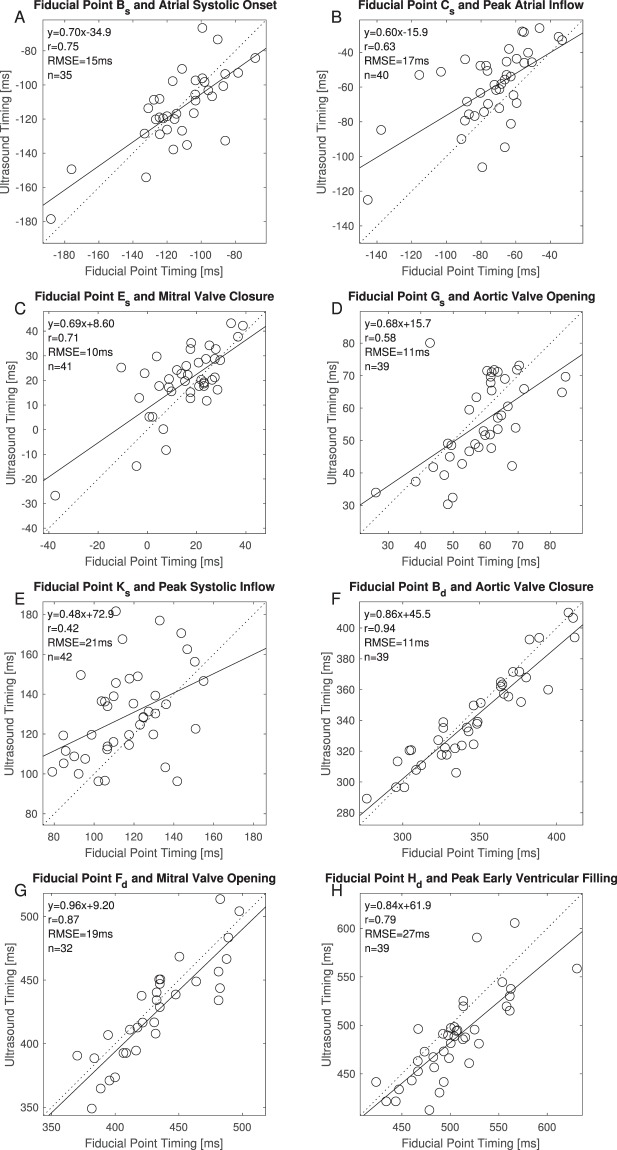
Correlation plots for the fiducial points and ultrasound images listed in Table 6.

The mean time point from the onset of the systolic profile in the tissue Doppler images and the aortic valve opening found on the pulsed Doppler flow images were correlated with the fiducial points from the systolic complex of the SCG. Fiducial point G_s_ has the lowest mean and absolute mean time difference to the mean time point (mean difference = −3 ms (±11 ms)). The correlation is 0.60; p < 0.001. Figure 2B is a visualization of one subject’s SCG. This subject is the outlier in Fig. 3D. If this subject is removed from the data set, the correlation between fiducial point G_s_ and Aortic Valve Opening is 0.70 instead of 0.60 – still with a p < 0.001.

#### Peak systolic inflow

The fiducial point K_s_ has the shortest mean time difference of 13 ms (±23 ms) to the event from the ultrasound image. The correlation is 0.42 (p < 0.01).

### Diastolic complex

Table 4 shows the correlation between fiducial points in the diastolic complex of the SCG and events found in the ultrasound images.

#### Aortic Valve Closure and end of systolic flow

The time point for AC was found in both the tissue Doppler image and the pulsed wave Doppler as the end of systolic flow. The shortest time difference between the fiducial point of the SCG and the time point for aortic valve closure is −5 ms (±12 ms) and is located at point B_d_. The correlation between the fiducial point and time point is 0.94 (p < 0.001).

#### Mitral Valve Opening and onset of E-wave

For the MO time point a combination of the tissue Doppler image and the onset of the E-wave in the pulsed wave Doppler was used. The shortest mean time difference of −7 ms (±19 ms) is to the fiducial point S_d_. The correlation is 0.87 (p < 0.001).

#### E-wave peak

For the peak of the E-wave the fiducial point with the shortest time difference (mean difference = −18 ms (±28 ms)) was U_s_ with a correlation of 0.79 (p < 0.001).

**Figure 4:**
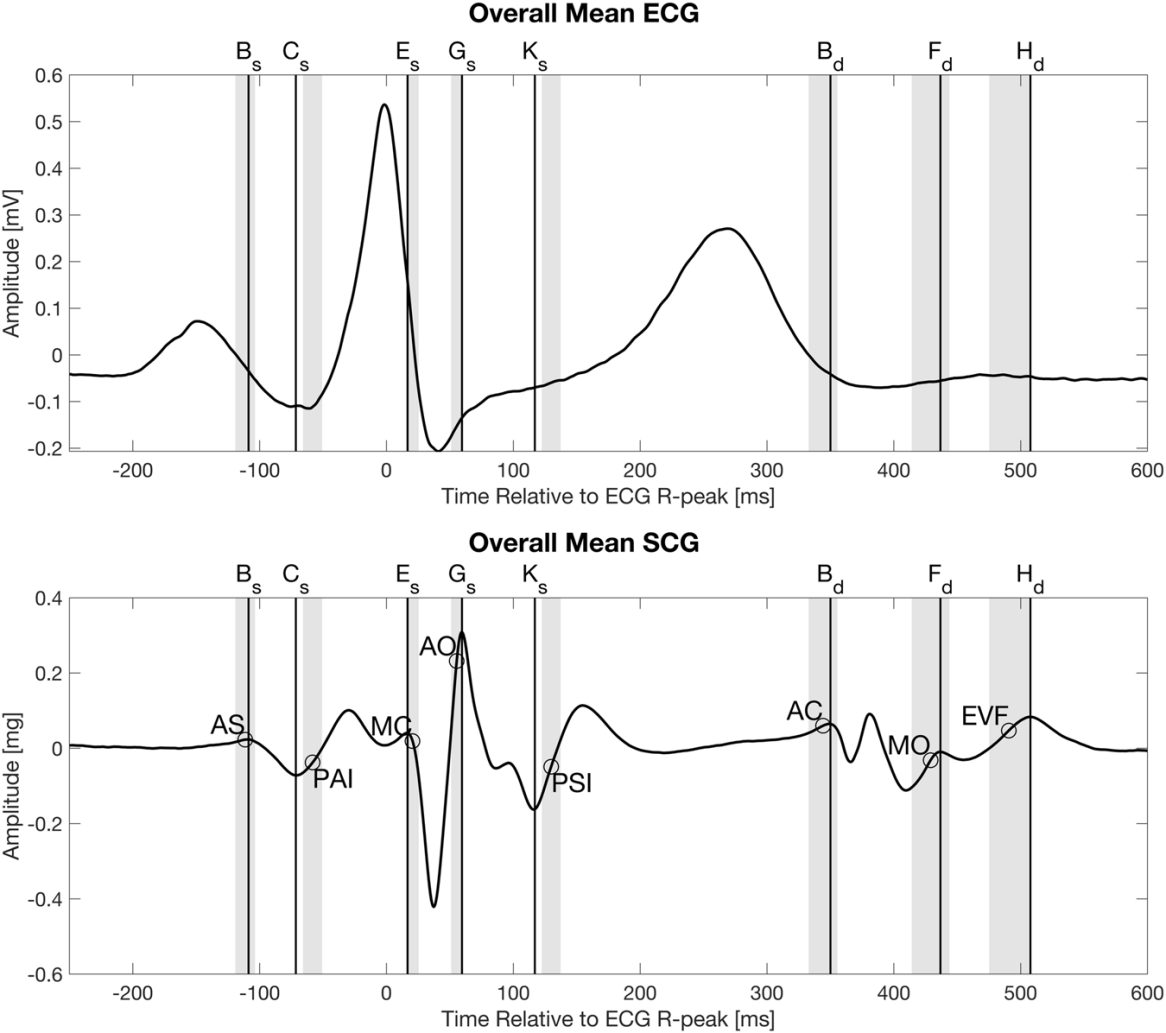
Overall mean electrocardiogram and seismocardiogram signal from 34 subjects. The circles indicate the mean location of the following physiologic events found in ultrasound images, from left: Atrial systole (AS), peak atrial inflow (PAI), mitral valve closure (MC), aortic valve opening (AO), peak systolic inflow (PSI), aortic valve closing (AC), mitral valve opening (MO), early ventricular filling (EVF). The grey areas indicate the 95% confidence intervals of the means for the physiologic events found in the ultrasound images.

All the points evaluated above with a short time interval between a particular fiducial point and the corresponding physiologic event in the ultrasound image, are listed in Table 4. In the table the mean times of occurrence of both the fiducial points from the SCG signal and the times of occurrence of the physiologic event are also listed. A visual representation of the data from the eight combinations of fiducial points and ultrasound locations is shown in Fig. 4. Both Fig. 3 and Table 6 also presents the beta coefficients for the linear regression between fiducial points and ultrasound locations.

The fiducial points B_s_, C_s_, D_s_, E_s_, F_s_, G_s_, K_s_, B_d_, F_d_ and H_d_ were used as fixed points in a time warping approach to define one overall mean SCG and ECG signal. The signals were segmented into parts based on the aforementioned fiducial points. A segment from signal start to fiducial point B_s_ and a segment from H_d_ to the end of the signal was also created, making a total of 11 segments across those 34 signals that contained all the fiducial points. For each segment the mean length was calculated, and all segments were resampled to fit this length with Matlab’s “spline” function. Thus, following this step all signal parts of the same segment were of the same length. Using the original length of each signal part and the new length of the same part a “resampling factor” was calculated and used to reposition the location of the ultrasound event to the new length of the signal parts. An overall mean SCG signal was computed based on the parts of signals, see Fig. 4. The 95% confidence interval of the mean was calculated based on the relocated ultrasound locations. The SCG signal in Fig. 4 consists of signals aligned to either the systolic or diastolic complex and then connected at approx. 250 ms after the ECG R-peak.

## Discussion

In this work we propose a nomenclature for the various waves and wave complexes in the normal seismocardiogram and we have identified the main physiological events in the cardiac cycle relative to those waves. A total of 42 SCG signals were manually annotated and the times of occurrence of the fiducial points were correlated to the occurrences of the physiological events in ultrasound images.

Current literature visualizes cardiac events as occurring at the local extrema of the SCG signal^9,10,13–16,21^. Only the original SCG paper describes cardiac events taking place on the slopes of the signal and not at the fiducial points^1^. Our results points towards the same as Mounsey’s: the physiologic event does not always occur at the fiducial point but before or after.

Fiducial point B_s_ is related to the atrial systole according to our results, see Table 6. In the 1957 paper by Mounsey the atrial systole is described as an initial downward deflection followed by an upward deflection. This is similar to our results, where fiducial point B_s_ marks the beginning of the negative slope describing the atrial systole in Mounseys paper. Likewise, the following positive slope in Mounseys paper is the same as the positive slope from our results. This corresponds to the positive slope following fiducial point C_s_. According to the ultrasound images the onset of the echocardiographic A-wave (the atrial systole - AS) is just prior to B_s_ whereas the beginning of the slope following fiducial point C_s_ marks the peak of the A-wave (peak atrial inflow - PAI). The acceleration deflection corresponds to an initial outwards deflection in displacement followed by an inwards deflection (Note: the displacement signal is obtained based on the signal from Fig. 4 using the Omega-Arithmetic). The outwards displacement could be caused by the atria contracting into a rounded shape as the pressure inside them builds. The inwards displacement could be described as the flattening of the atria when blood is forced into the ventricles.

**Table 6:** Overview of fiducial points with shortest time difference and highest correlation to physiologic events found in the ultrasound images.

Physiologic Event	Fiducial Point	R	Diff (±SD) [ms]	Fiducial Mean Location (±SD) [ms]	Ultrasound Image Mean Location (±SD) [ms]	Beta coefficient linear regression
Atrial Systolic Onset	B_s_	0.75**	−2 (±16)^†^	−111 (±24)	−113 (±22)	0.70
Peak Atrial Inflow	C_s_	0.63**	13 (±19)	−73 (±23)	−61 (±22)	0.60
Mitral Valve Closure	E_s_	0.71*	4 (±11)^†^	16 (±14)	19 (±15)	0.69
Aortic Valve Opening	G_s_	0.60**	−3 (±11)^†^	59 (±11)	55 (±13)	0.68
Peak Systolic Inflow	K_s_	0.42*	13 (±23)	116 (±20)	128 (±22)	0.48
Aortic Valve Closure	B_d_	0.94**	−5 (±12)^†^	348 (±37)	342 (±30)	0.86
Mitral Valve Opening	F_d_	0.87**	−7 (±19)^†^	437 (±37)	424 (±38)	0.96
Peak Early Ventricular Filling	H_d_	0.79**	−18 (±28)	505 (±41)	485 (±43)	0.84

R = Pearsons Correlation. *p < 0.01; **p < 0.001. Diff: Mean difference between fiducial point in the seismocardiogram and physiologic event found in the ultrasound image (^†^indicates no significant difference in time).

Fiducial point E_s_ is situated around the location of the mitral valve closure point according to the ultrasound images. Mounsey reports the start of the outwards deflection to correspond to the mitral valve closure. This deflection starts before the ECG R-peak, together with the first heart sound. This corresponds to the valley prior to our fiducial point E_s_. We find this event to happen at 19 (±15) ms after the R-peak, thus not at the same location as suggested by Mounsey.

Following E_s_ is the isovolumetric contraction segment. Before the aortic valve opens the heart start to contract to build enough pressure in the left ventricle to exceed the pressure in the aorta. The acceleration is first negative, then positive. The corresponding displacement signal is an outward deflection. This could be due to a rounding of the ventricles as they contract, and pressure is built. The outward displacement deflection stops as the acceleration deflection is at the valley prior to fiducial point G_s_. From this point the positive acceleration describes the inward displacement, possibly due to the blood being forced out of the ventricles thus causing the heart to flatten. This corresponds with the finding by Mounsey.

The positive acceleration starting at fiducial point K_s_ is caused by an inward displacement during what Mounsey calls “reduced ejection” period^1^.

Fiducial point B_d_ is associated with the closing of the aortic valve^1^. Just before this event a positive deflection of displacement starts. This deflection continues to fiducial point E_d_. The outward and following inward displacement could be due to the forces created by the closure of the aortic valve. Blood in the aorta forces the valves to close causing a movement of the heart towards the apex of the heart. Mounsey explains this further by comparing two recordings of the SCG, one from the upper sternal area and one from the apical area. The deflections in the two recordings are opposite to each other. In the upper sternal area the displacement was upward instead of downward, resulting from an inward displacement over the area of the valves^1^.

Fiducial Point F_d_ is located in the interval containing of the mitral valve opening. The positive acceleration leading up to this fiducial point is associated with an inward displacement deflection. Mounsey explains these changes with a possible change in position of the heart in this period.

The last fiducial point H_d_ is associated with early ventricular filling. This positive deflection in acceleration is associated with a positive displacement possibly caused by blood filling the ventricles as the mitral valve and tricuspid valves opens. Mounsey proposes the same explanation for this positive displacement deflection.

For the three locations peak atrial inflow, peak systolic inflow and peak early ventricular filling the difference between these physiologic events and the closest fiducial points were significantly different from zero.

With regard to accurately determining the events in the ultrasound images it should be noted that this process is not perfect. The quality of the profile of the flow or tissue movement was varying, such that an accurate determination of the occurrence of the events still is a subjective task. In this study, the operator was blinded for the corresponding SCG signal when determining the events in the ultrasound image. This eliminates the bias towards selecting events that could fit any hypothesis the operator might have with regard to where to locate echocardiographic events relative to the SCG.

A different approach for a study like this, could be to use homologous time points as described by Piras *et al*.^26,27^. This would result in a relation between the SCG signal and the evolution of the shape of the contractions, instead of the locations defined in this paper.

### Study limitations

The main limitation in this study is the low temporal resolution of the ultrasound modalities compared to that of the SCG signal. When analyzing the ultrasound images, the low resolution limits the operator’s ability to accurately mark the events. Moreover, the ultrasound images consist of only 1–4 consecutive beats whereas the SCG signals are composed of more than 20 beats, thus a more general representation of the SCG signal from a subject compared to the ultrasound image. This could lead to inconsistency between the SCGs and the ultrasound images due to variations of the heartbeat, for instance introduced by the respiration component of the signals.

### Conclusion

We have defined fiducial points in the SCG and associated them with physiologic events obtained from cardiac ultrasound images and we found a consistent relationship between SCG fiducial points and the events. Furthermore, this study introduces a new location for the MO event.

## Electronic Supplementary Material

All SCG Signals from Dataset

## References

[1] Mounsey P (1957). Praecordial ballistocardiography. Br. Heart J..

[2] Elliott RV, Packard RGAY, Kyrazis DT (1954). Acceleration Ballistocardiography: Design, Construction, and Application of a new instrument. Circulation.

[3] Bozhenko BS (1961). Seismocardiography–a new method in the study of functional conditions of the heart. Ter. Arkh..

[4] Baevsky R, Egorov A, Kazarian L (1964). Metodika seismokardiografii. Kardiologia.

[5] Henderson Y (1905). The mass-movements of the circulation as shown by a recoil curve. Am. J. Physiol..

[6] Baevskii, R. M. Physiological methods in astronautics. No. FTD-MT-66-42. FOREIGN TECHNOLOGY DIV WRIGHT-PATTERSONAFB OH, 1966.

[7] Zanetti J, Salerno D (1990). Seismocardiography: A new technique for recording cardiac vibrations. concept, method, and initial observations. J. Cardiovasc. Technol..

[8] Zanetti, J. M. & Tavakolian, K. Seismocardiography: Past, Present and Future. *Engineering in Medicine and Biology Society (EMBC), 2013 35th Annual International Conference of the IEEE.* 7004–7007 (2013). 10.1109/EMBC.2013.6611170 24111357

[9] Inan O (2014). Ballistocardiography and Seismocardiography: A Review of Recent Advances. IEEE J. Biomed. Heal. Informatics.

[10] Wilson RA, Bamrah VS, Lindsay J, Schwaiger M, Morganroth J (1993). Diagnostic accuracy of seismocardiography compared with electrocardiography for the anatomic and physiologic diagnosis of coronary artery disease during exercise testing. Am. J. Cardiol..

[11] Di Rienzo, M. *et al*. A wearable system for the seismocardiogram assessment in daily life conditions. *Proc. Annu. Int. Conf. IEEE Eng. Med. Biol. Soc. EMBS*, 4263–4266, 10.1109/IEMBS.2011.6091058 (2011). 10.1109/IEMBS.2011.6091058 22255281

[12] Di Rienzo M (2013). Wearable seismocardiography: Towards a beat-by-beat assessment of cardiac mechanics in ambulant subjects. Auton. Neurosci. Basic Clin..

[13] Tavakolian, K. *Characterization and analysis of seismocardiogram for estimation of hemodynamic parameters*. (Applied Science: School of Engineering Science, 2010).

[14] Zanetti, J. M., Poliac, M. O. & Crow, R. S. Seismocardiography: waveform identification and noise analysis. *Computers in Cardiology 1991*, Proceedings. 49–52 (1991).

[15] Zanetti, J. M. & Salerno, D. M. Seismocardiography: a technique for recording precordial acceleration. *Computer-Based Medical Systems*. Proceedings of the Fourth Annual IEEE Symposium 4–9 (1991).

[16] Khosrow-Khavar F (2014). Automatic Annotation of Seismocardiogram with High Frequency Precordial Accelerations. IEEE J. Biomed. Heal. informatics.

[17] Yang C, Tavassolian N (2017). Pulse Transit Time Measurement Using Seismocardiogram, Photoplethysmogram, and Acoustic Recordings: Evaluation and Comparison. IEEE J. Biomed. Heal. Informatics.

[18] Mukkamala R (2015). Toward ubiquitous blood pressure monitoring via pulse transit time: theory and practice. IEEE Trans. Biomed. Eng..

[19] Di Rienzo, M., Vaini, E. & Lombardi, P. Use of seismocardiogram for the beat-to-beat assessment of the Pulse Transit Time: A pilot study. *Engineering in Medicine and Biology Society (EMBC)*, 2015 37th Annual International Conference of the IEEE 7184–7187 (2015). 10.1109/EMBC.2015.7320049 26737949

[20] Soerensen K (2017). Challenges in Using Seismocardiography for Blood Pressure Monitoring. Computing in Cardiology.

[21] Crow RS, Hannan P, Jacobs D, Hedquist L, Salerno DM (1994). Relationship between seismocardiogram and echocardiogram for events in the cardiac cycle. Am. J. noninvasive Cardiol..

[22] Jensen, A. S. *et al*. Effects of Cardiac Resynchronization Therapy on the First Heart Sound Energy. 29–32 (2014).

[23] Bulwer, B. & Rivero, J. *Echocardiography Pocket Guide: The Transthoracic Examination (Echocardiography Pocket Guides)*. (Jones & Bartlett Publishers, 2010).

[24] Otto, Catherine M. *Textbook of Clinical Echocardiography: Expert Consult - Online and Print, 4e*. (Saunders, 2009).

[25] Biering-Sørensen T (2016). Cardiac time intervals by tissue doppler imaging M-mode: Normal values and association with established echocardiographic and invasive measures of systolic and diastolic function. PLoS One.

[26] Piras, P. *et al*. Homeostatic Left Heart integration and disintegration links atrio-ventricular covariations dyshomeostasis in Hypertrophic Cardiomyopathy. **7**, 6257 (2017). 10.1038/s41598-017-06189-w PMC5524707 28740203

[27] Piras, P. *et al*. Left Atrial trajectory impairment in Hypertrophic Cardiomyopathy disclosed by Geometric Morphometrics and Parallel Transport. **6**, 34906 (2016). 10.1038/srep34906 PMC5054674 27713503

