# Author Correction: Definition of Fiducial Points in the Normal Seismocardiogram

**DOI:** 10.1038/s41598-020-68363-x

**Published:** 2020-09-04

**Authors:** Kasper Sørensen, Samuel E. Schmidt, Ask S. Jensen, Peter Søgaard, Johannes J. Struijk

**Affiliations:** 1 Department of Health Science and Technology, Aalborg University, 9220 Aalborg, Denmark; 2 Department of Cardiology, Aalborg University Hospital, 9000 Aalborg, Denmark

Correction to:* Scientific Reports* 10.1038/s41598-018-33675-6, published online 18 October 2018

The original version of this Article contained an error in the scaling of the y-axis in seismocardiogram figures that resulted in the amplitudes being reported incorrectly. This error affected Figures 1, 2 and 4, and the supplementary figures.

In addition, in the original version of this Article, the term ‘peak systolic inflow’ was incorrectly used instead of ‘peak systolic outflow’.

In the Abstract,

“The smallest mean differences (±SD) between the eight events found in the ultrasound images and the fiducial points, together with their correlation coefficients (r) were: atrial systolic onset: − 2 (±16) ms, r = 0.75 (p < 0.001); peak atrial inflow: 13 (±19) ms, r = 0.63 (p < 0.001); mitral valve closure: 4 (±11) ms, r = 0.71 (p < 0.01); aortic valve opening: − 3 (±11) ms, r = 0.60 (p < 0.001); peak systolic inflow: 13 (±23) ms, r = 0.42 (p < 0.01); aortic valve closure: − 5 (±12) ms, r = 0.94 (p < 0.001); mitral valve opening: − 7 (±19) ms, r = 0.87 (p < 0.001) and peak early ventricular filling: − 18 (±28 ms), r = 0.79 (p < 0.001).”

now reads:

“The smallest mean differences (±SD) between the eight events found in the ultrasound images and the fiducial points, together with their correlation coefficients (r) were: atrial systolic onset: − 2 (±16) ms, r = 0.75 (p < 0.001); peak atrial inflow: 13 (±19) ms, r = 0.63 (p < 0.001); mitral valve closure: 4 (±11) ms, r = 0.71 (p < 0.01); aortic valve opening: − 3 (±11) ms, r = 0.60 (p < 0.001); peak systolic outflow: 13 (±23) ms, r = 0.42 (p < 0.01); aortic valve closure: − 5 (±12) ms, r = 0.94 (p < 0.001); mitral valve opening: − 7 (±19) ms, r = 0.87 (p < 0.001) and peak early ventricular filling: − 18 (±28 ms), r = 0.79 (p < 0.001).”

In the Results, the subheading “Peak systolic inflow” now reads “Peak systolic outflow”.

In the legend for Figure 4,

“The circles indicate the mean location of the following physiologic events found in ultrasound images, from left: Atrial systole (AS), peak atrial inflow (PAI), mitral valve closure (MC), aortic valve opening (AO), peak systolic inflow (PSI), aortic valve closing (AC), mitral valve opening (MO), early ventricular filling (EVF)”

now reads:

“The circles indicate the mean location of the following physiologic events found in ultrasound images, from left: Atrial systole (AS), peak atrial inflow (PAI), mitral valve closure (MC), aortic valve opening (AO), peak systolic outflow (PSO), aortic valve closing (AC), mitral valve opening (MO), early ventricular filling (EVF)”

In the Discussion,

“For the three locations peak atrial inflow, peak systolic inflow and peak early ventricular filling the difference between these physiologic events and the closest fiducial points were significantly different from zero.”

now reads:

“For the three locations peak atrial inflow, peak systolic outflow and peak early ventricular filling the difference between these physiologic events and the closest fiducial points were significantly different from zero.”

Finally, this error also affected Figure 3 and Tables 4 and 6, which have now been corrected in the original Article.

The original Figures 1, 2, 3, 4 and Tables 4 and 6 are included below. The original Supplementary Information accompanies this correction.

**Figure 1:**
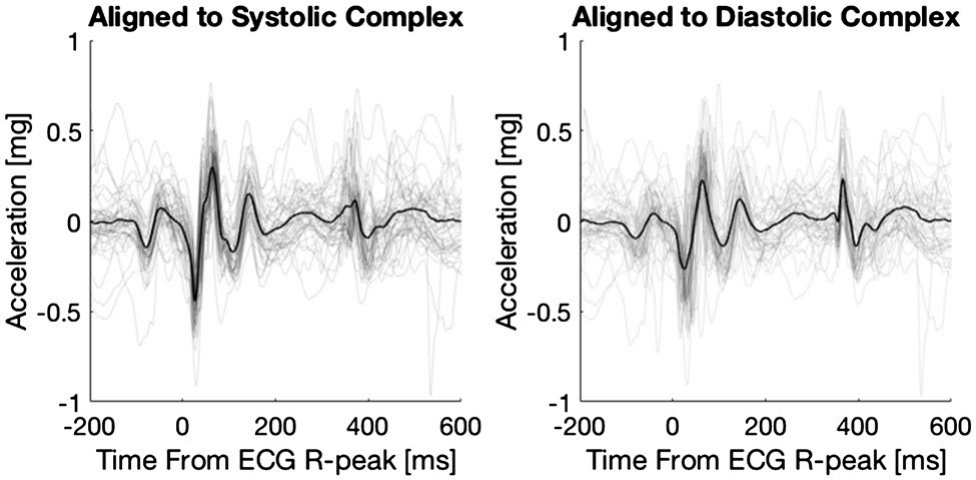
A seismocardiographic signal from one subject aligned to the ECG R-peak (systolic complex) and to the diastolic complex respectively. Individual beats are plotted in grey, the mean beats are plotted in black.

**Figure 2:**
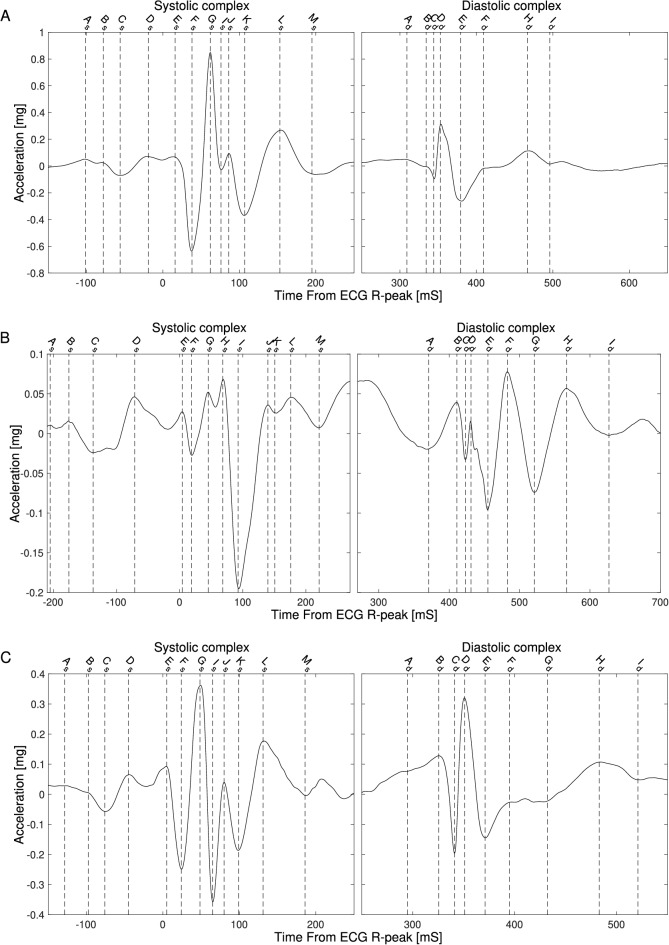
SCG signals from subject 09 (**A**), 15 (**B**) and 22 (**C**) with labeling of the fiducial points in the two complexes.

**Figure 3:**
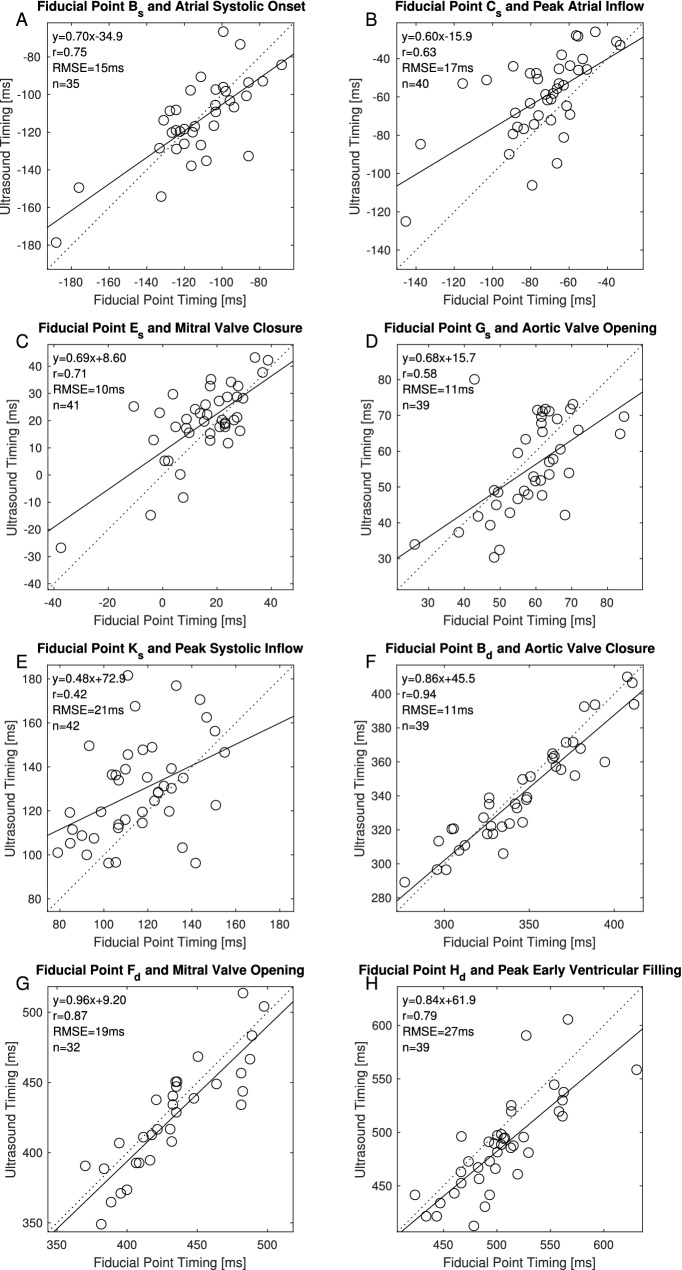
Correlation plots for the fiducial points and ultrasound images listed in Table 6.

**Figure 4:**
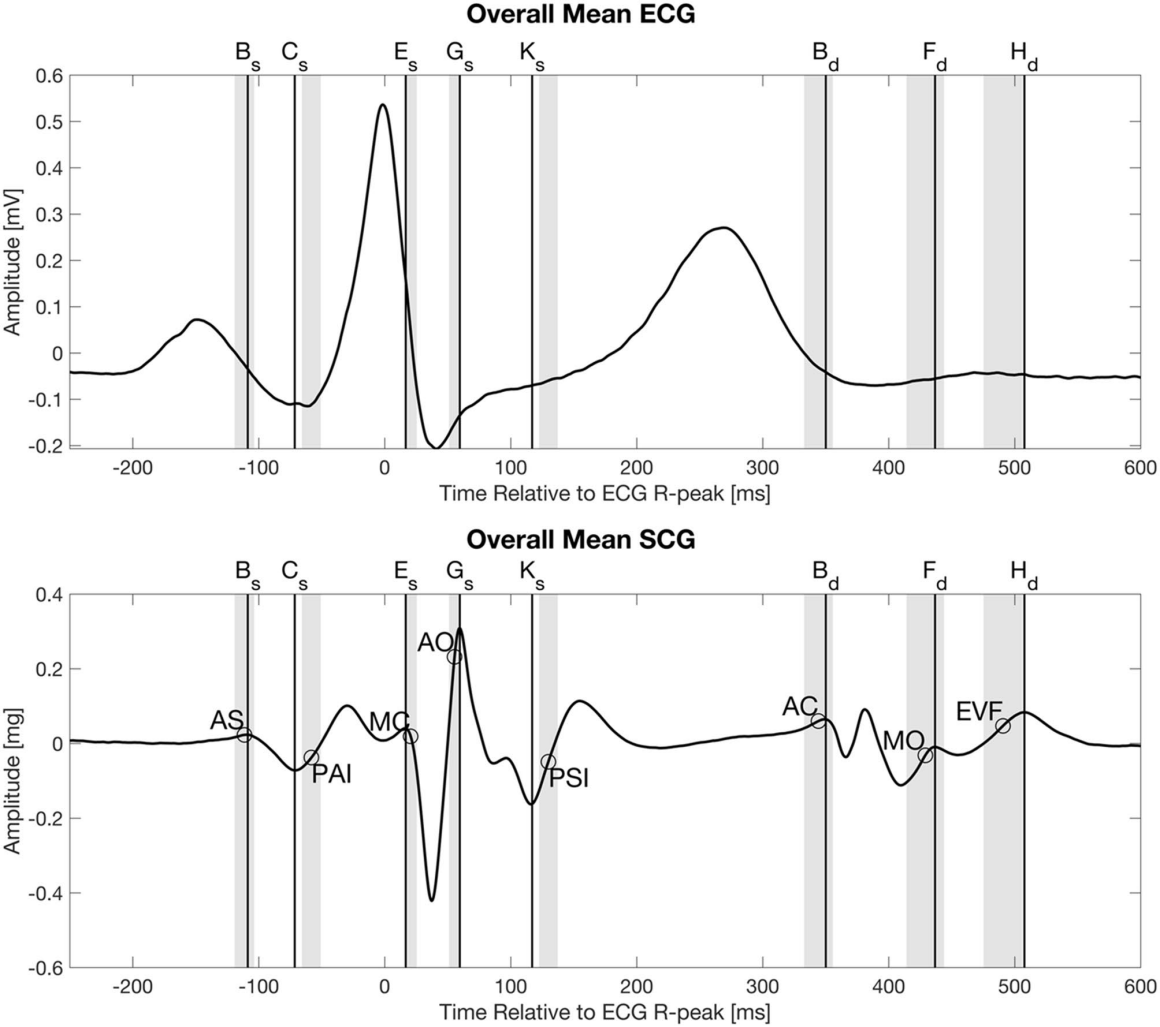
Overall mean electrocardiogram and seismocardiogram signal from 34 subjects. The circles indicate the mean location of the following physiologic events found in ultrasound images, from left: Atrial systole (AS), peak atrial inflow (PAI), mitral valve closure (MC), aortic valve opening (AO), peak systolic inflow (PSI), aortic valve closing (AC), mitral valve opening (MO), early ventricular filling (EVF). The grey areas indicate the 95% confidence intervals of the means for the physiologic events found in the ultrasound images.

**Table 4:** Correlations, difference and number of points between ultrasound images and seismocardiography systolic complex

Fiducial point	Onset atrial systole			Peak A-wave			Mitral valve closure			Aortic valve opening			Peak systolic inflow		
	R	Diff (± SD) [ms]	Points	R	Diff (± SD) [ms]	Points	R	Diff (± SD) [ms]	Points	R	Diff (± SD) [ms]	Points	R	Diff (± SD) [ms]	Points
B_s_	0.75**	− 2 (± 16)^†^	35	0.69**	52 (± 18)	39	0.45*	132 (± 21)	40	0.07	169 (± 26)	38	0.14	242 (± 30)	41
C_s_	0.78**	− 40 (± 15)	36	0.63**	13 (± 19)	40	0.46*	93 (± 20)	41	0.11	129 (± 25)	39	0.11	202 (± 30)	42
D_s_	0.76**	− 81 (± 16)	36	0.60**	− 27 (± 20)	40	0.55**	51 (± 19)	41	0.28	88 (± 23)	39	0.23	160 (± 28)	42
E_s_	0.68**	− 129 (± 16)	36	0.50*	− 75 (± 19)	40	0.71**	4 (± 11)^†^	41	0.37	40 (± 16)	39	0.31	113 (± 23)	42
F_s_	0.67**	− 149 (± 17)	36	0.60**	− 96 (± 17)	40	0.77**	− 17 (± 9)	41	0.49*	19 (± 13)	39	0.33	93 (± 22)	42
G_s_	0.47*	− 172 (± 20)	36	0.44*	− 119 (± 19)	40	0.62**	− 39 (± 11)	41	0.60**	− 3 (± 11)^†^	39	0.40*	70 (± 21)	42
I_s_	0.23	− 196 (± 24)	35	0.33	− 143 (± 22)	39	0.52**	− 64 (± 14)	40	0.42*	− 27 (± 14)	38	0.51**	46 (± 20)	41
J_s_	− 0.01	− 209 (± 28)	34	0.12	− 156 (± 26)	38	0.47*	− 76 (± 16)	39	0.53**	− 40 (± 14)	37	0.51**	32 (± 20)	40
K_s_	0.13	− 230 (± 28)	36	0.20	− 176 (± 27)	40	0.54**	− 97 (± 17)	41	0.57**	− 61 (± 16)	39	0.42*	13 (± 23)	42
L_s_	0.22	− 267 (± 26)	35	0.22	− 214 (± 26)	39	0.50**	− 135 (± 17)	40	0.69**	− 99 (± 14)	38	0.57**	− 24 (± 20)	41
M_s_	0.09	− 319 (± 34)	35	0.08	− 266 (± 34)	39	0.34	− 185 (± 27)	40	0.38	− 150 (± 26)	38	0.35	− 75 (± 29)	41

R = Pearsons Correlation (* = p < 0.01; ** = p < 0.001). Diff: Mean difference between the time of occurrence of the fiducial point in the seismocardiogram and the physiologic event found in the ultrasound images (^†^indicates no significant difference in time). Points: The sets of ultrasound images and fiducial points used in the correlation and difference calculations

**Table 6:** Overview of fiducial points with shortest time difference and highest correlation to physiologic events found in the ultrasound images

Physiologic event	Fiducial point	R	Diff (± SD) [ms]	Fiducial mean location (± SD) [ms]	Ultrasound image mean location (± SD) [ms]	Beta coefficient linear regression
Atrial systolic onset	B_s_	0.75**	− 2 (± 16) ^†^	− 111 (± 24)	− 113 (± 22)	0.70
Peak atrial inflow	C_s_	0.63**	13 (± 19)	− 73 (± 23)	− 61 (± 22)	0.60
Mitral valve closure	E_s_	0.71*	4 (± 11)^†^	16 (± 14)	19 (± 15)	0.69
Aortic valve opening	G_s_	0.60**	− 3 (± 11)^†^	59 (± 11)	55 (± 13)	0.68
Peak systolic inflow	K_s_	0.42*	13 (± 23)	116 (± 20)	128 (± 22)	0.48
Aortic valve closure	B_d_	0.94**	− 5 (± 12) ^†^	348 (± 37)	342 (± 30)	0.86
Mitral valve opening	F_d_	0.87**	− 7 (± 19) ^†^	437 (± 37)	424 (± 38)	0.96
Peak early ventricular filling	H_d_	0.79**	− 18 (± 28)	505 (± 41)	485 (± 43)	0.84

R = Pearsons Correlation. * = p < 0.01; ** = p < 0.001. Diff: Mean difference between fiducial point in the seismocardiogram and physiologic event found in the ultrasound image (^†^indicates no significant difference in time)

These errors have now been corrected in the HTML and PDF versions of this Article and in the Supplementary Information that accompanies this Article.

## Supplementary information

Original Supplementary Information

