# Supplementary material for: Definition of Fiducial Points in the Normal Seismocardiogram
Source: Sci Rep. 2018 Oct 18;8:15455. doi: 10.1038/s41598-018-33675-6 (PMC6193995; doi:10.1038/s41598-018-33675-6)
Supplement: Supplementary file 1 — All SCG Signals from Dataset [file 41598_2018_33675_MOESM1_ESM.pdf]

Definition of Fiducial Points in the Normal Seismocardiogram

Kasper Sørensen[1,*], Samuel E. Schmidt[1], Ask S. Jensen[1], Peter Søgaard[2] and Johannes J. Struijk[1]

[1]Aalborg University, Department of Health Science and Technology, Aalborg, 9220, Denmark
[2]Aalborg University Hospital, Department of Cardiology, Aalborg, 9000, Denmark

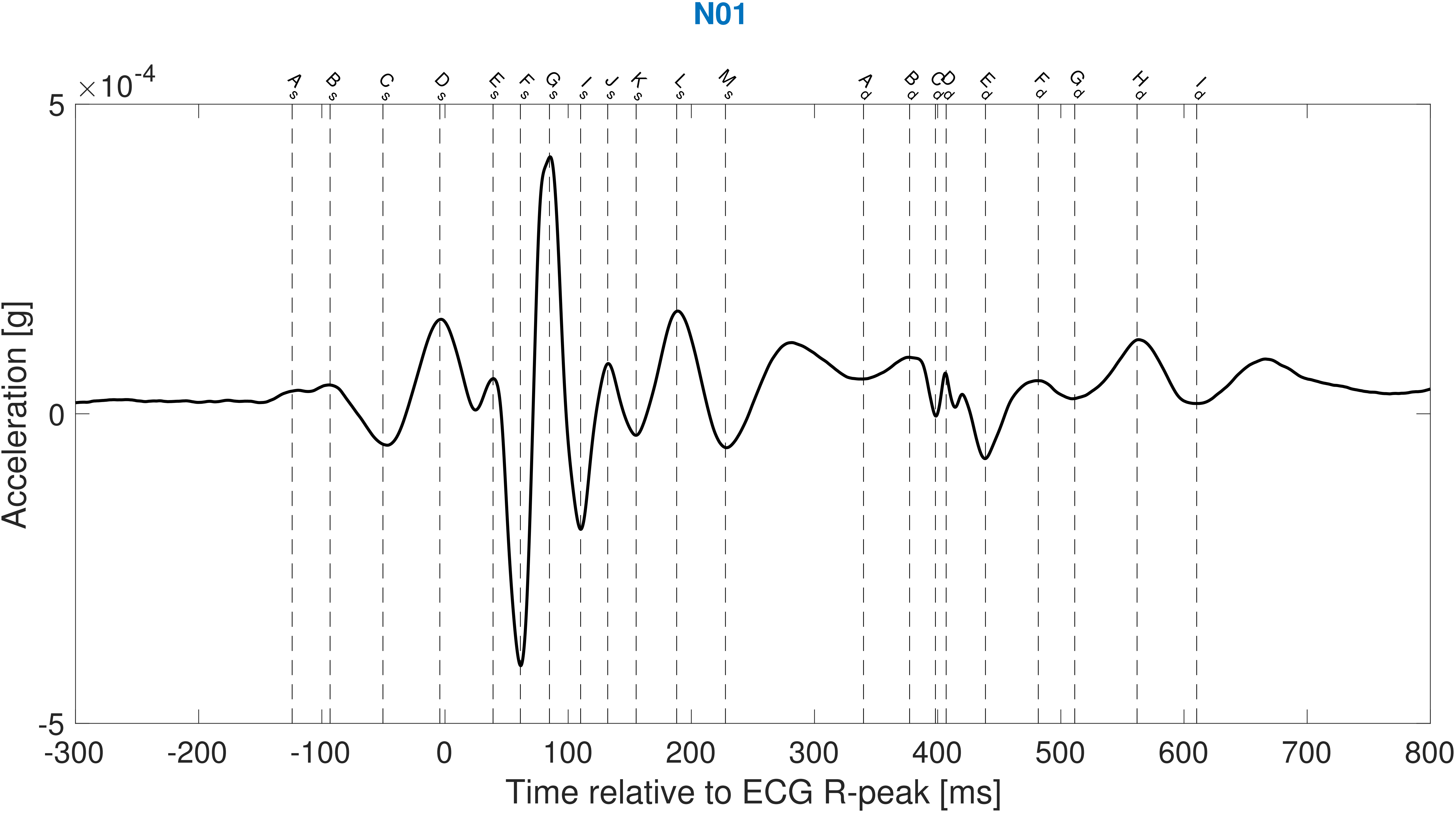

N01
×10^-4
5
0
-5
Acceleration [g]
-300
-200
-100
0
100
200
300
400
500
600
700
800
Time relative to ECG R-peak [ms]
A s
B s
C s
D s
E s
F s
G s
I s
J s
K s
L s
M s
A d
B d
C d
D d
E d
F d
G d
H d
I d

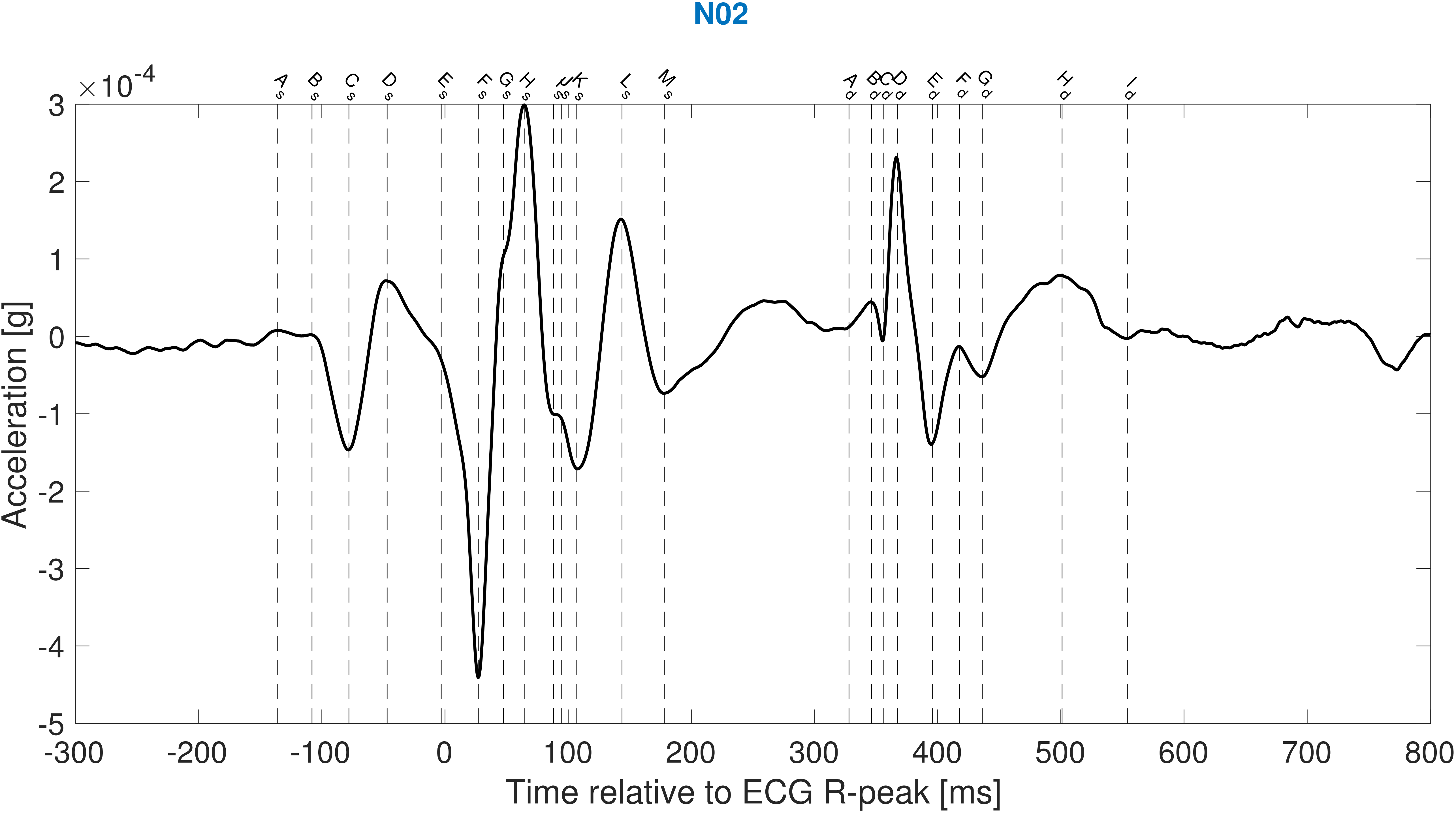
N02
Acceleration [g]
$\times 10^{-4}$
Time relative to ECG R-peak [ms]
A_s B_s C_s D_s E_s F_s G_s H_s K_s L_s M_s
A_d B_d C_d D_d E_d F_d G_d H_d I_d

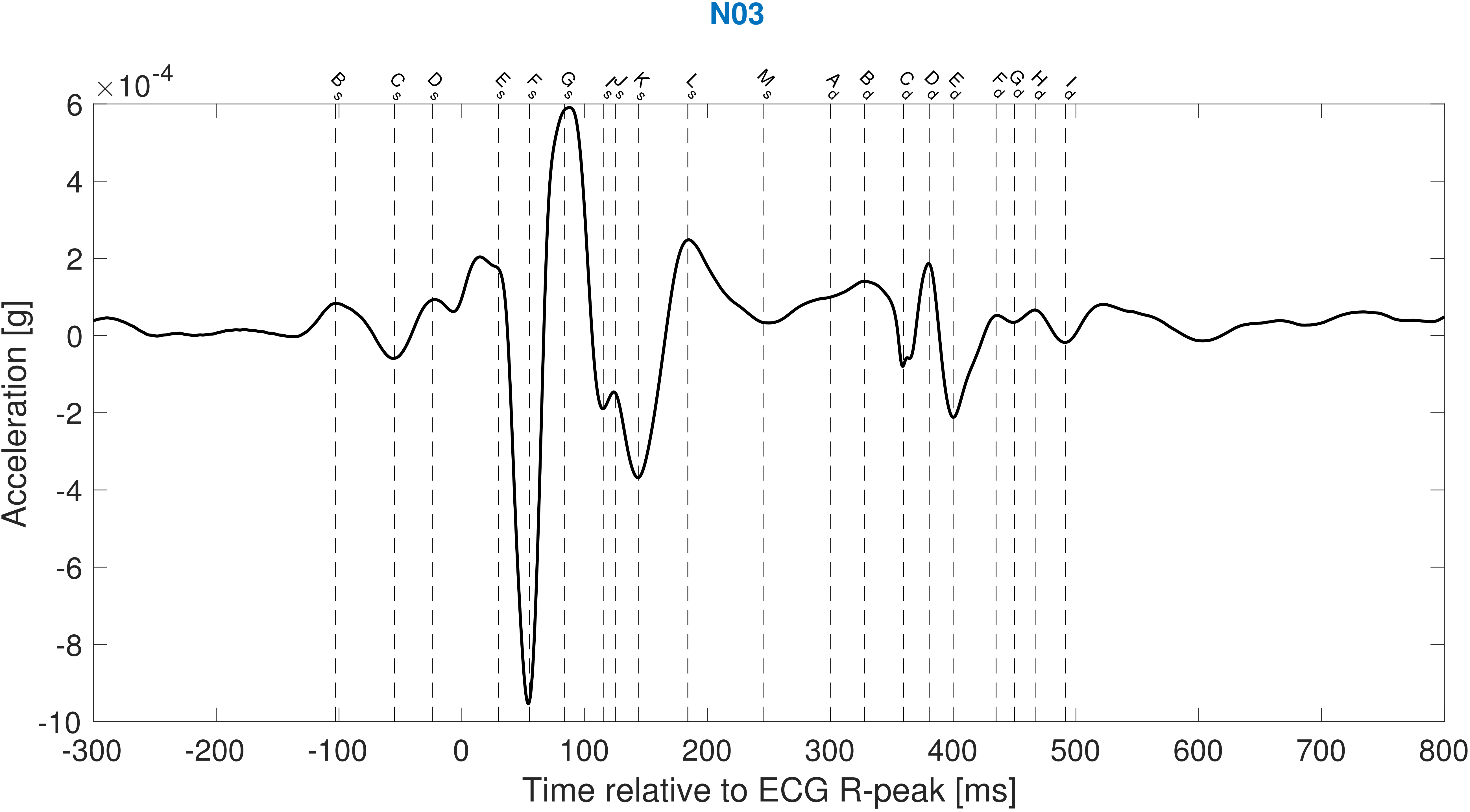

N03
×10^-4
Acceleration [g]
Time relative to ECG R-peak [ms]
6
4
2
0
-2
-4
-6
-8
-10
-300
-200
-100
0
100
200
300
400
500
600
700
800
Bs
Cs
Ds
Es
Fs
Gs
Is
Js
Ks
Ls
Ms
Ad
Bd
Cd
Dd
Ed
Fd
Gd
Hd
Id

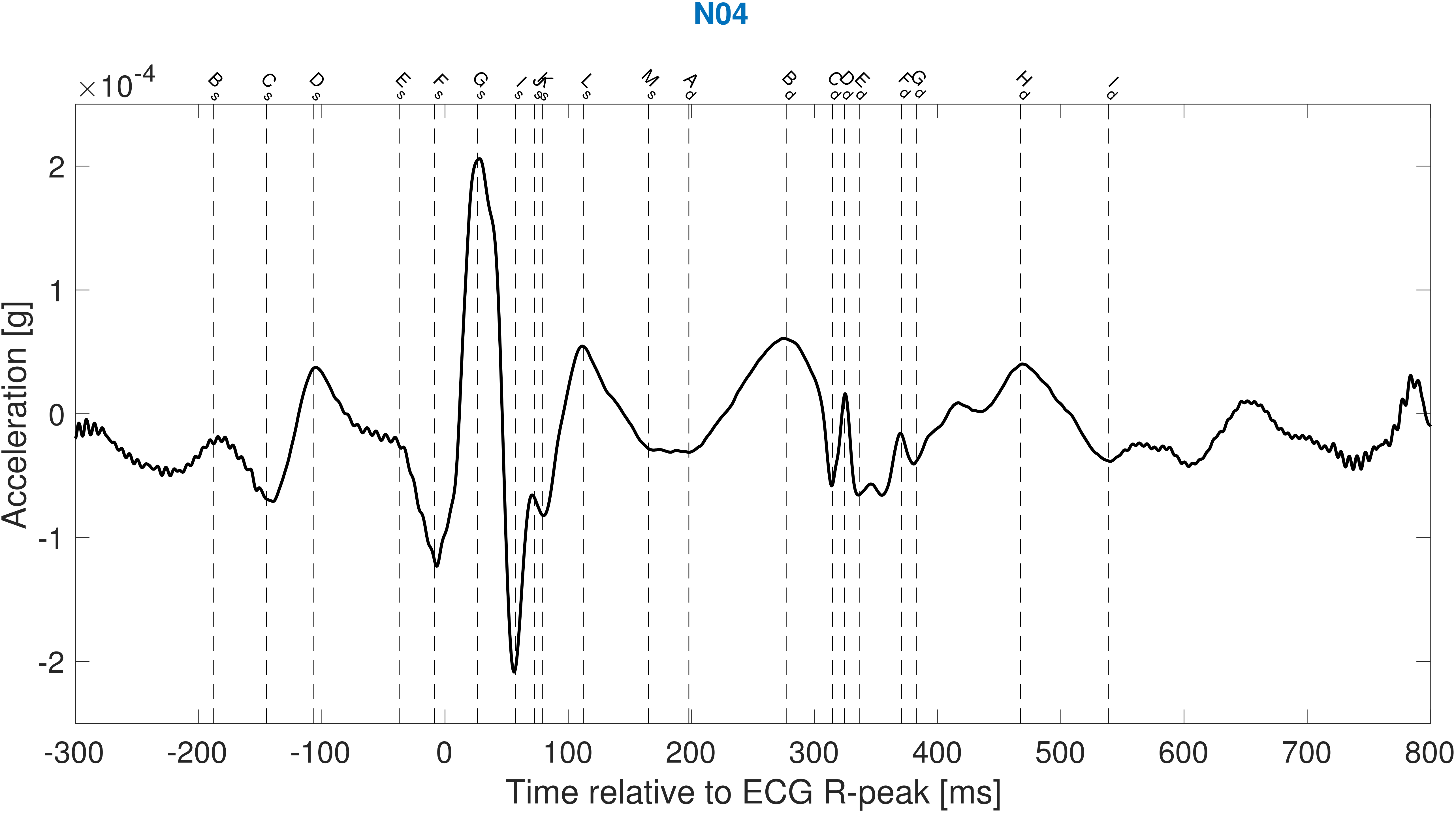
N04
×10^-4
Acceleration [g]
Time relative to ECG R-peak [ms]
-300
-200
-100
0
100
200
300
400
500
600
700
800
2
1
0
-1
-2
Bs
Cs
Ds
Es
Fs
Gs
Is
Js
Ks
Ls
Ms
Ad
Bd
Cd
Dd
Ed
Fd
Gd
Hd
Id

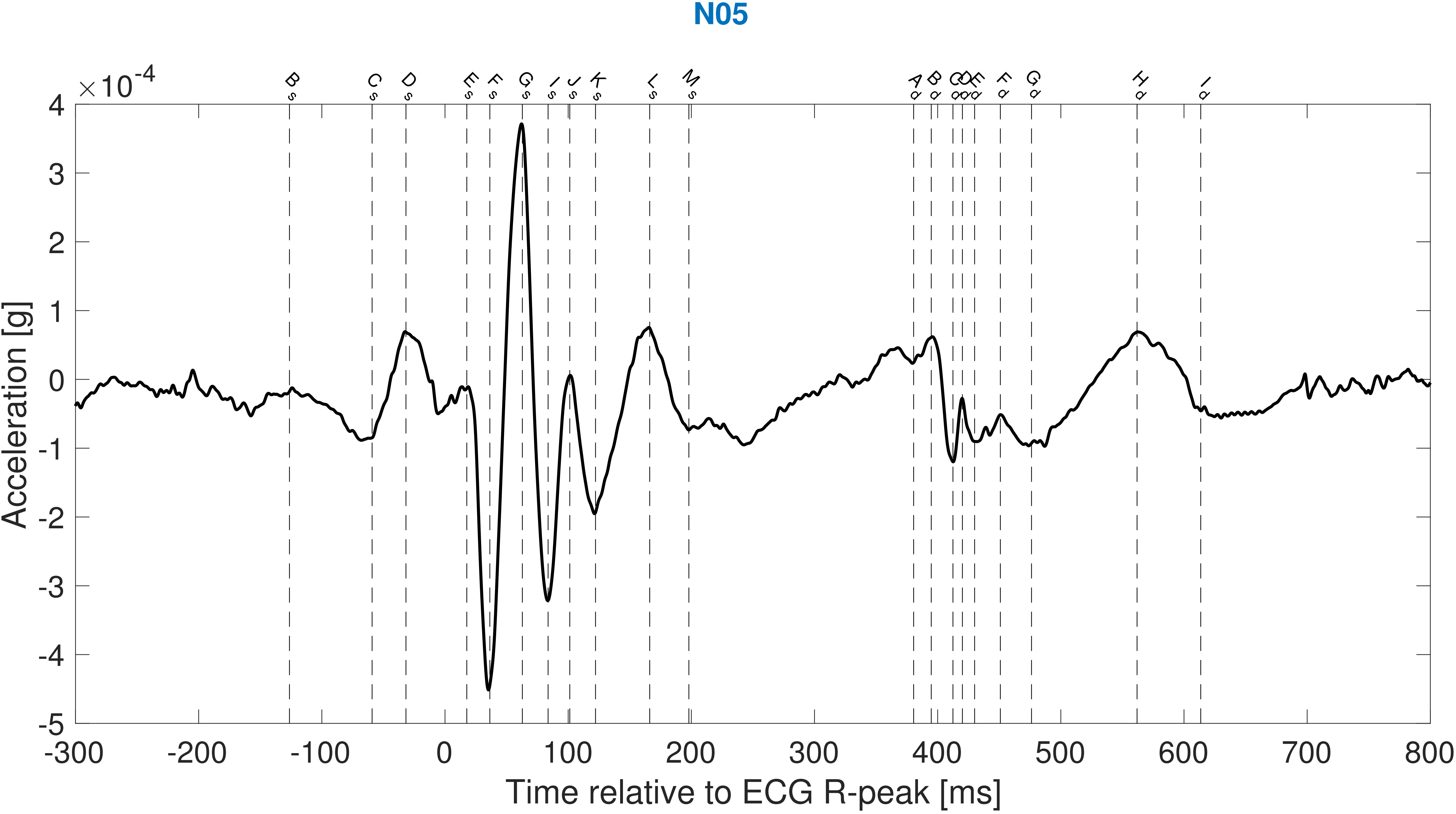

N05
×10^-4
B_s
C_s
D_s
E_s
F_s
G_s
I_s
J_s
K_s
L_s
M_s
A_d
B_d
H_d
I_d
F_d
G_d
4
3
2
1
0
-1
-2
-3
-4
-5
Acceleration [g]
-300
-200
-100
0
100
200
300
400
500
600
700
800
Time relative to ECG R-peak [ms]

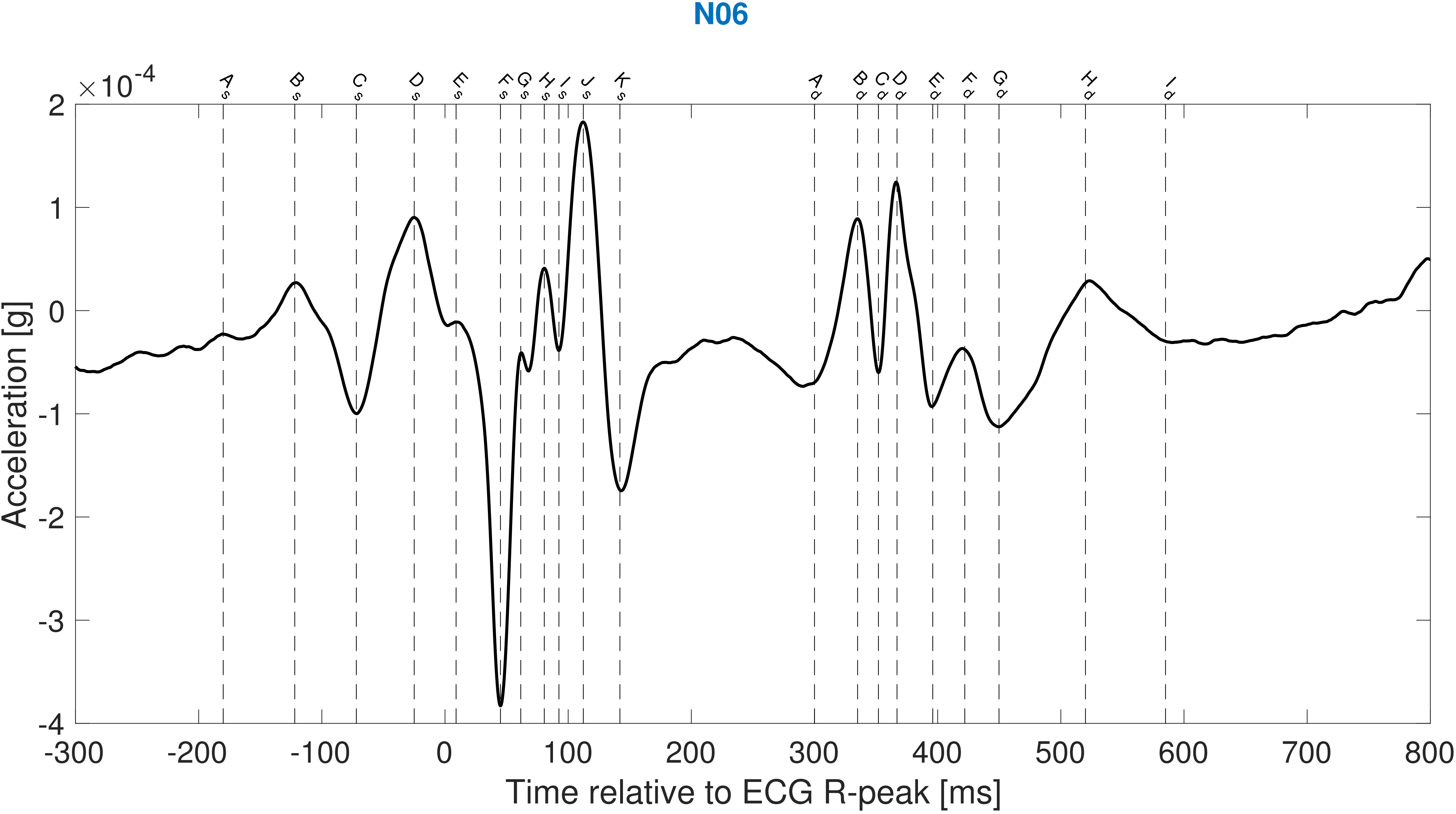

N06
×10^-4
Acceleration [g]
Time relative to ECG R-peak [ms]
A s
B s
C s
D s
E s
F s
G s
H s
I s
J s
K s
A d
B d
C d
D d
E d
F d
G d
H d
I d
2
1
0
-1
-2
-3
-4
-300
-200
-100
0
100
200
300
400
500
600
700
800

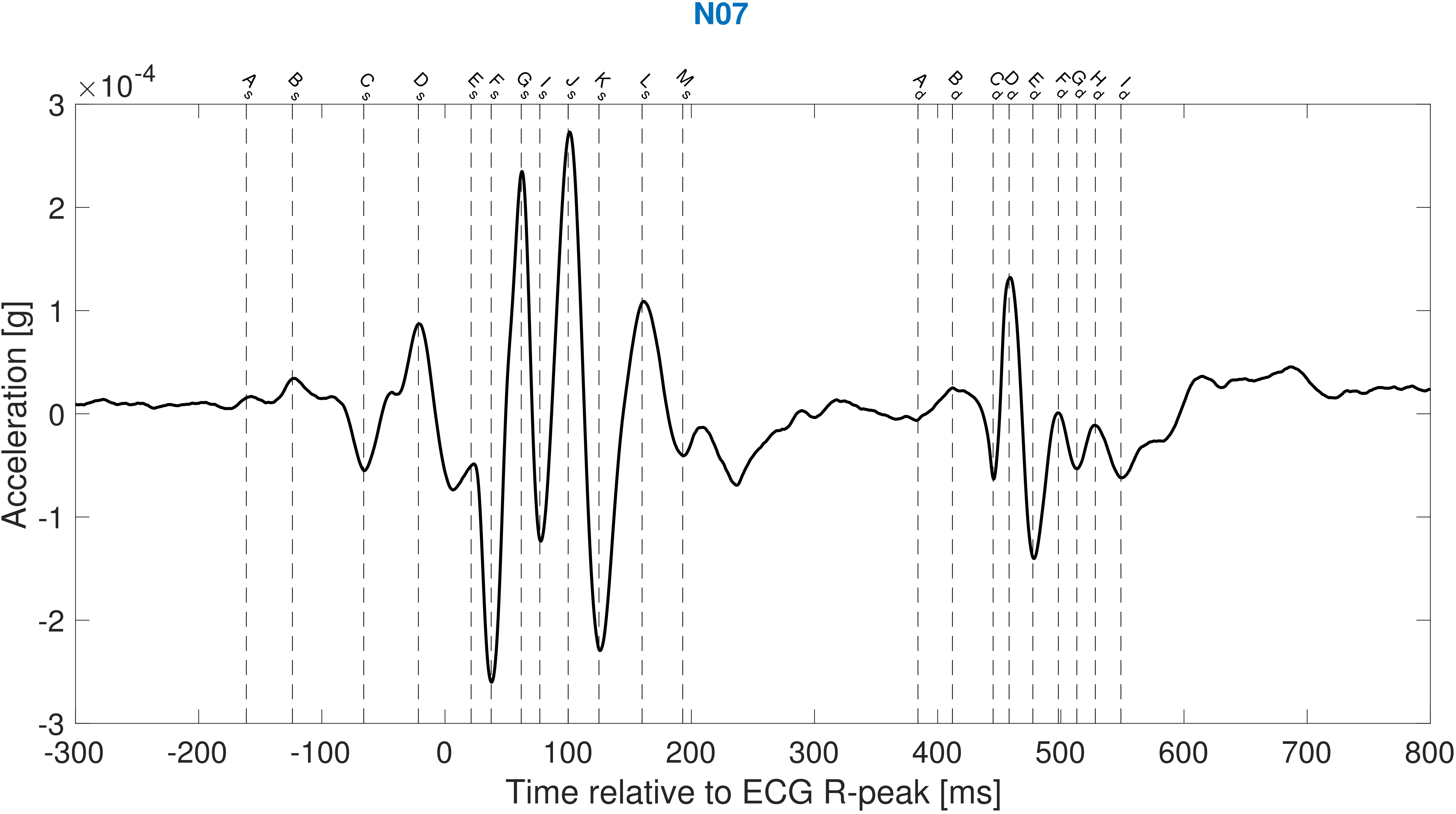
N07
×10^-4
Acceleration [g]
Time relative to ECG R-peak [ms]
As Bs Cs Ds Es Fs Gs Is Js Ks Ls Ms
Ad Bd Cd Dd Ed Fd Gd Hd Id
3 2 1 0 -1 -2 -3
-300 -200 -100 0 100 200 300 400 500 600 700 800

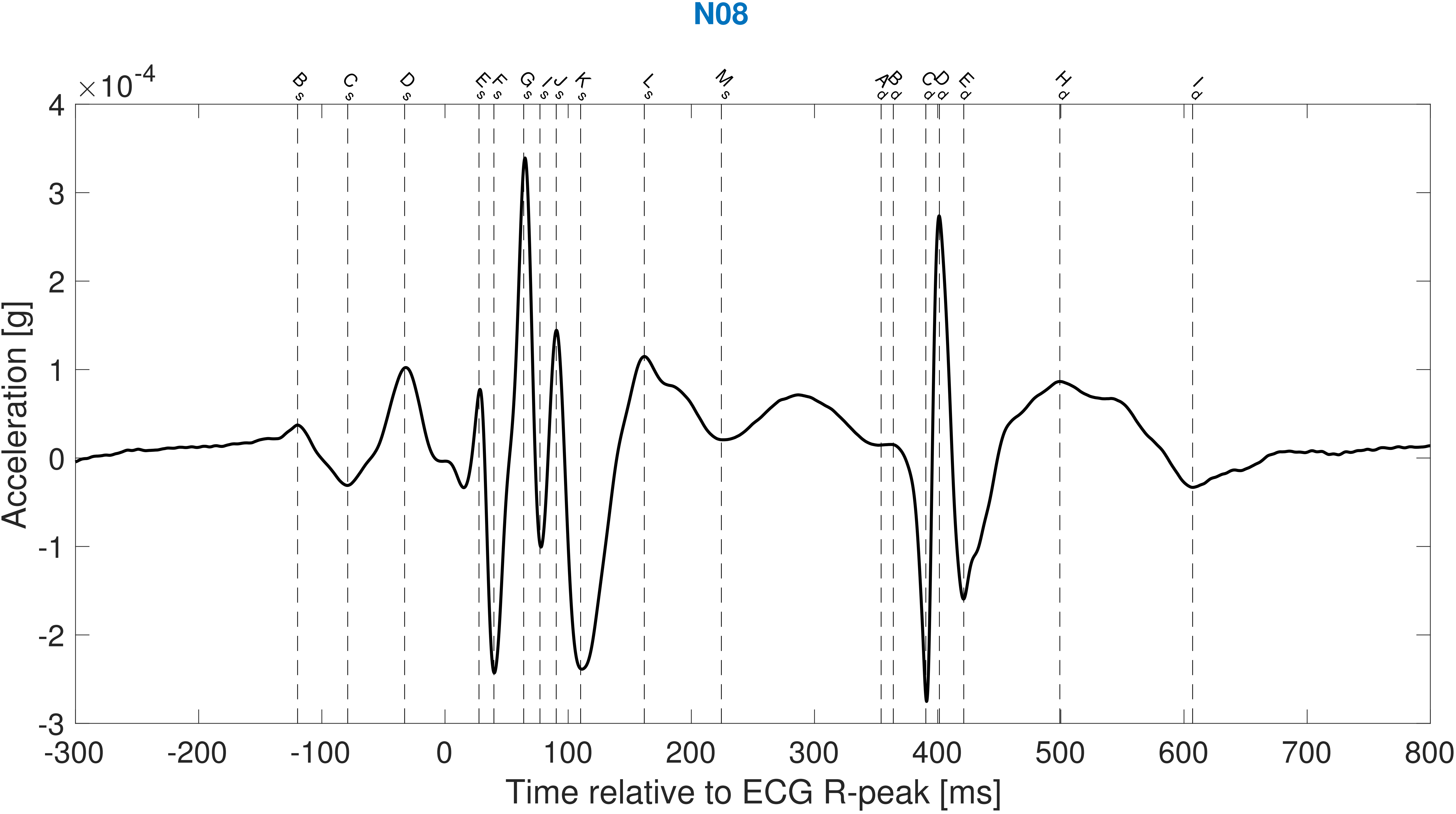

N08
×10^-4
4
3
2
1
0
-1
-2
-3
Acceleration [g]
-300
-200
-100
0
100
200
300
400
500
600
700
800
Time relative to ECG R-peak [ms]
B s
C s
D s
E s
F s
G s
I s
J s
K s
L s
M s
A d
B d
C d
D d
E d
H d
I d

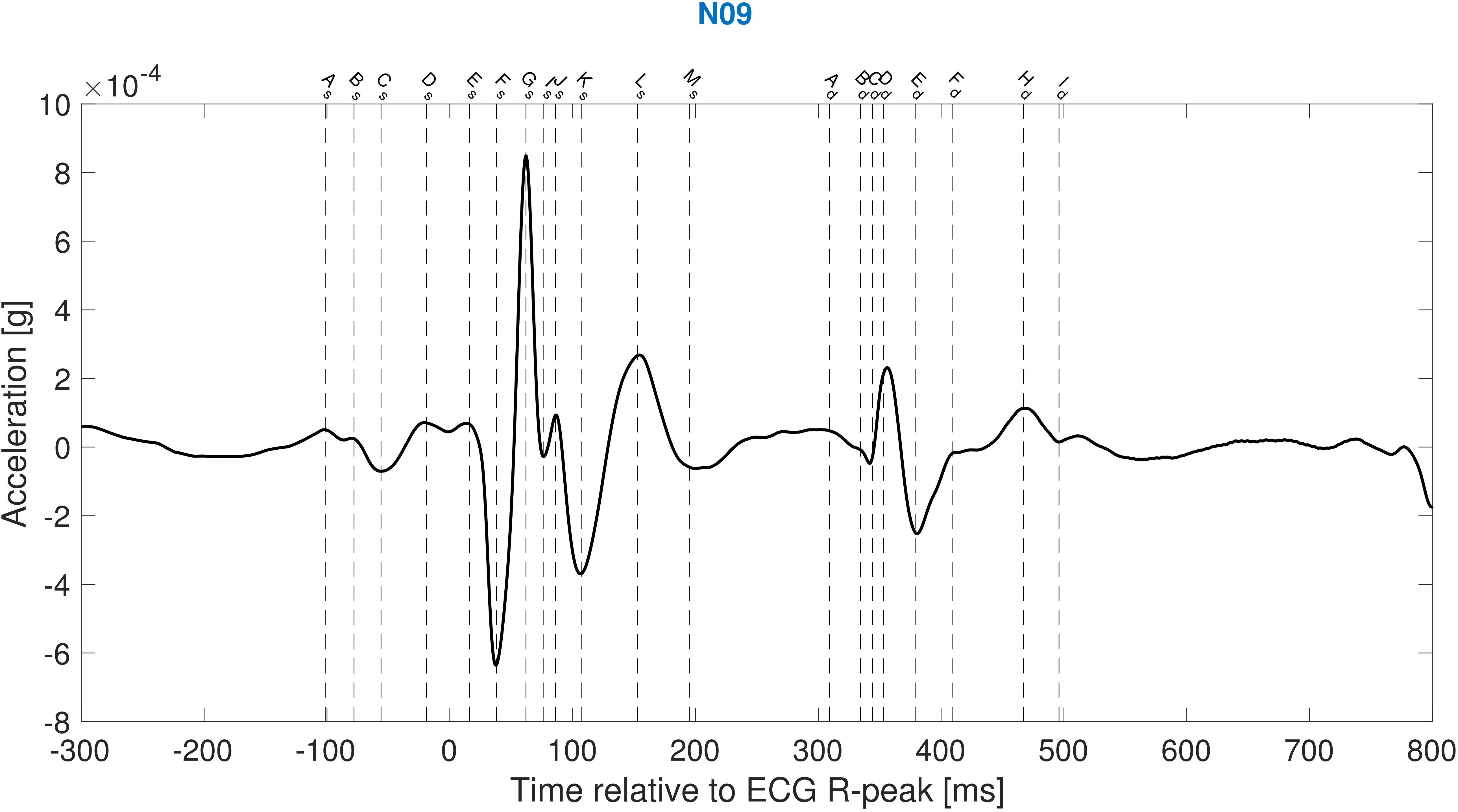

N09
×10^-4
Acceleration [g]
Time relative to ECG R-peak [ms]
A s
B s
C s
D s
E s
F s
G s
I s
J s
K s
L s
M s
A d
B d
C d
D d
E d
F d
H d
I d

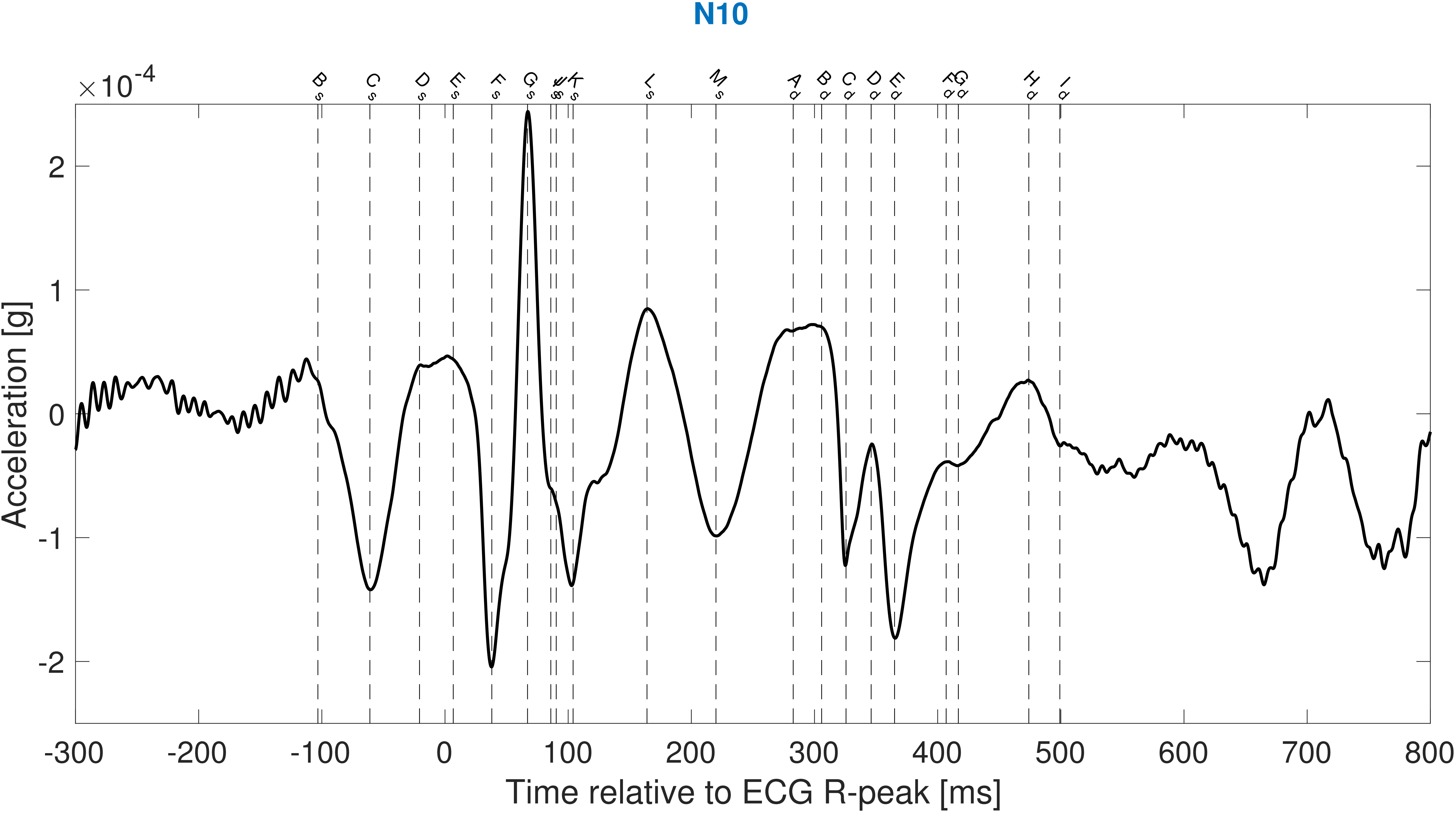
N10
×10^-4
Acceleration [g]
Time relative to ECG R-peak [ms]
Bs
Cs
Ds
Es
Fs
Gs
Ks
Ls
Ms
Ad
Bd
Cd
Dd
Ed
Fd
Gd
Hd
Id
2
1
0
-1
-2
-300
-200
-100
0
100
200
300
400
500
600
700
800

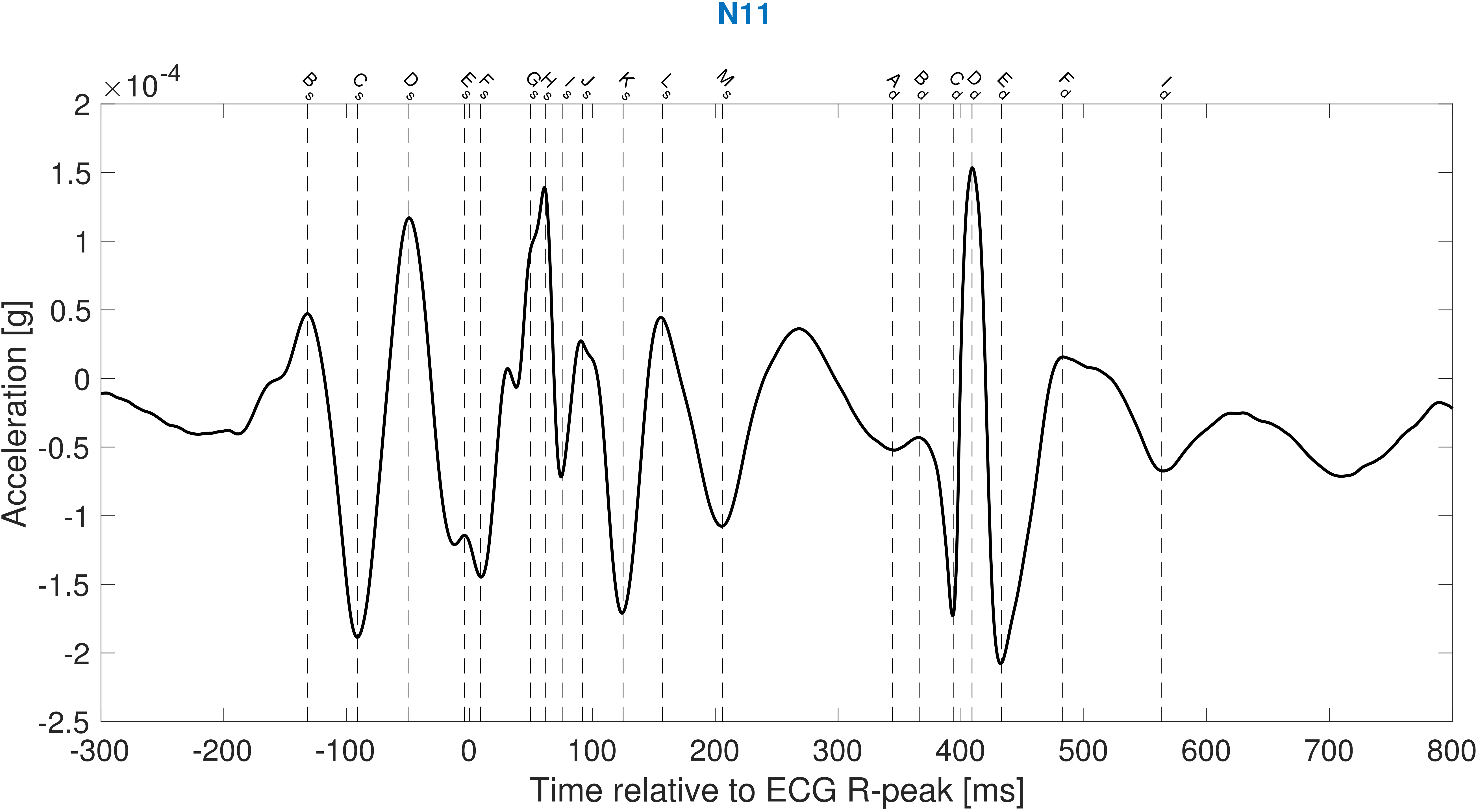
N11
×10^-4
Acceleration [g]
Time relative to ECG R-peak [ms]
B s
C s
D s
E s
F s
G s
H s
I s
J s
K s
L s
M s
A d
B d
C d
D d
E d
F d
I d
2
1.5
1
0.5
0
-0.5
-1
-1.5
-2
-2.5
-300
-200
-100
0
100
200
300
400
500
600
700
800

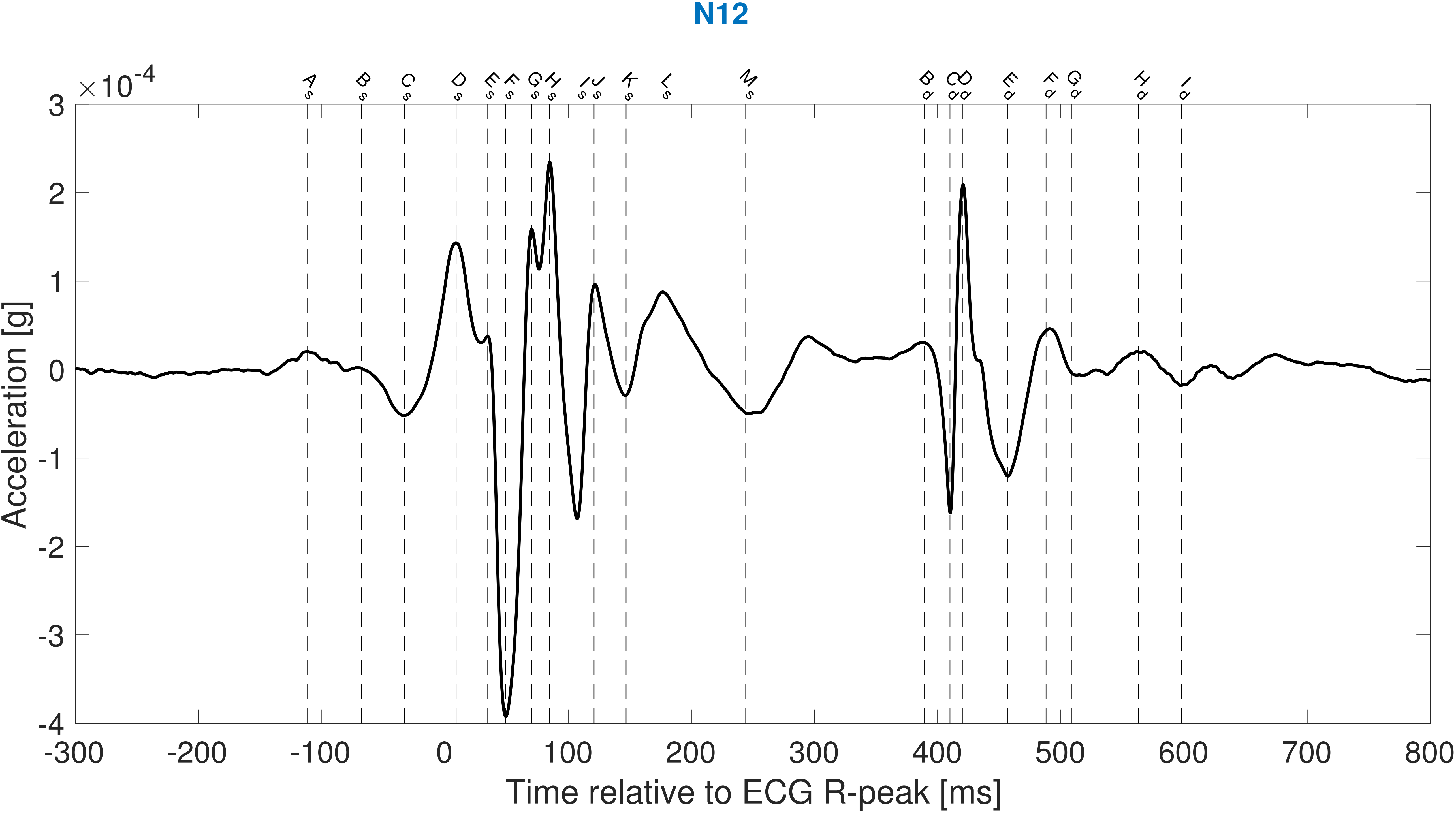

N12
$\times 10^{-4}$
A_s
B_s
C_s
D_s
E_s
F_s
G_s
H_s
I_s
J_s
K_s
L_s
M_s
B_d
C_d
D_d
E_d
F_d
G_d
H_d
I_d
3
2
1
0
-1
-2
-3
-4
Acceleration [g]
-300
-200
-100
0
100
200
300
400
500
600
700
800
Time relative to ECG R-peak [ms]

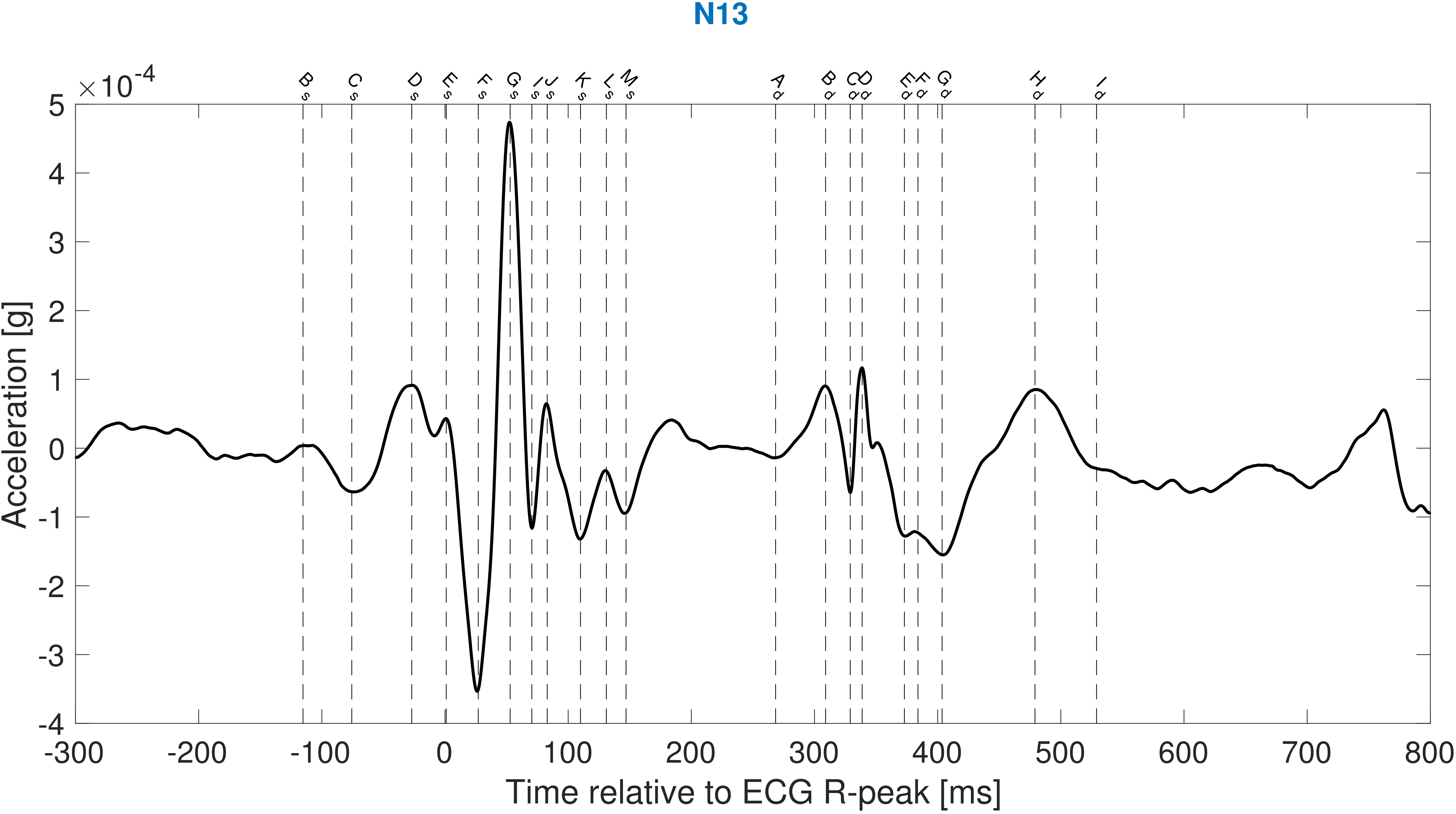

N13
×10^-4
Acceleration [g]
Time relative to ECG R-peak [ms]
B s
C s
D s
E s
F s
G s
I s
J s
K s
L s
M s
A d
B d
C d
D d
E d
F d
G d
H d
I d

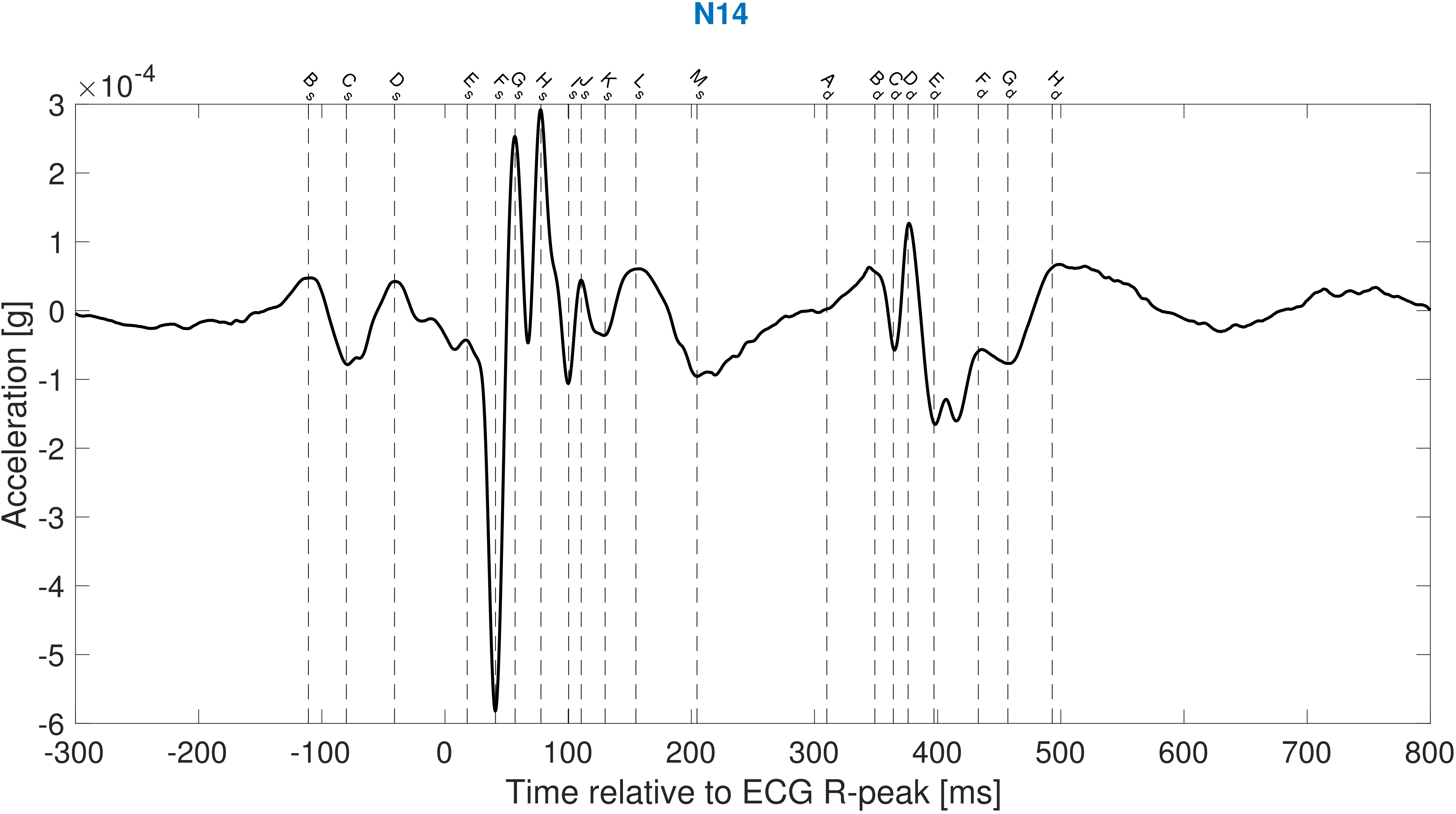
N14
×10^-4
Acceleration [g]
Time relative to ECG R-peak [ms]
B s
C s
D s
E s
F s
G s
H s
I s
J s
K s
L s
M s
A d
B d
C d
D d
E d
F d
G d
H d
3
2
1
0
-1
-2
-3
-4
-5
-6
-300
-200
-100
0
100
200
300
400
500
600
700
800

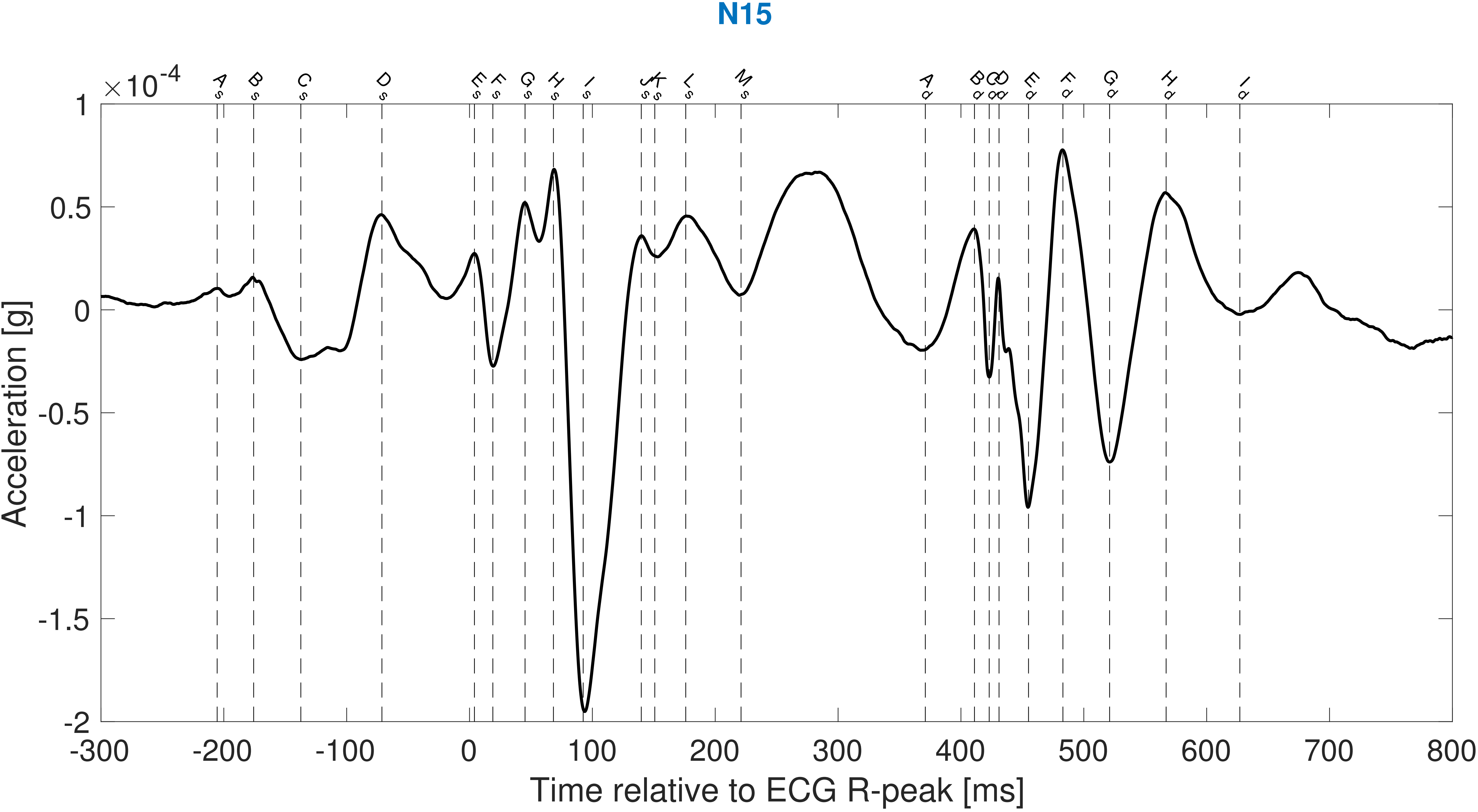
N15
$\times 10^{-4}$
Acceleration [g]
Time relative to ECG R-peak [ms]
1
0.5
0
-0.5
-1
-1.5
-2
-300
-200
-100
0
100
200
300
400
500
600
700
800
A_s B_s C_s D_s E_s F_s G_s H_s I_s J_s K_s L_s M_s
A_d B_d C_d D_d E_d F_d G_d H_d I_d

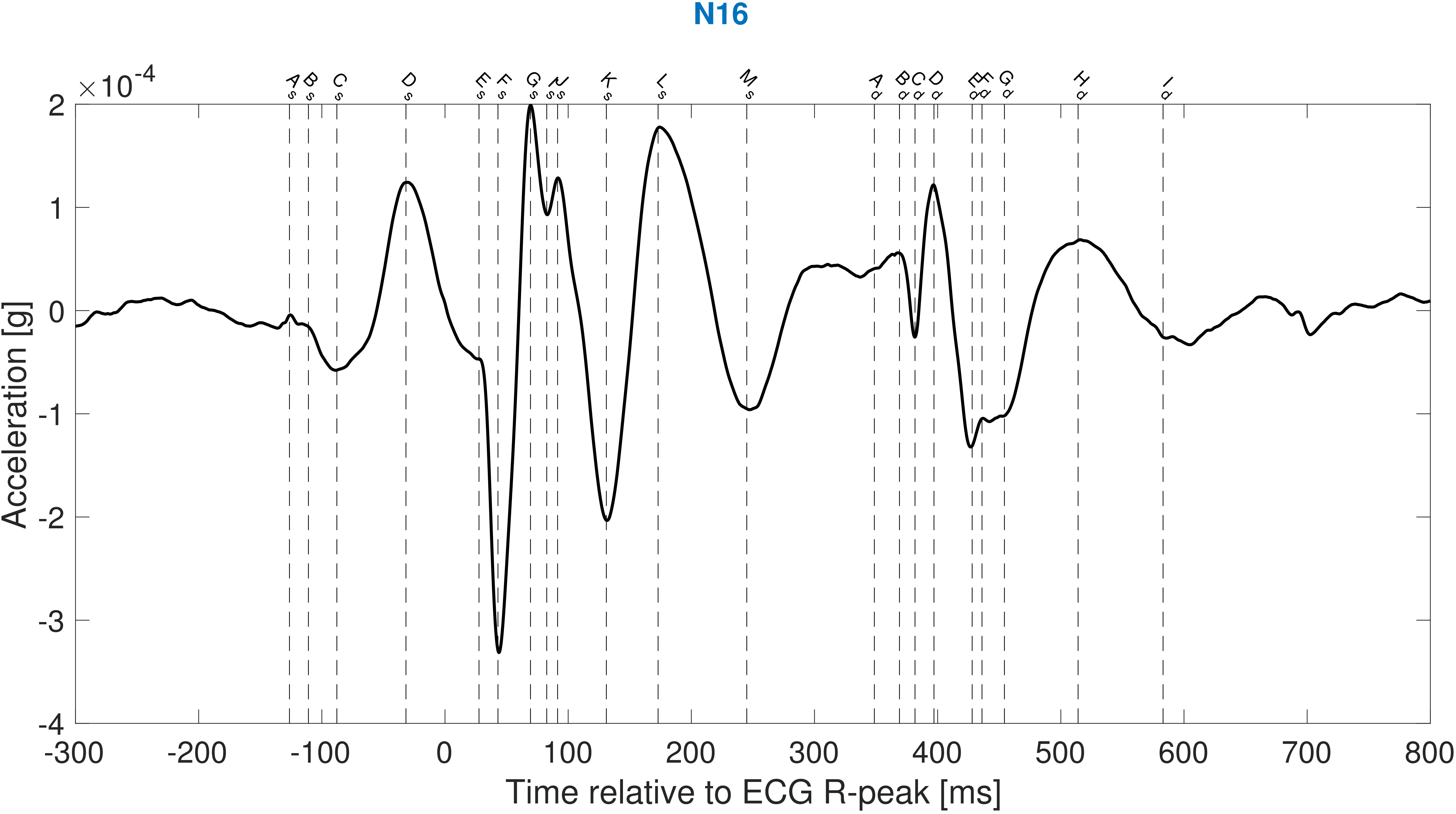

N16
×10^-4
Acceleration [g]
Time relative to ECG R-peak [ms]
2
1
0
-1
-2
-3
-4
-300
-200
-100
0
100
200
300
400
500
600
700
800
A s
B s
C s
D s
E s
F s
G s
I s
J s
K s
L s
M s
A d
B d
C d
D d
E d
F d
G d
H d
I d

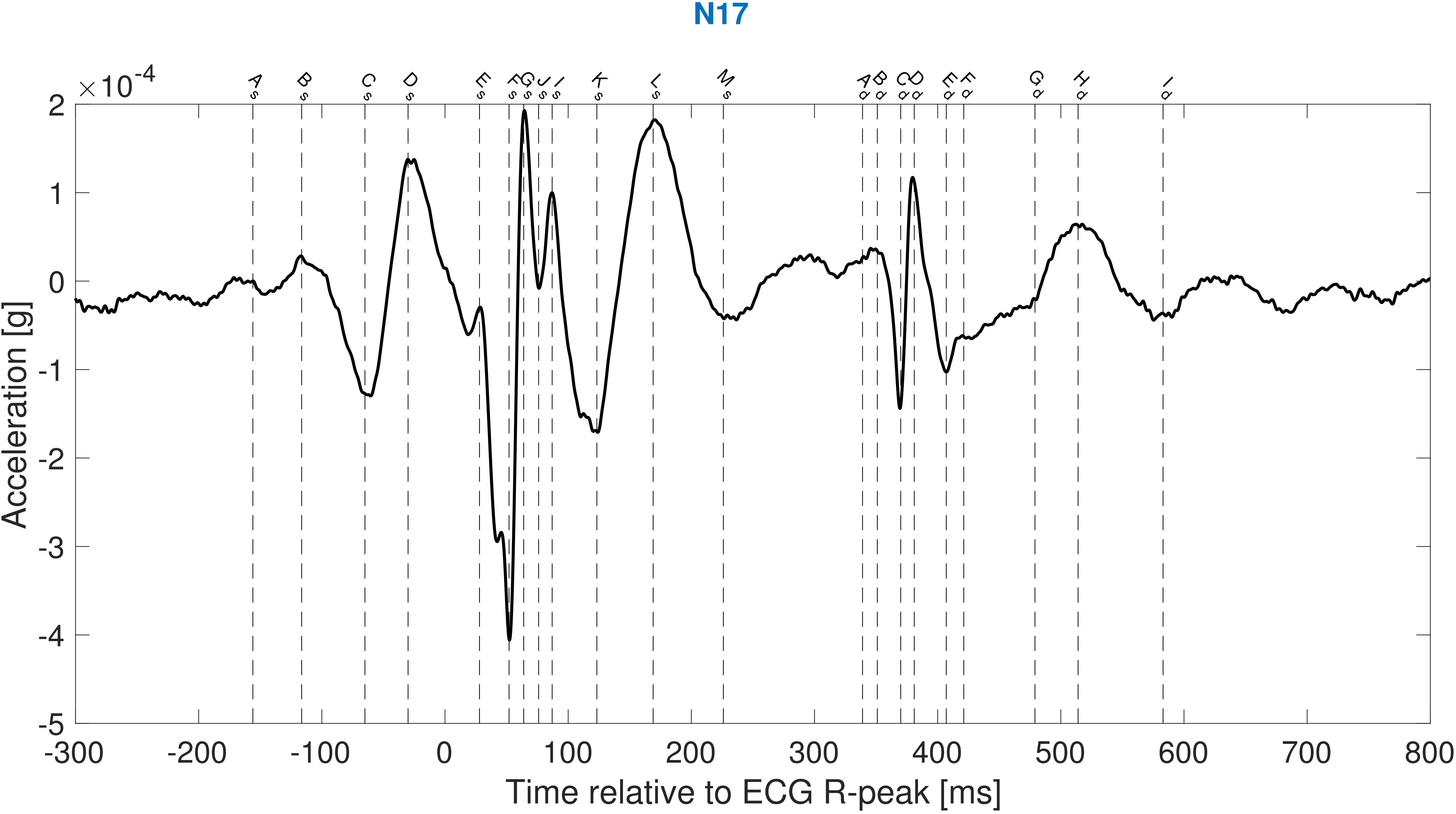

N17
×10^-4
2
1
0
-1
-2
-3
-4
-5
Acceleration [g]
-300
-200
-100
0
100
200
300
400
500
600
700
800
Time relative to ECG R-peak [ms]
A s
B s
C s
D s
E s
F s
G s
J s
I s
K s
L s
M s
A d
B d
C d
D d
E d
F d
G d
H d
I d

N19

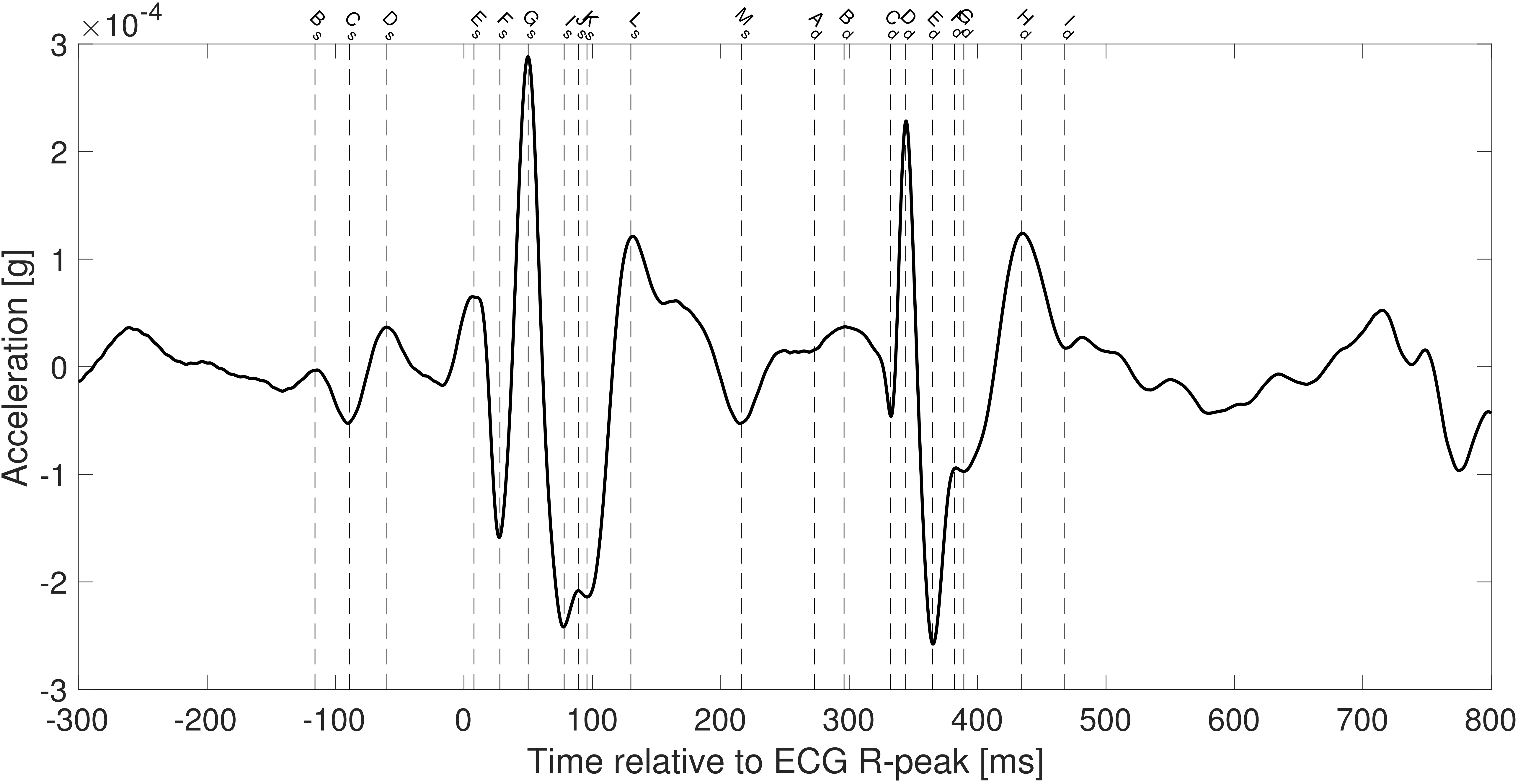

$\times 10^{-4}$
B s
C s
D s
E s
F s
G s
I s
J s
K s
L s
M s
A d
B d
C d
D d
E d
F d
G d
H d
I d
3
2
1
0
-1
-2
-3
Acceleration [g]
-300
-200
-100
0
100
200
300
400
500
600
700
800
Time relative to ECG R-peak [ms]

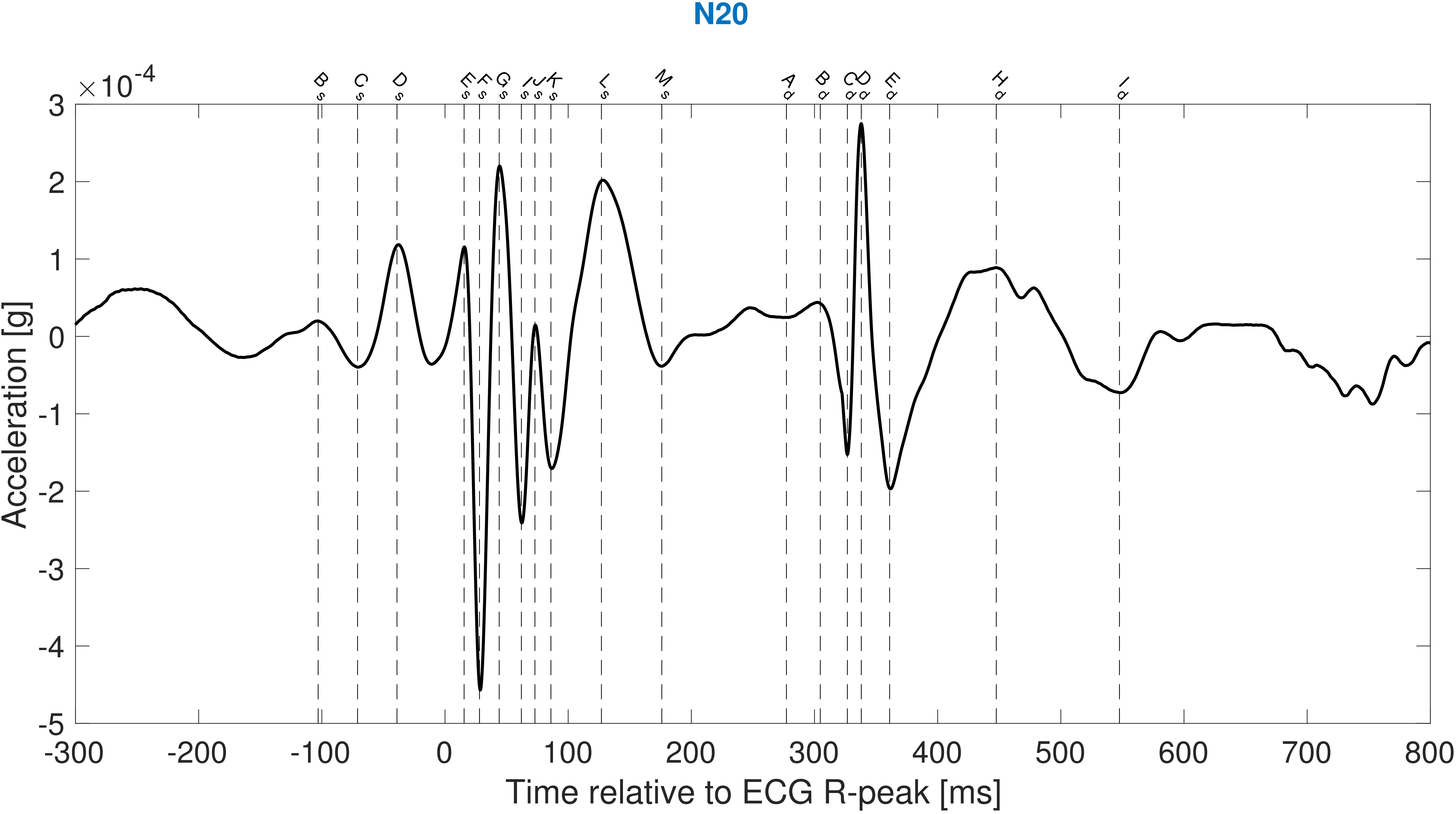

N20
×10^-4
Acceleration [g]
Time relative to ECG R-peak [ms]
B s
C s
D s
E s
F s
G s
I s
J s
K s
L s
M s
A d
B d
C d
D d
E d
H d
I d
3
2
1
0
-1
-2
-3
-4
-5
-300
-200
-100
0
100
200
300
400
500
600
700
800

N21

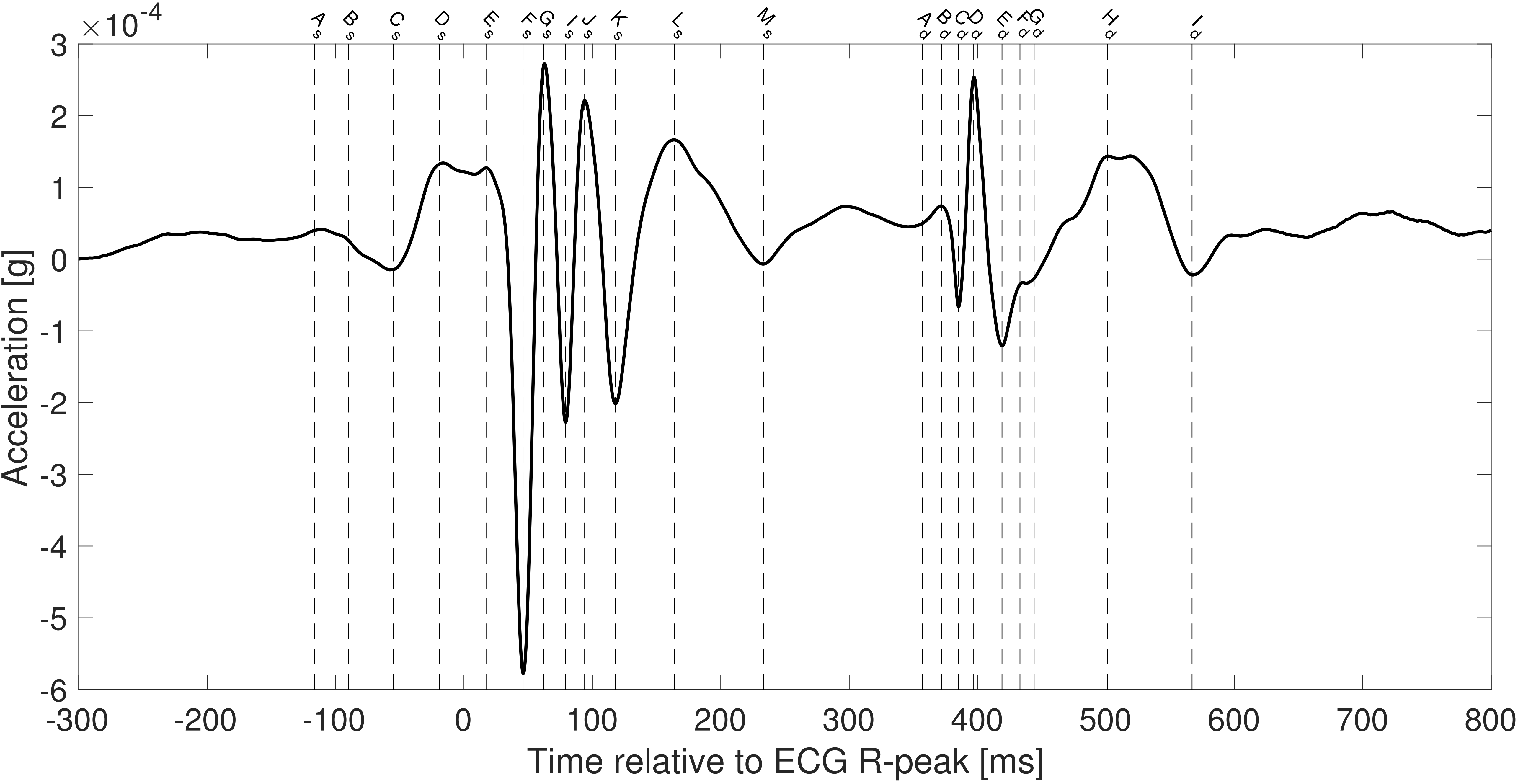

×10^-4
A_s B_s C_s D_s E_s F_s G_s I_s J_s K_s L_s M_s
A_d B_d C_d D_d E_d F_d G_d H_d I_d
3
2
1
0
-1
-2
-3
-4
-5
-6
Acceleration [g]
-300 -200 -100 0 100 200 300 400 500 600 700 800
Time relative to ECG R-peak [ms]

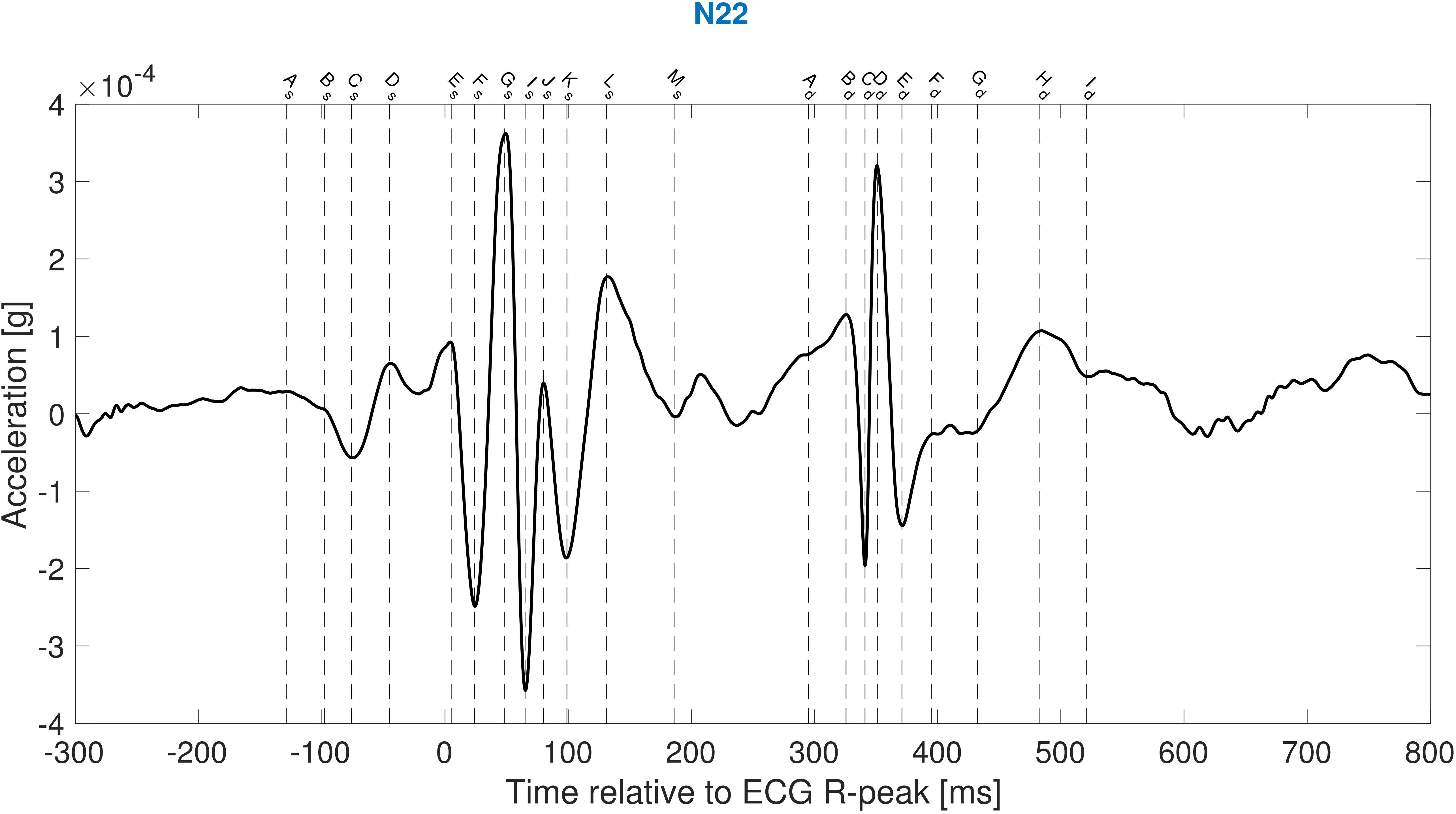
N22
$\times 10^{-4}$
Acceleration [g]
Time relative to ECG R-peak [ms]
A_s B_s C_s D_s E_s F_s G_s I_s J_s K_s L_s M_s
A_d B_d C_d D_d E_d F_d G_d H_d I_d
4 3 2 1 0 -1 -2 -3 -4
-300 -200 -100 0 100 200 300 400 500 600 700 800

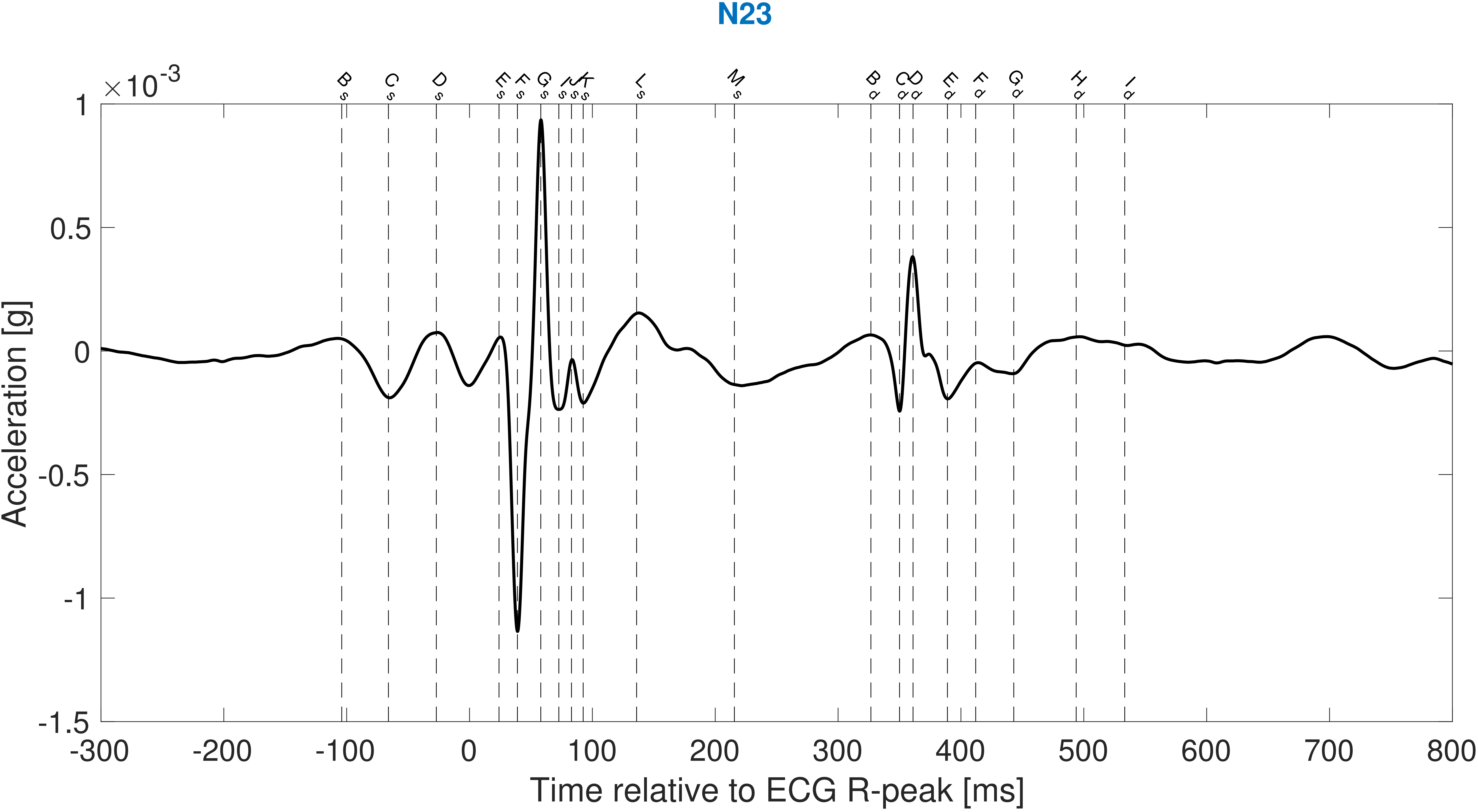

N23
×10^-3
1
0.5
0
-0.5
-1
-1.5
Acceleration [g]
-300
-200
-100
0
100
200
300
400
500
600
700
800
Time relative to ECG R-peak [ms]
Bs
Cs
Ds
Es
Fs
Gs
Is
Js
Ks
Ls
Ms
Bd
Cd
Dd
Ed
Fd
Gd
Hd
Id

N24

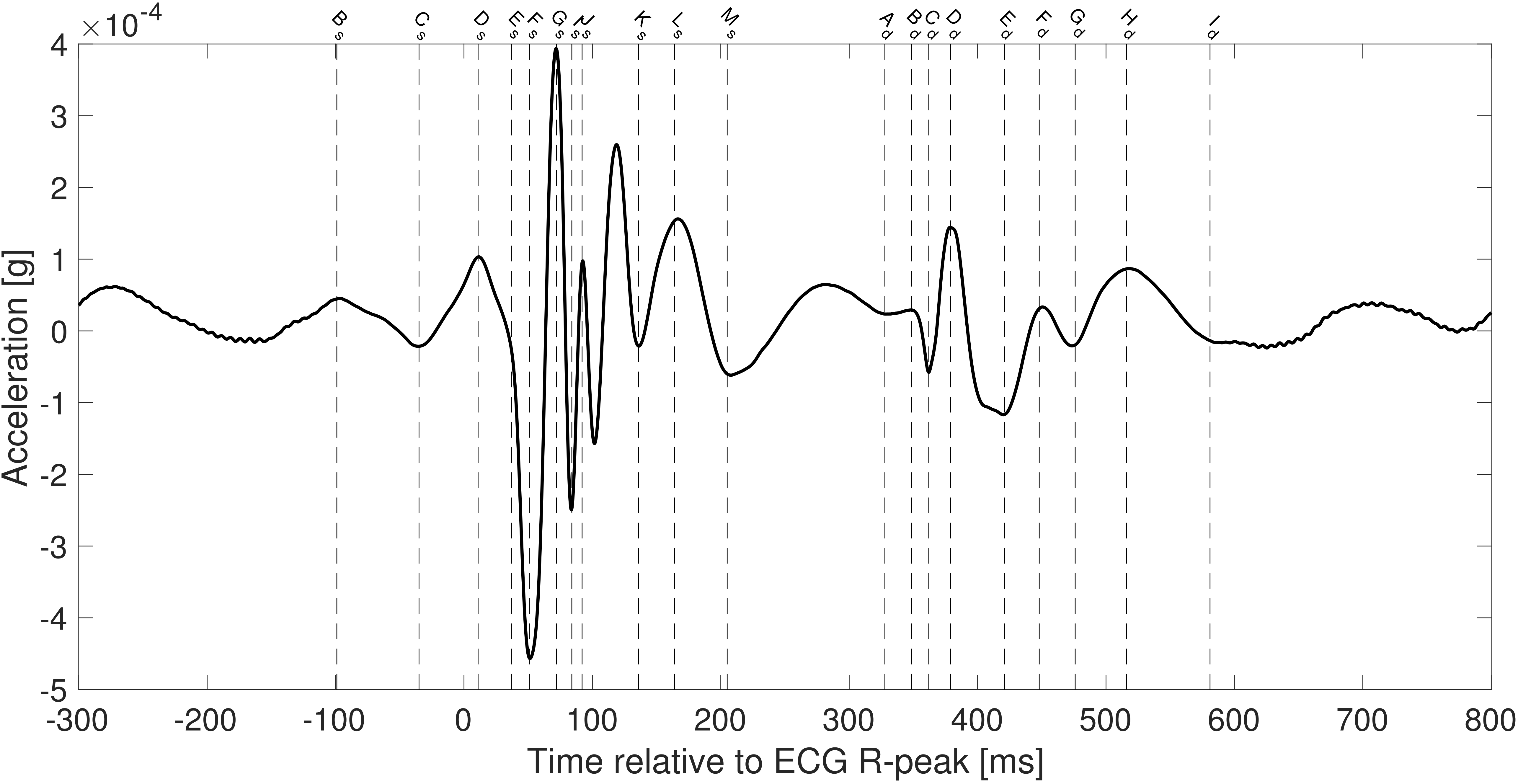

×10^-4
Bs
Cs
Ds
Es
Fs
Gs
Ks
Ls
Ms
Ad
Bd
Cd
Dd
Ed
Fd
Gd
Hd
Id
Acceleration [g]
4
3
2
1
0
-1
-2
-3
-4
-5
-300
-200
-100
0
100
200
300
400
500
600
700
800
Time relative to ECG R-peak [ms]

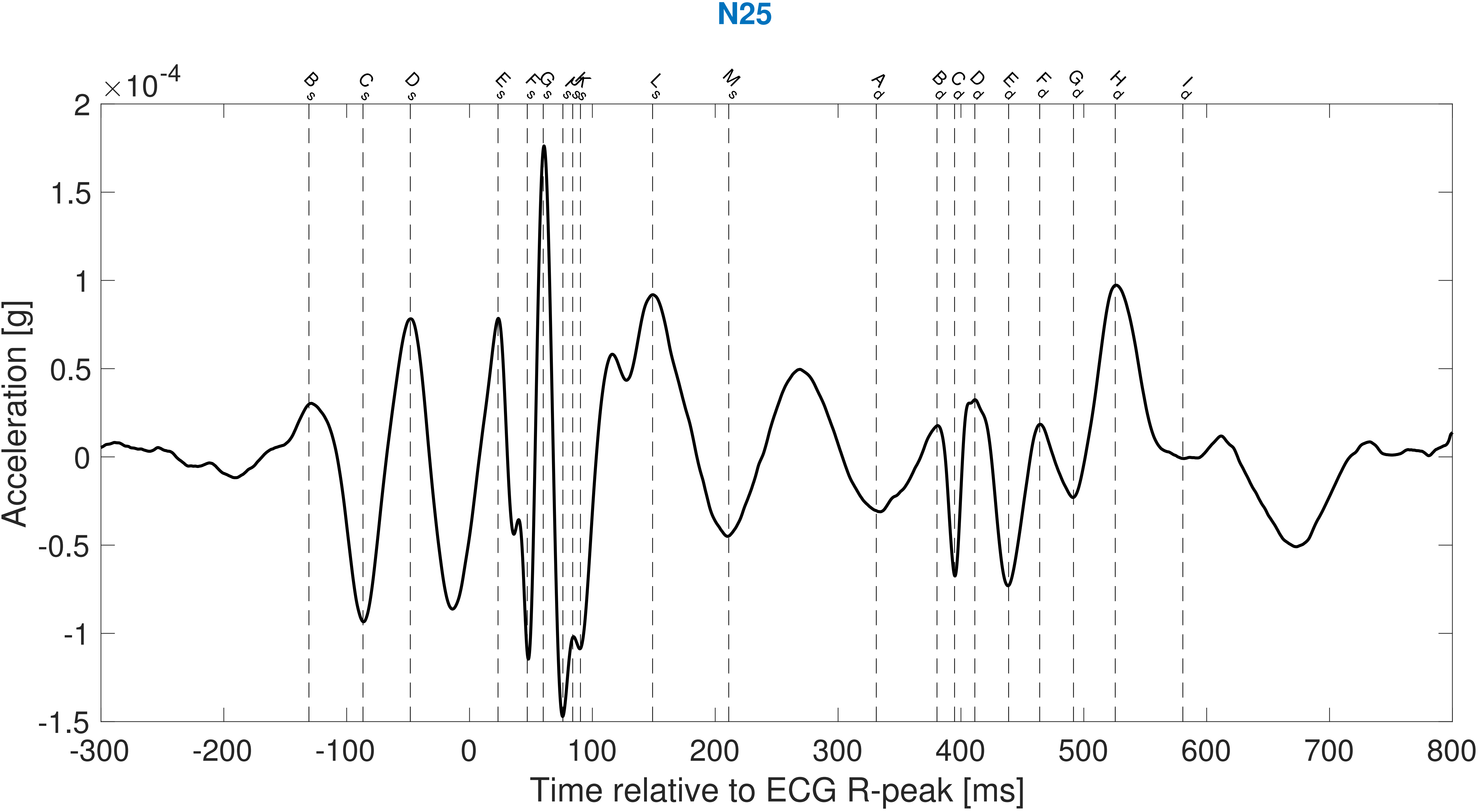

N25
×10^-4
Acceleration [g]
Time relative to ECG R-peak [ms]
-300
-200
-100
0
100
200
300
400
500
600
700
800
2
1.5
1
0.5
0
-0.5
-1
-1.5

N26

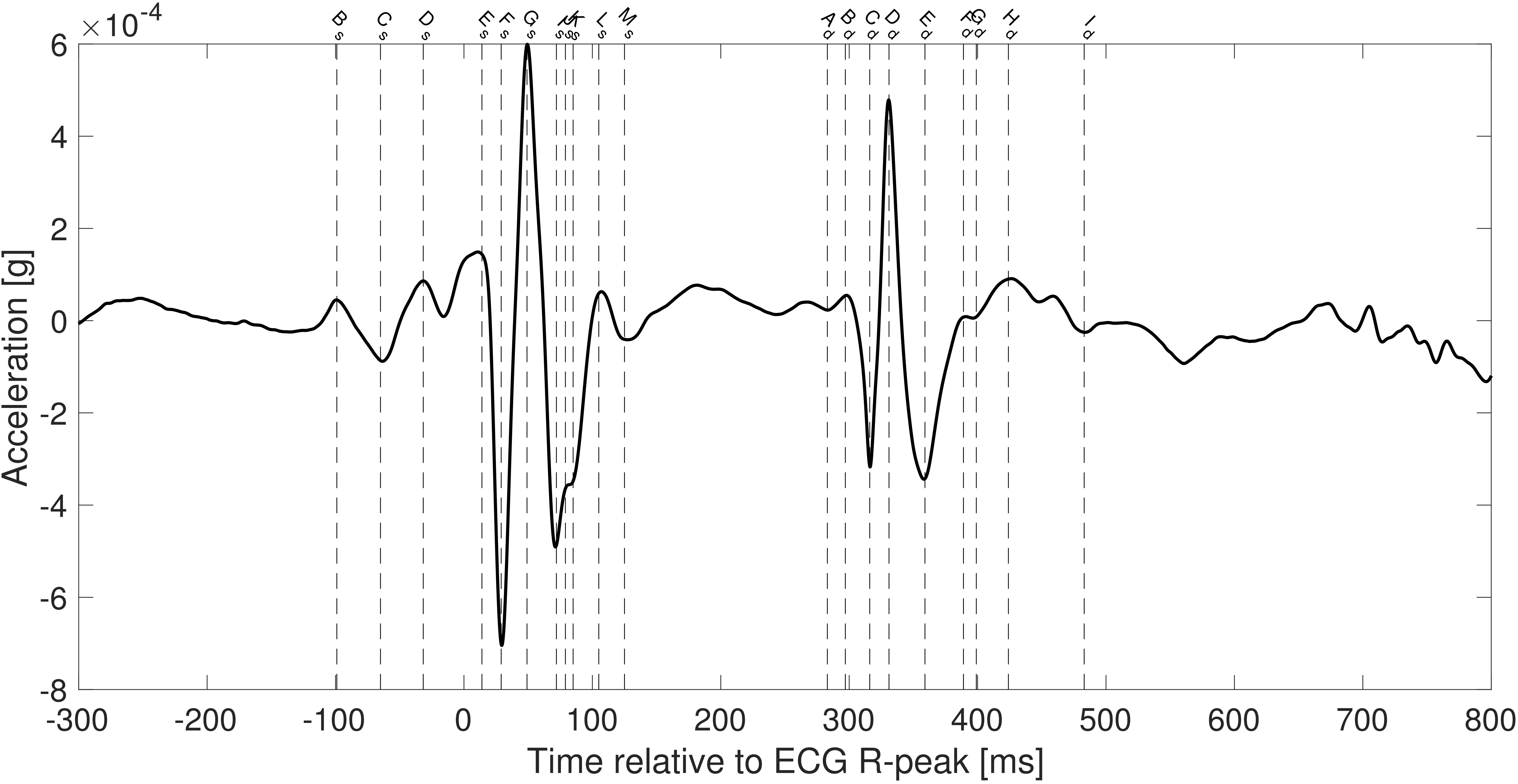

×10^-4
Acceleration [g]
Time relative to ECG R-peak [ms]
B s
C s
D s
E s
F s
G s
L s
M s
A d
B d
C d
D d
E d
H d
I d

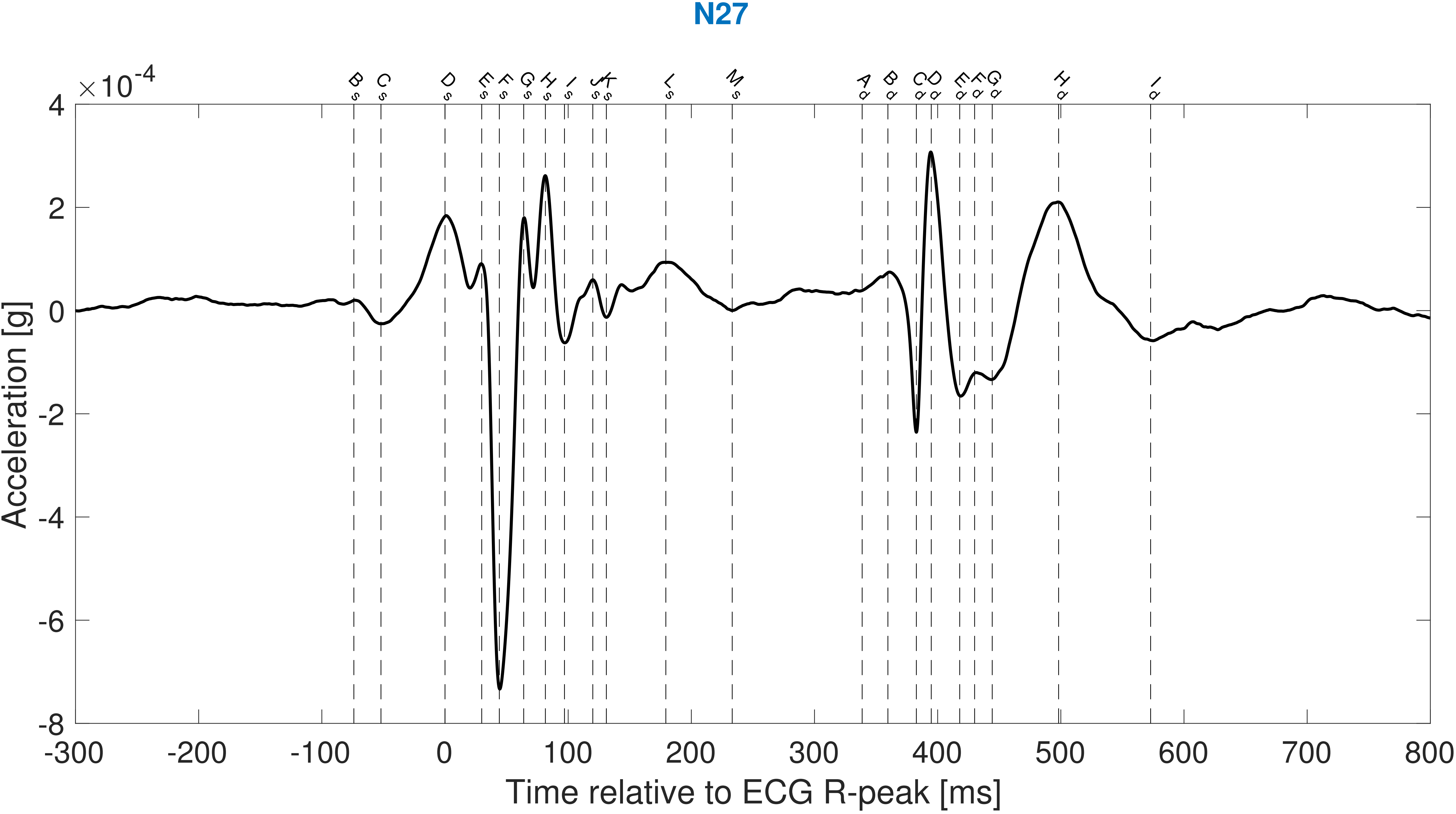

N27
B_s
C_s
D_s
E_s
F_s
G_s
H_s
I_s
J_s
K_s
L_s
M_s
A_d
B_d
C_d
D_d
E_d
F_d
G_d
H_d
I_d
$\times 10^{-4}$
4
2
0
-2
-4
-6
-8
Acceleration [g]
-300
-200
-100
0
100
200
300
400
500
600
700
800
Time relative to ECG R-peak [ms]

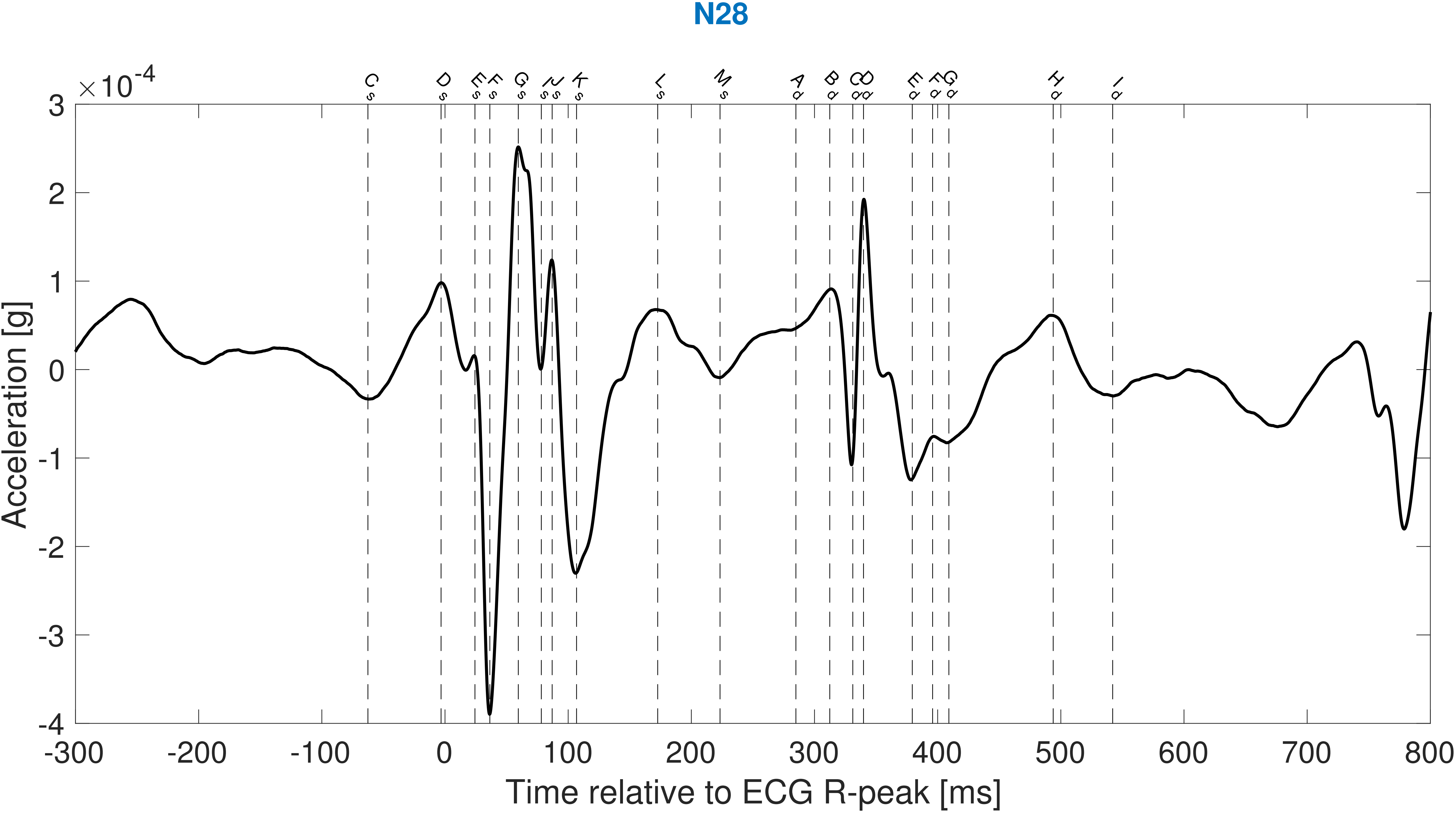

N28
$\times 10^{-4}$
Acceleration [g]
Time relative to ECG R-peak [ms]
Cs
Ds
Es
Fs
Gs
Is
Js
Ks
Ls
Ms
Ad
Bd
Cd
Dd
Ed
Fd
Gd
Hd
Id
3
2
1
0
-1
-2
-3
-4
-300
-200
-100
0
100
200
300
400
500
600
700
800

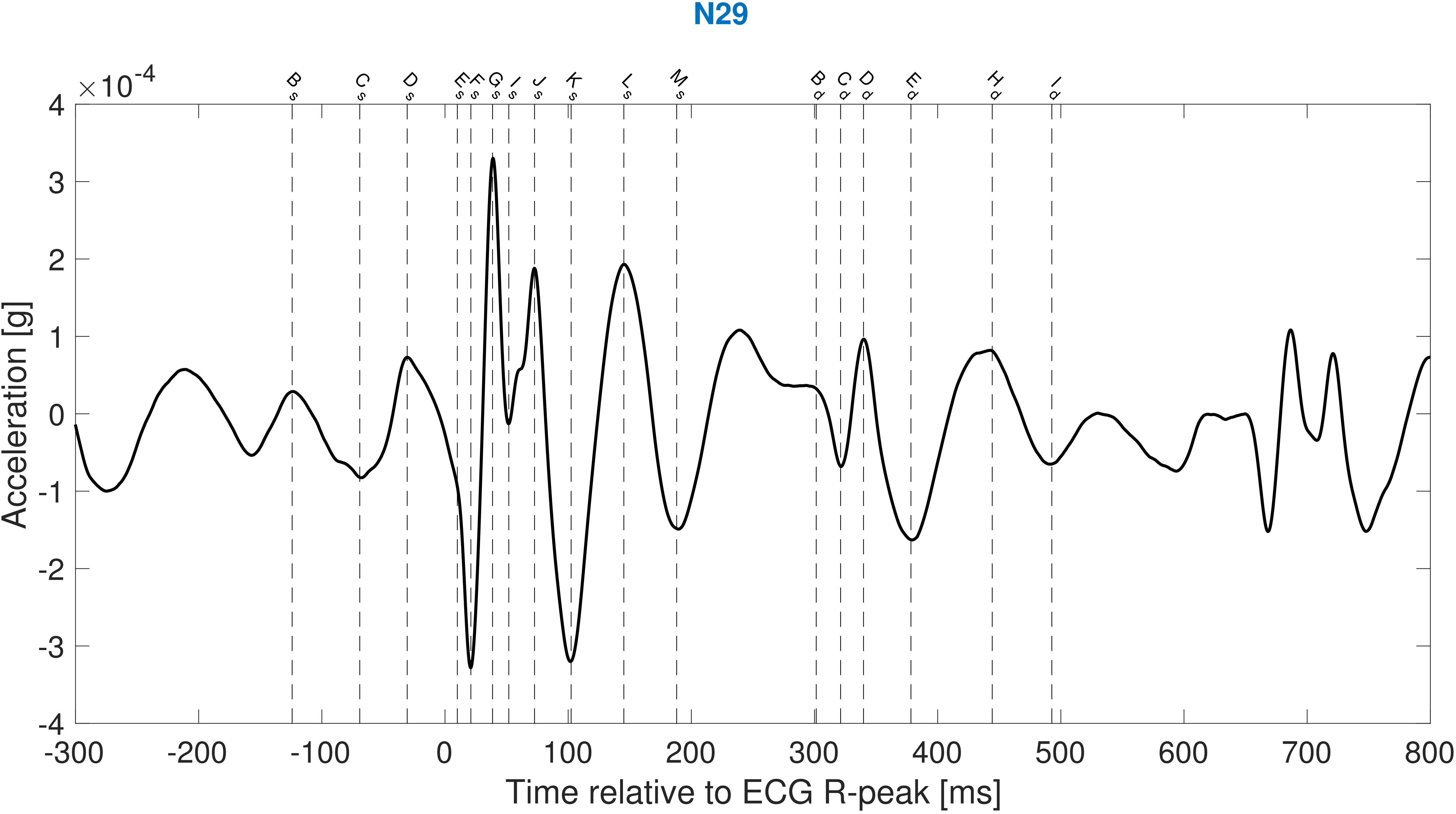

N29
B s
C s
D s
E s
F s
G s
I s
J s
K s
L s
M s
B d
C d
D d
E d
H d
I d
× 10^-4
4
3
2
1
0
-1
-2
-3
-4
Acceleration [g]
-300
-200
-100
0
100
200
300
400
500
600
700
800
Time relative to ECG R-peak [ms]

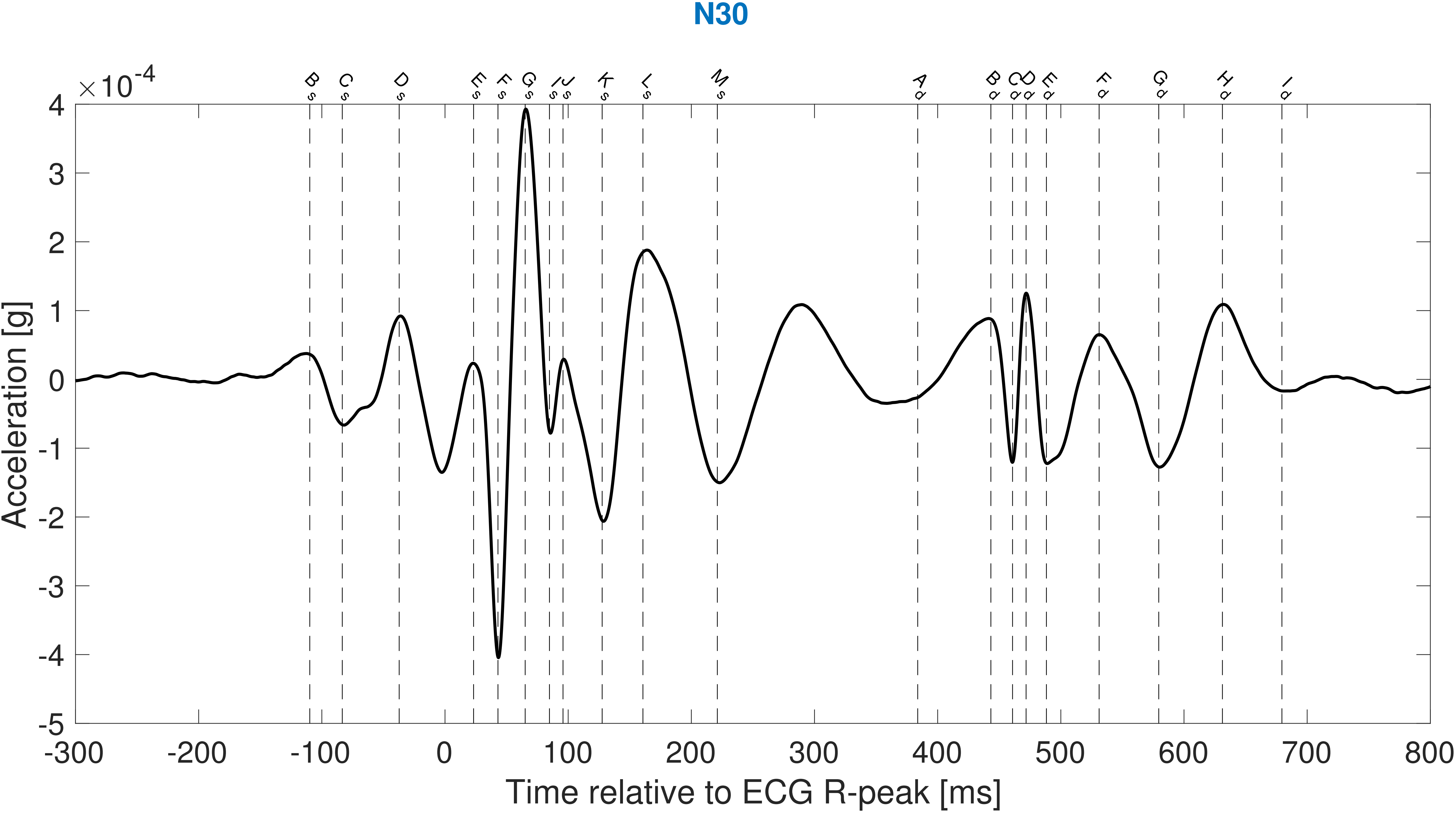

N30
$\times 10^{-4}$
Acceleration [g]
Time relative to ECG R-peak [ms]
4
3
2
1
0
-1
-2
-3
-4
-5
-300
-200
-100
0
100
200
300
400
500
600
700
800
B_s
C_s
D_s
E_s
F_s
G_s
I_s
J_s
K_s
L_s
M_s
A_d
B_d
C_d
D_d
E_d
F_d
G_d
H_d
I_d

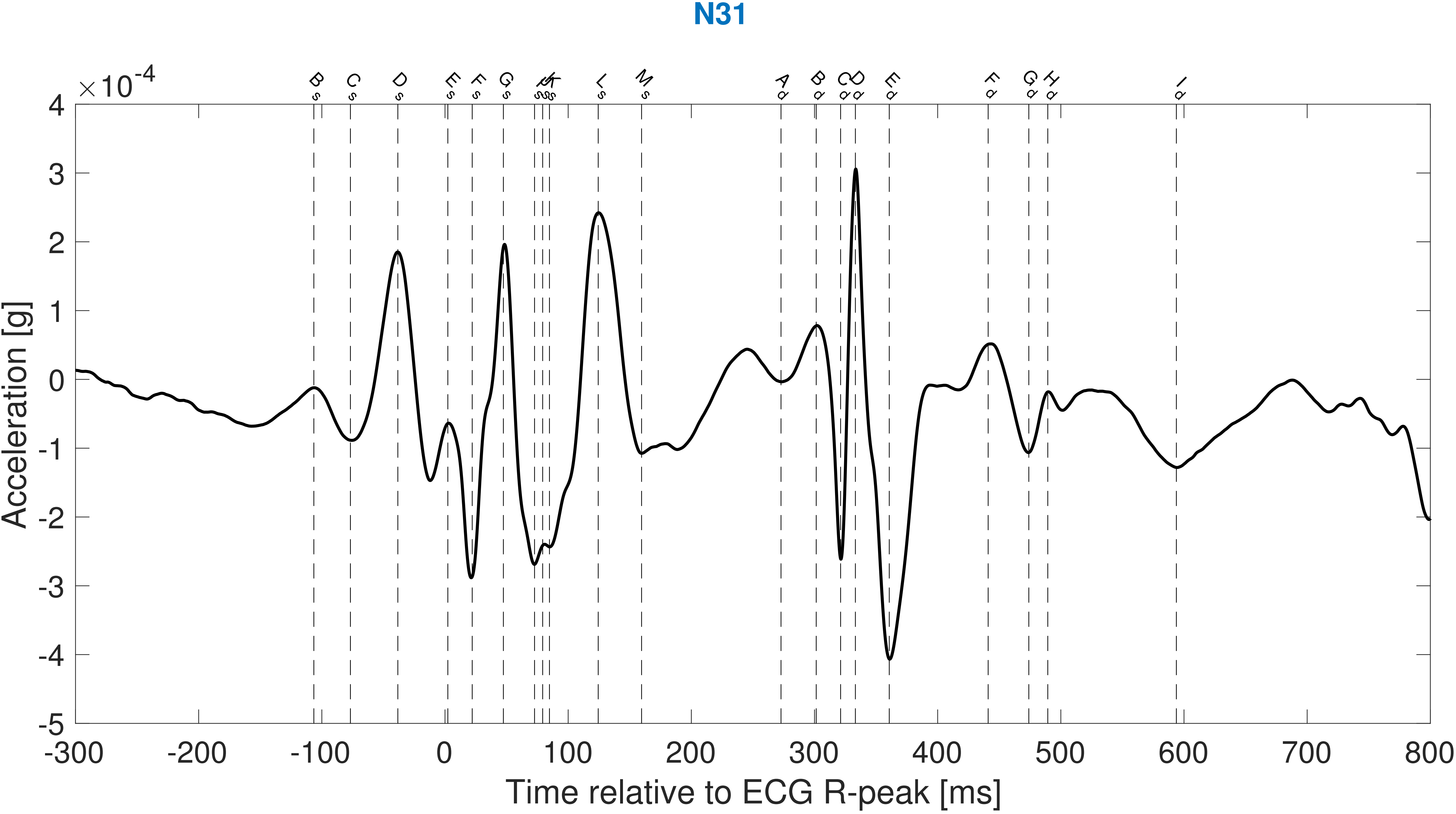
N31
×10^-4
Acceleration [g]
Time relative to ECG R-peak [ms]
B s
C s
D s
E s
F s
G s
K s
L s
M s
A d
B d
C d
D d
E d
F d
G d
H d
I d

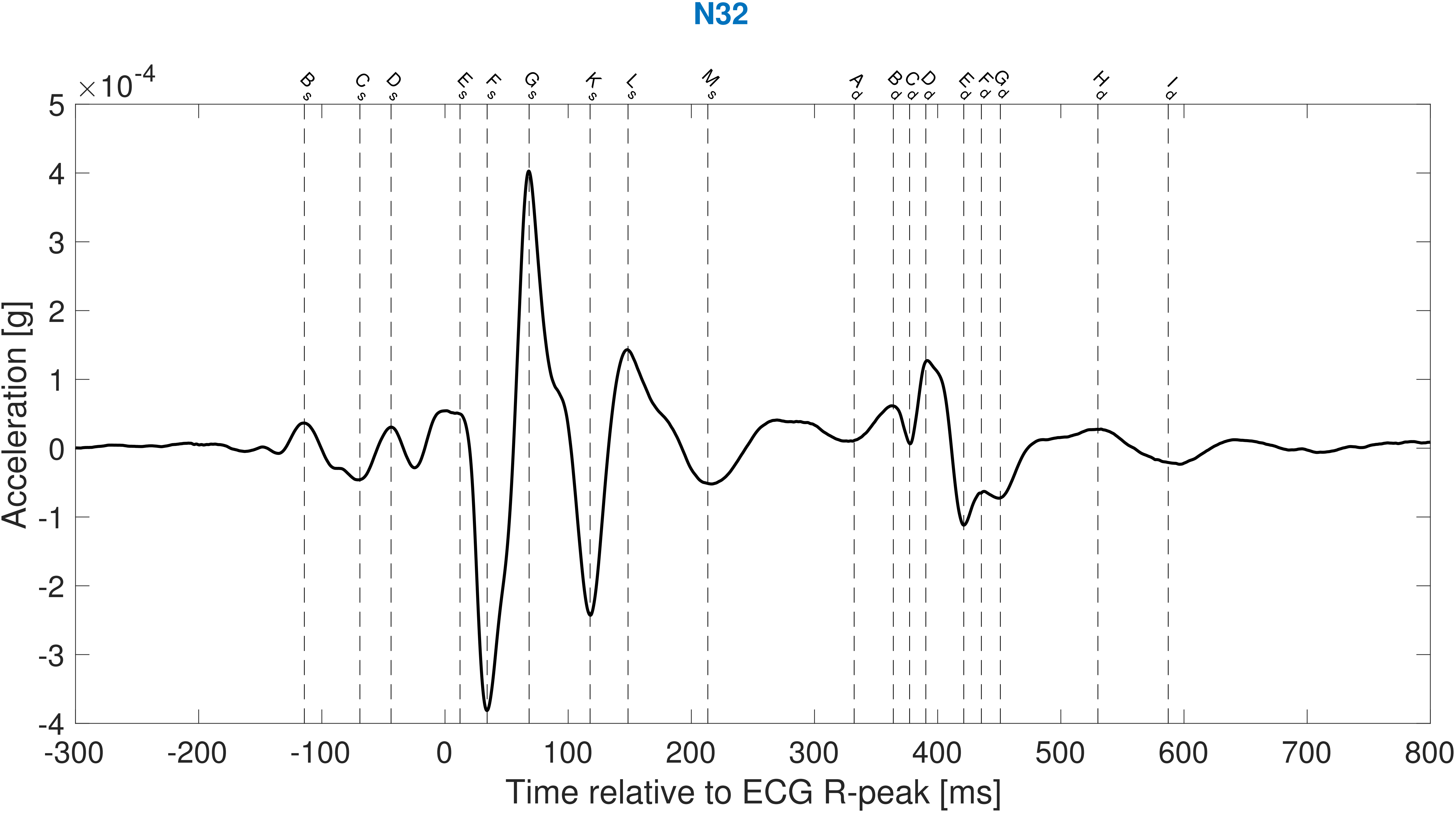

N32
×10^-4
Bs
Cs
Ds
Es
Fs
Gs
Ks
Ls
Ms
Ad
Bd
Cd
Dd
Ed
Fd
Gd
Hd
Id
5
4
3
2
1
0
-1
-2
-3
-4
Acceleration [g]
-300
-200
-100
0
100
200
300
400
500
600
700
800
Time relative to ECG R-peak [ms]

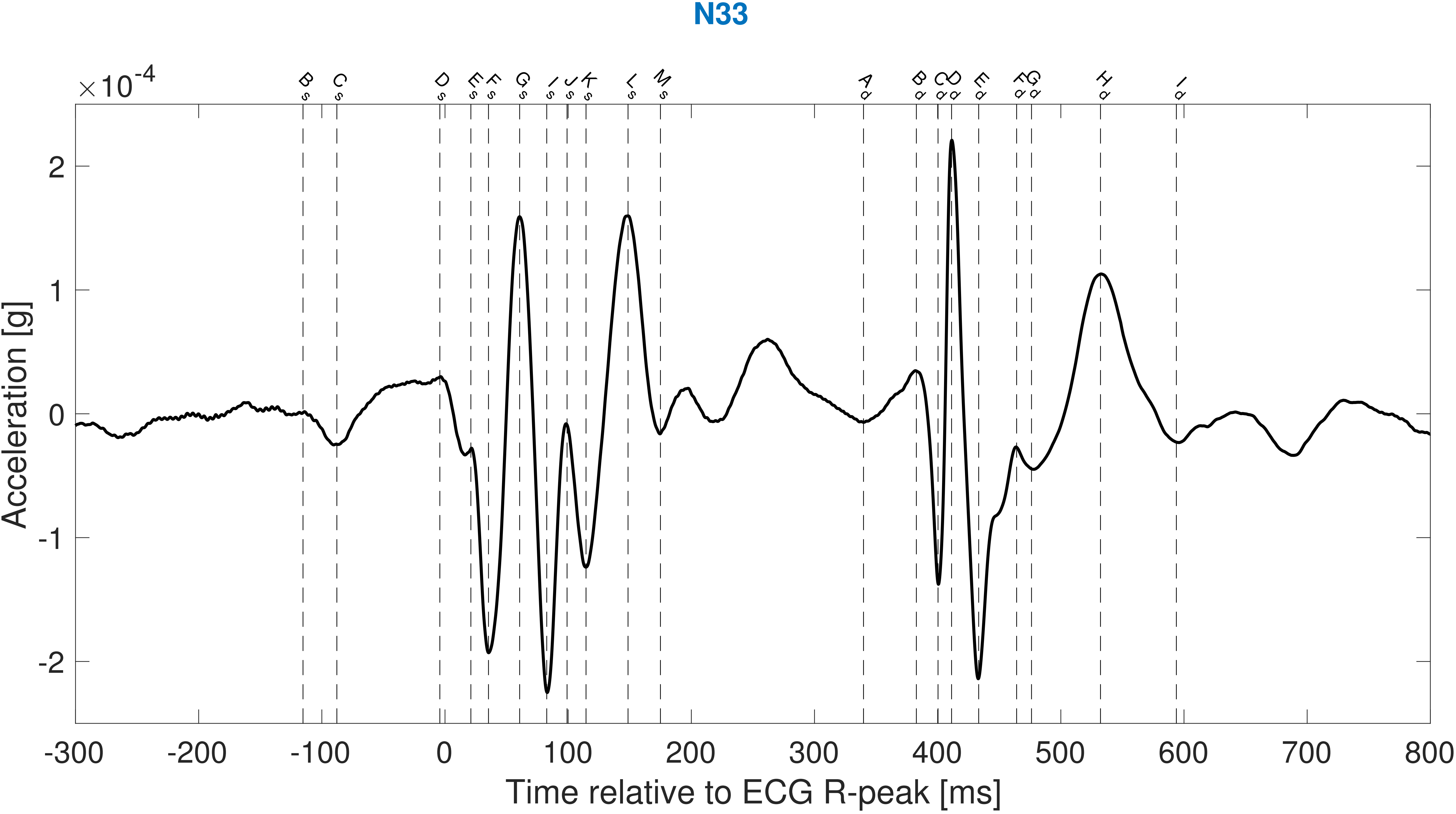

N33
×10^-4
Acceleration [g]
Time relative to ECG R-peak [ms]
B s
C s
D s
E s
F s
G s
I s
J s
K s
L s
M s
A d
B d
C d
D d
E d
F d
G d
H d
I d
2
1
0
-1
-2
-300
-200
-100
0
100
200
300
400
500
600
700
800

N34

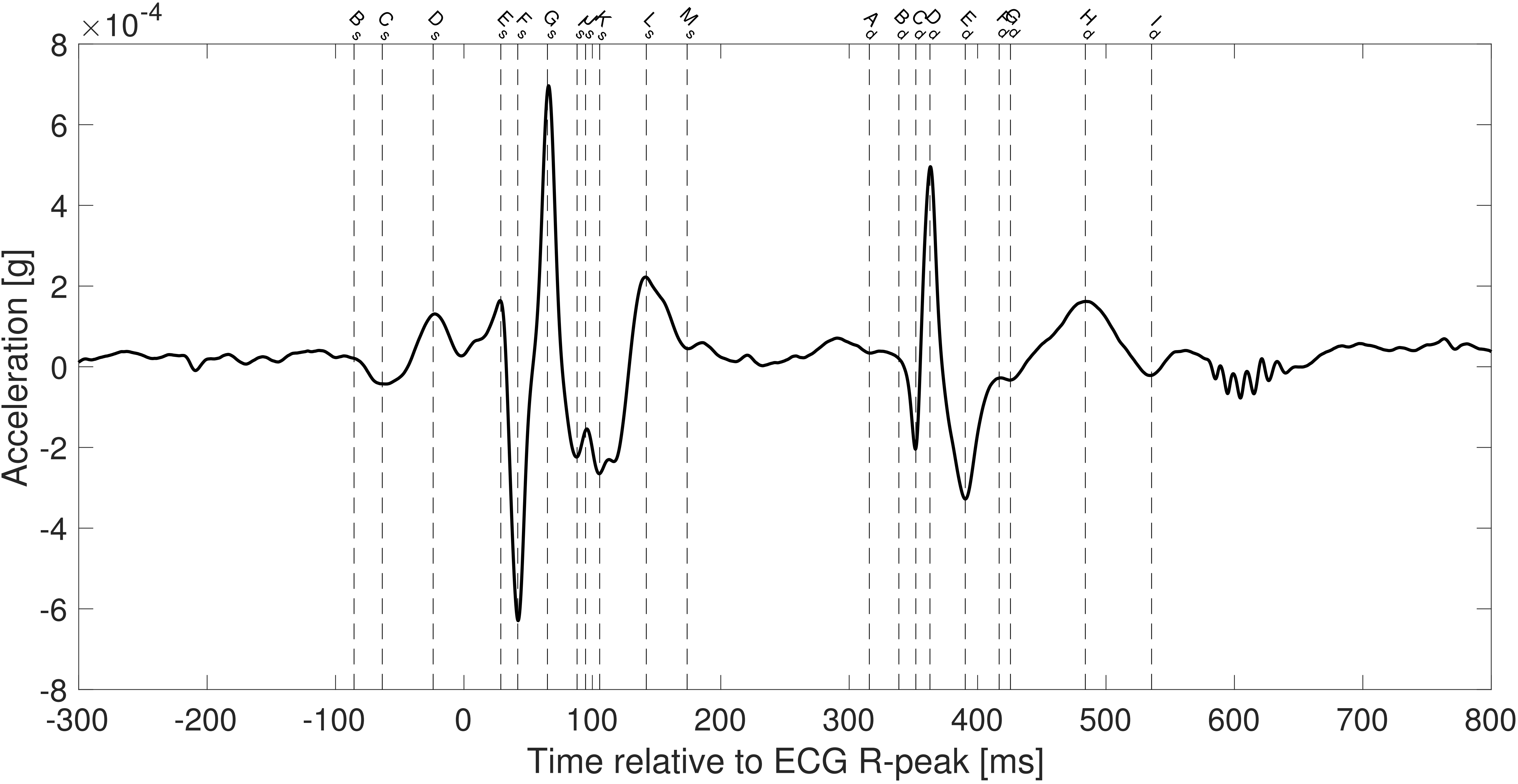

×10^-4
8
6
4
2
0
-2
-4
-6
-8
Acceleration [g]
-300
-200
-100
0
100
200
300
400
500
600
700
800
Time relative to ECG R-peak [ms]

N35

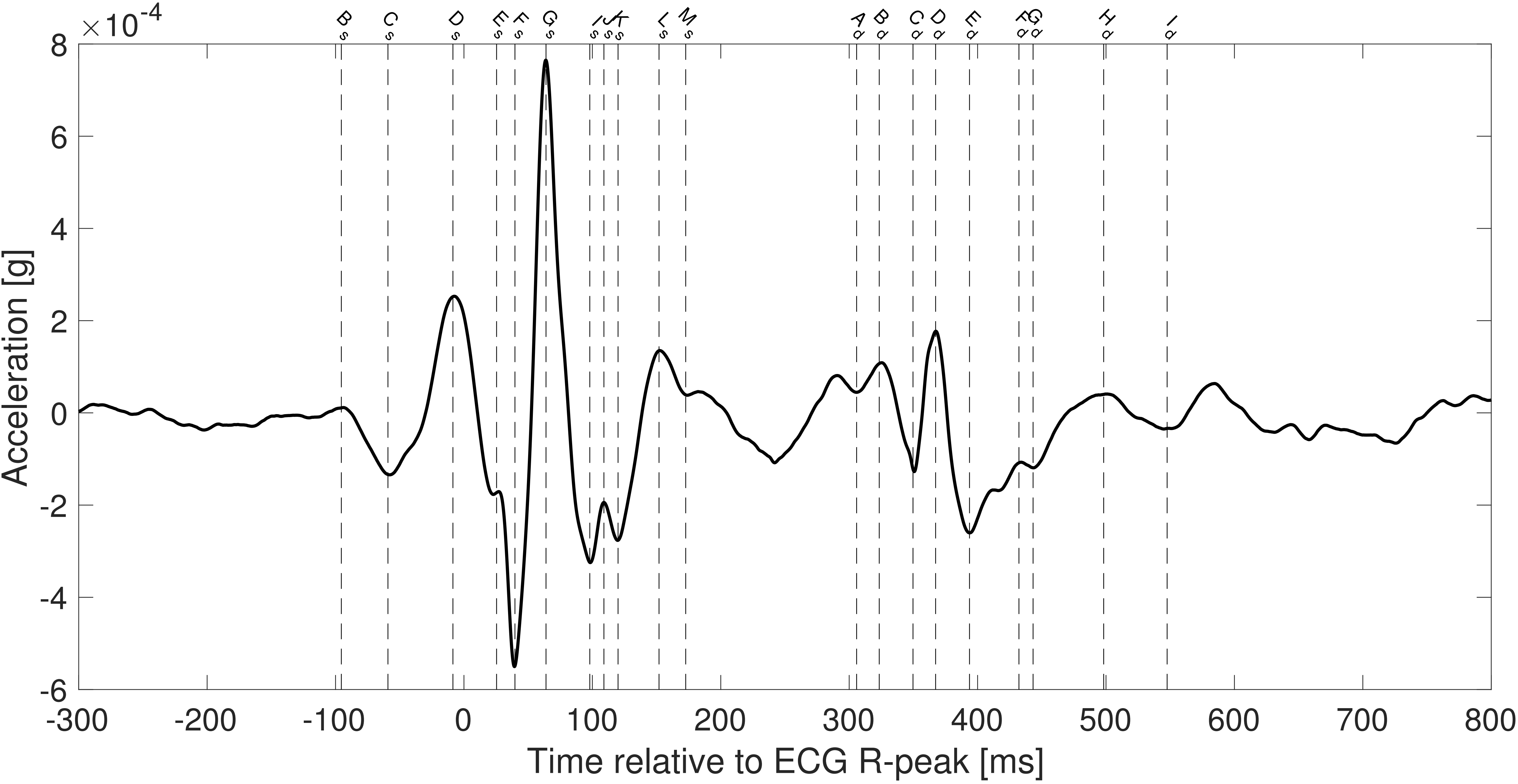

$\times 10^{-4}$
8
6
4
2
0
-2
-4
-6
Acceleration [g]
-300
-200
-100
0
100
200
300
400
500
600
700
800
Time relative to ECG R-peak [ms]
B s
C s
D s
E s
F s
G s
I s
J s
K s
L s
M s
A d
B d
C d
D d
E d
F d
G d
H d
I d

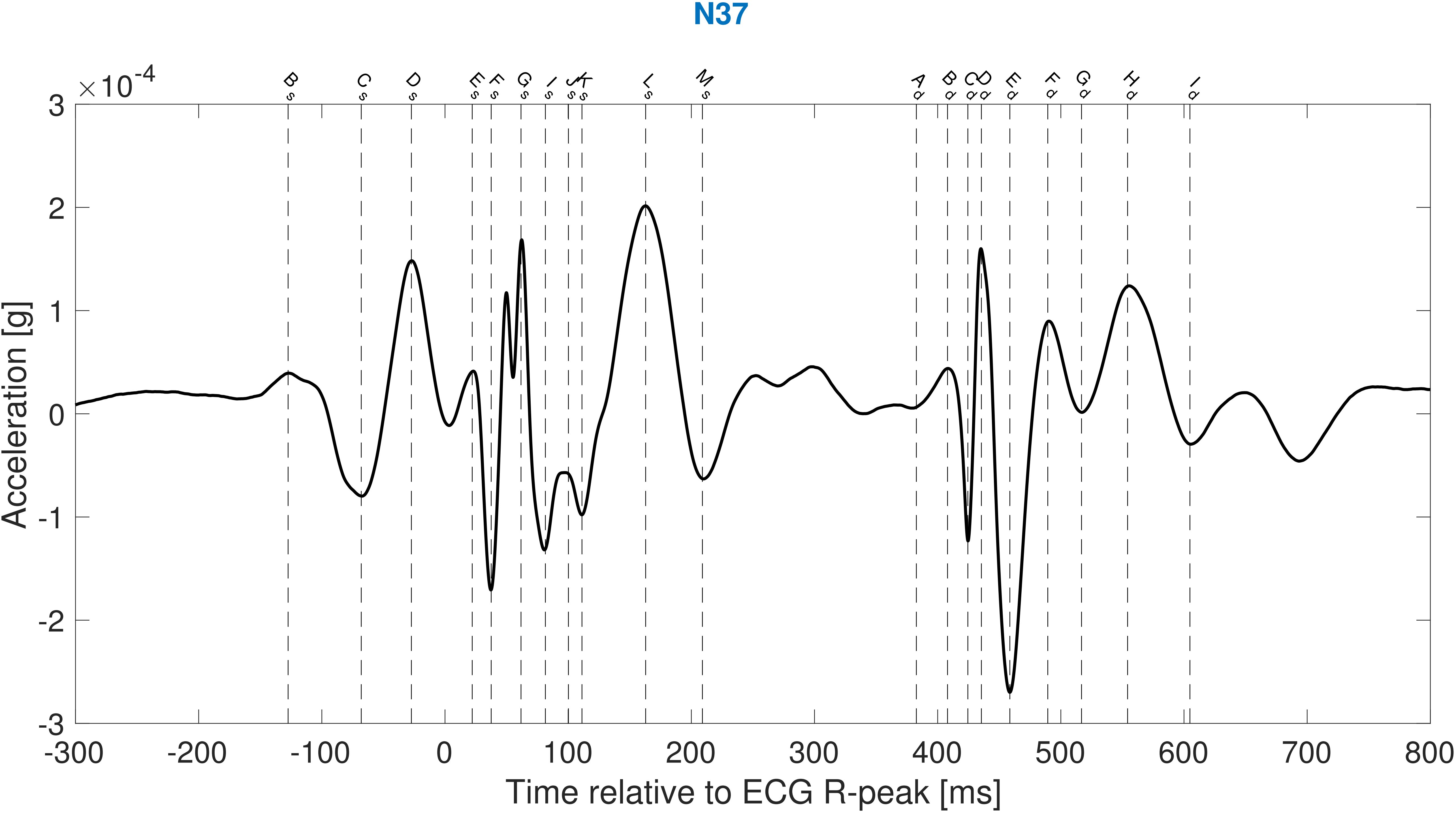

N37
×10^-4
Acceleration [g]
Time relative to ECG R-peak [ms]
-300
-200
-100
0
100
200
300
400
500
600
700
800
3
2
1
0
-1
-2
-3

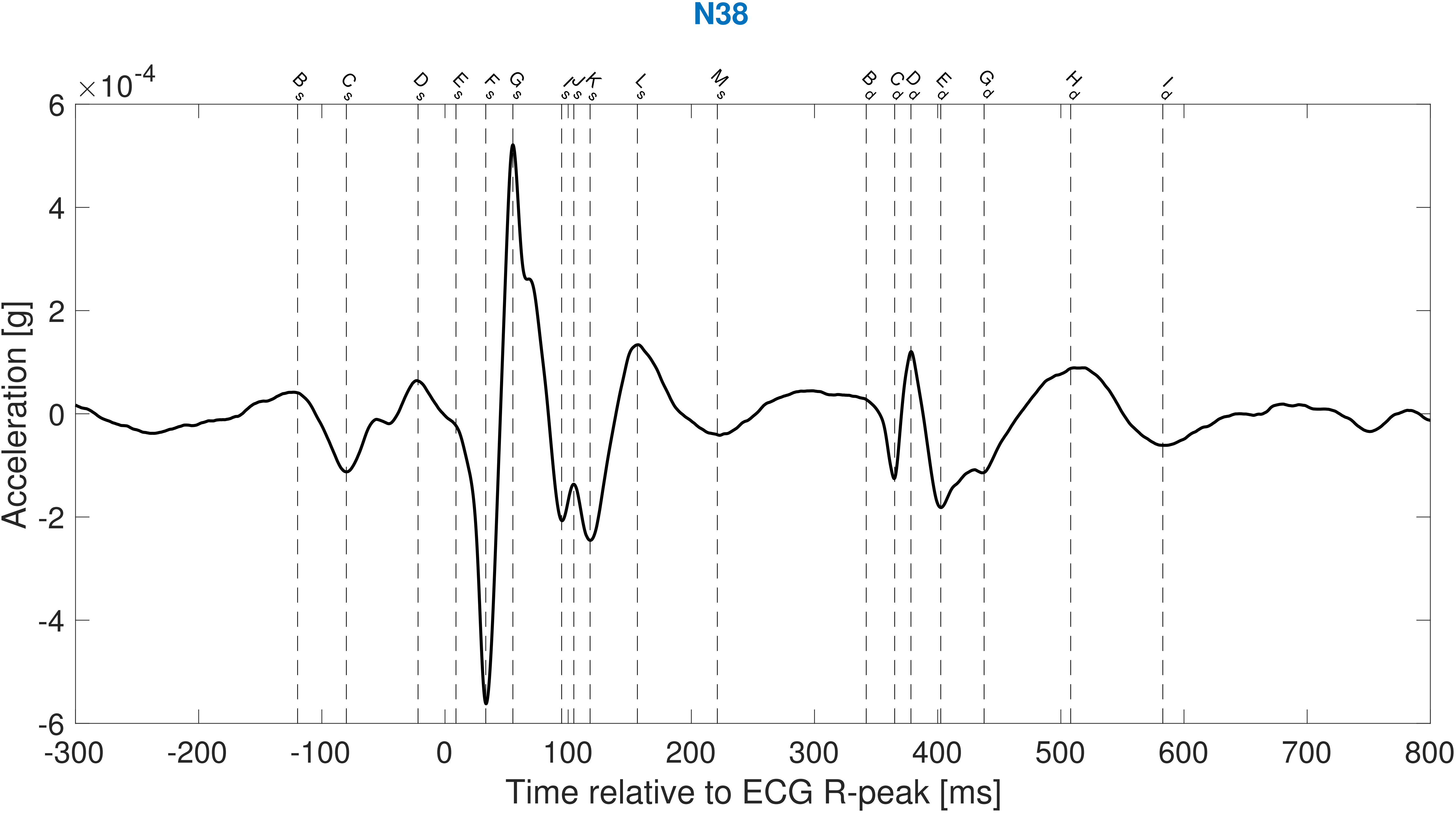
N38
×10^-4
Bs
Cs
Ds
Es
Fs
Gs
Is
Js
Ks
Ls
Ms
Bd
Cd
Dd
Ed
Gd
Hd
Id
6
4
2
0
-2
-4
-6
Acceleration [g]
-300
-200
-100
0
100
200
300
400
500
600
700
800
Time relative to ECG R-peak [ms]

N39

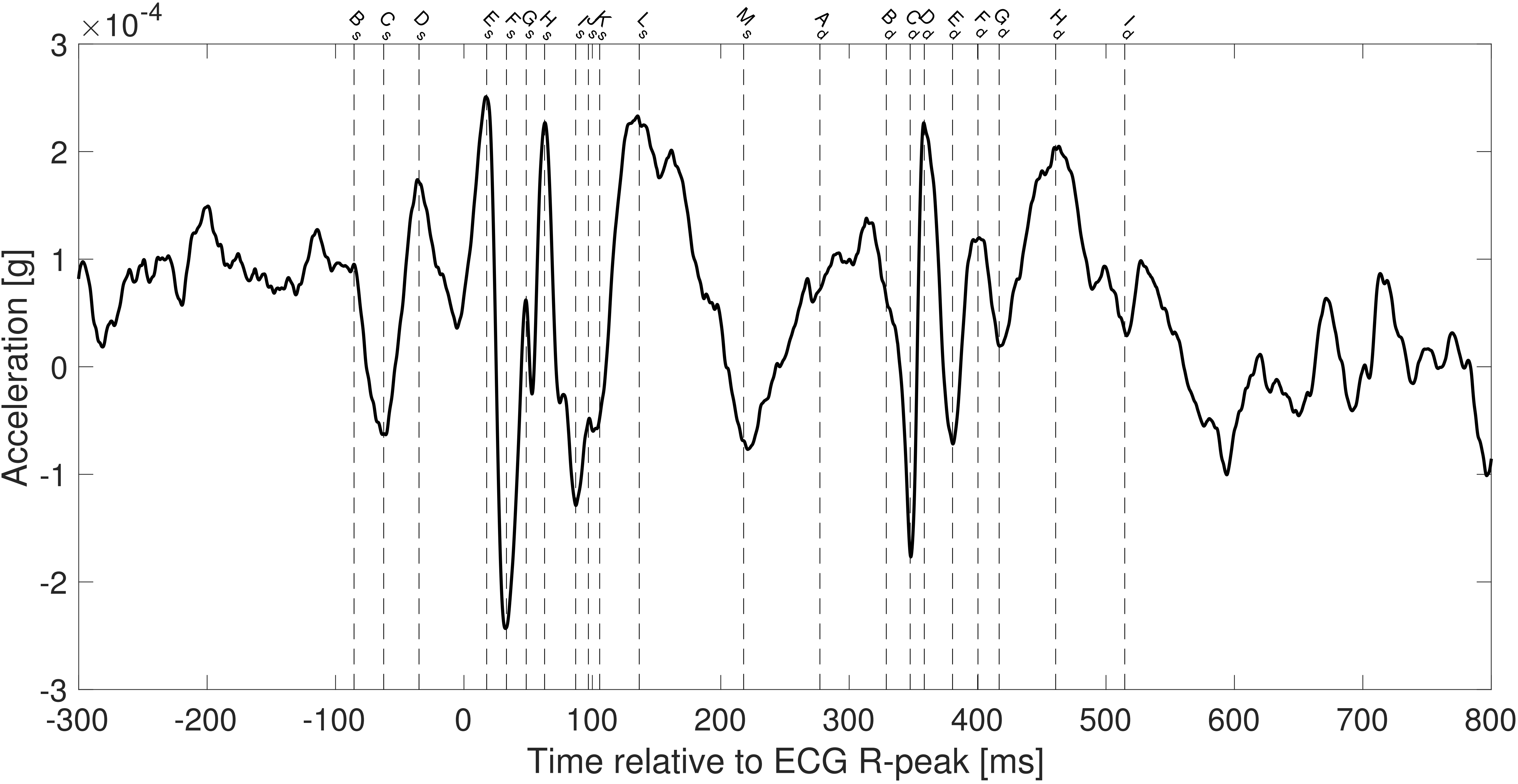

B s
C s
D s
E s
F s
G s
H s
I s
J s
K s
L s
M s
A d
B d
C d
D d
E d
F d
G d
H d
I d
×10^-4
3
2
1
0
-1
-2
-3
Acceleration [g]
-300
-200
-100
0
100
200
300
400
500
600
700
800
Time relative to ECG R-peak [ms]

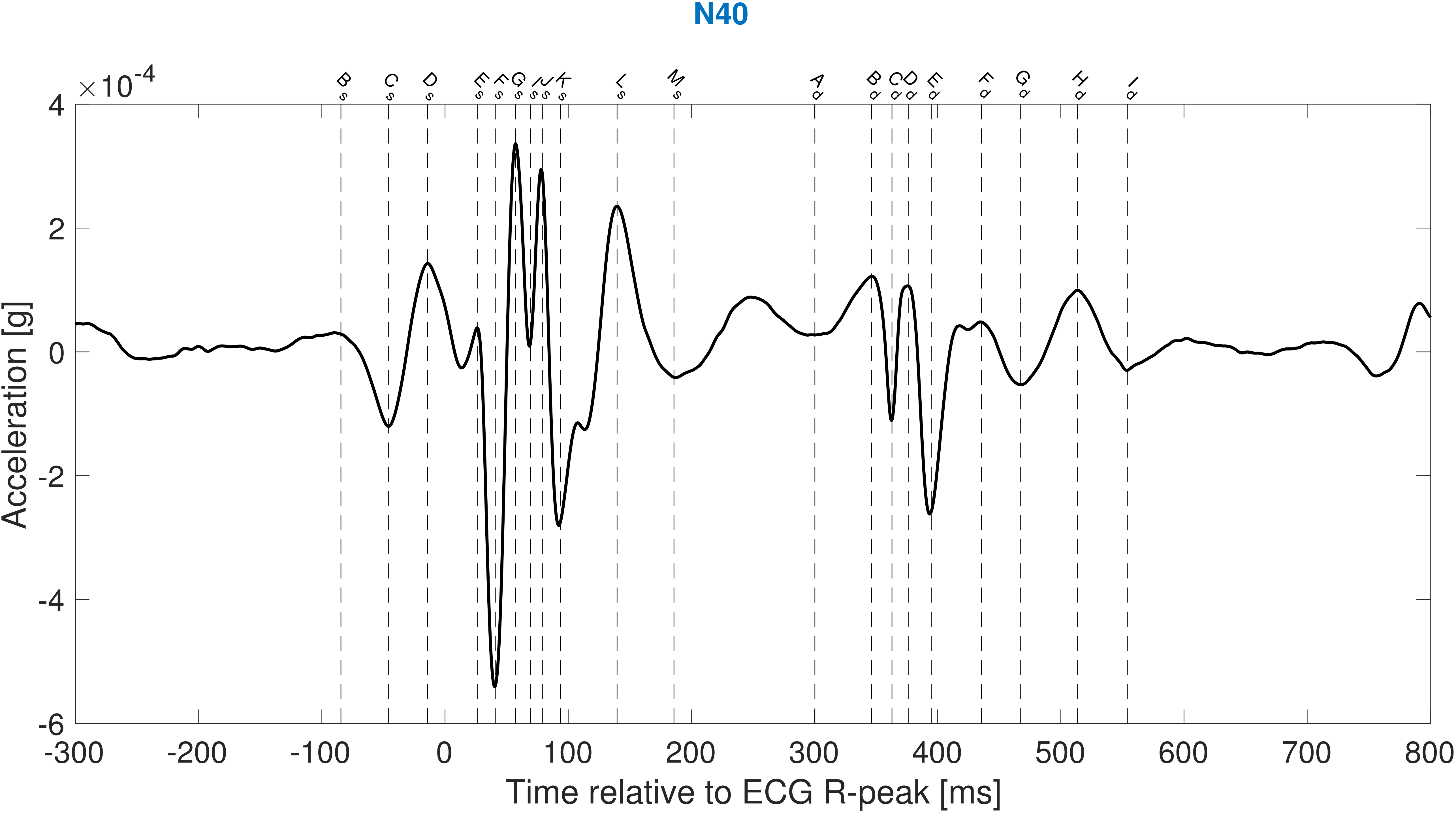

N40
×10^-4
Acceleration [g]
Time relative to ECG R-peak [ms]
4
2
0
-2
-4
-6
-300
-200
-100
0
100
200
300
400
500
600
700
800
B s
C s
D s
E s
F s
G s
I s
J s
K s
L s
M s
A d
B d
C d
D d
E d
F d
G d
H d
I d

N41

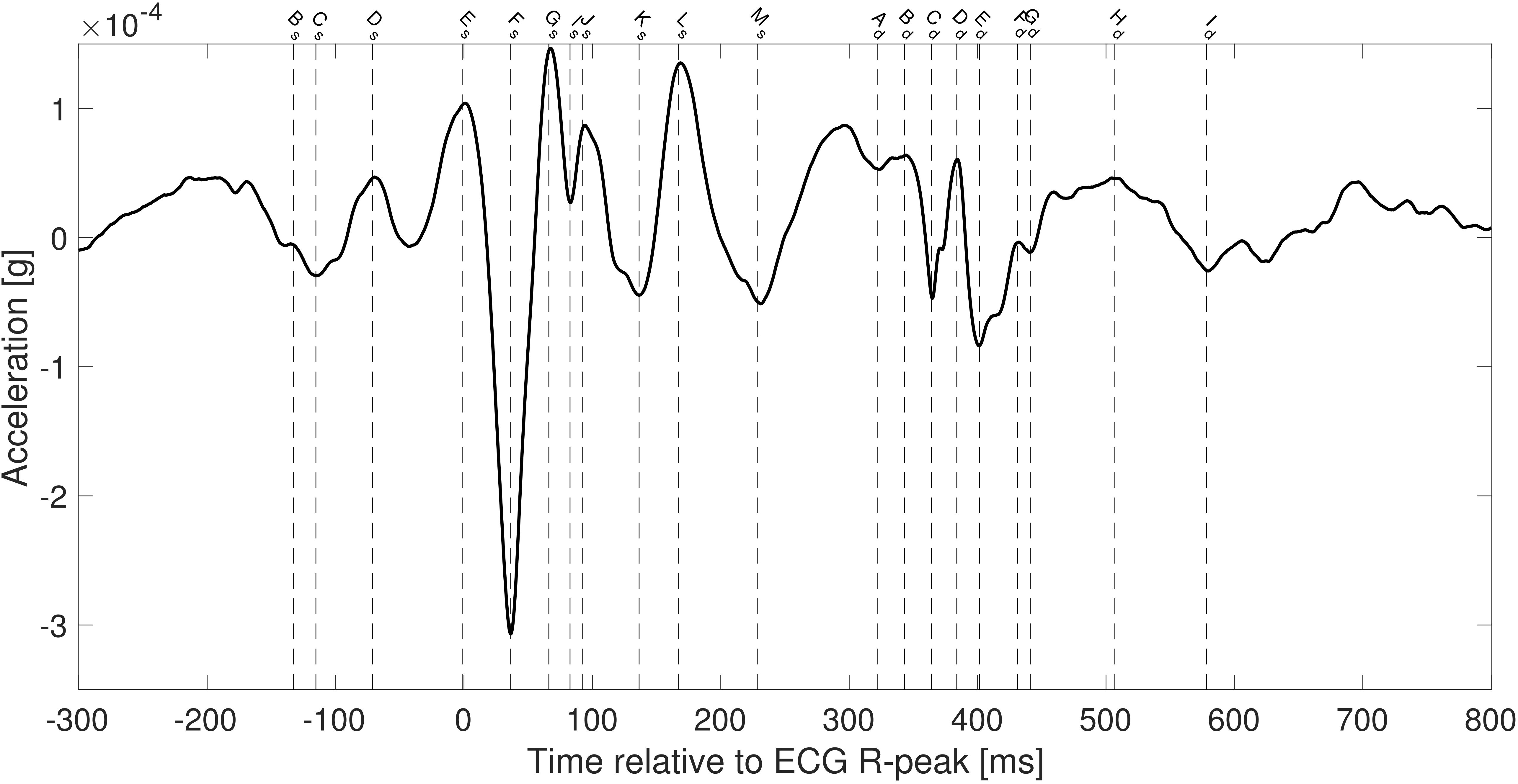

×10^-4
B s
C s
D s
E s
F s
G s
I s
J s
K s
L s
M s
A d
B d
C d
D d
E d
F d
G d
H d
I d
1
0
-1
-2
-3
Acceleration [g]
-300
-200
-100
0
100
200
300
400
500
600
700
800
Time relative to ECG R-peak [ms]

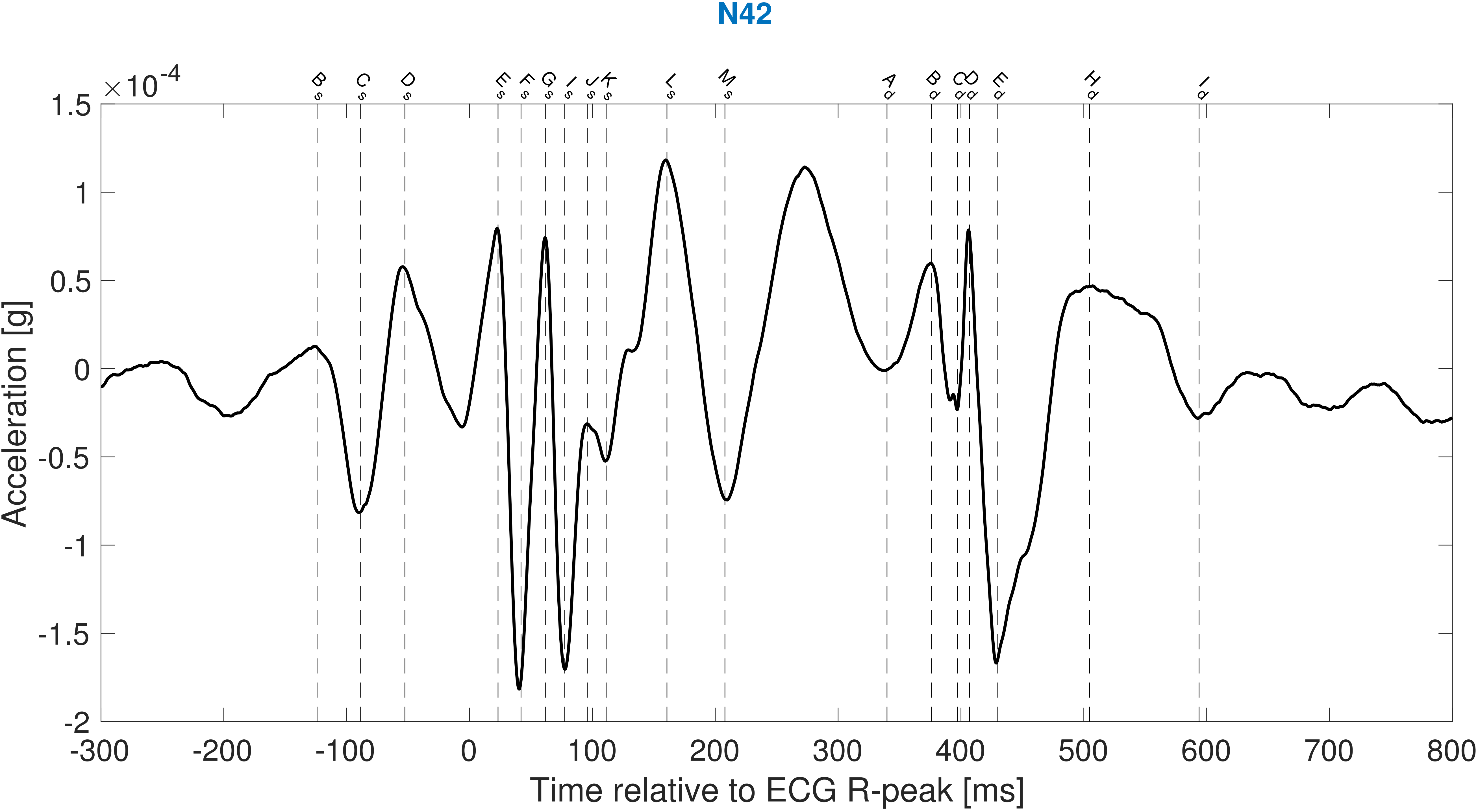
N42
Bs
Cs
Ds
Es
Fs
Gs
Is
Js
Ks
Ls
Ms
Ad
Bd
Cd
Dd
Ed
Hd
Id
×10^-4
Acceleration [g]
Time relative to ECG R-peak [ms]
1.5
1
0.5
0
-0.5
-1
-1.5
-2
-300
-200
-100
0
100
200
300
400
500
600
700
800

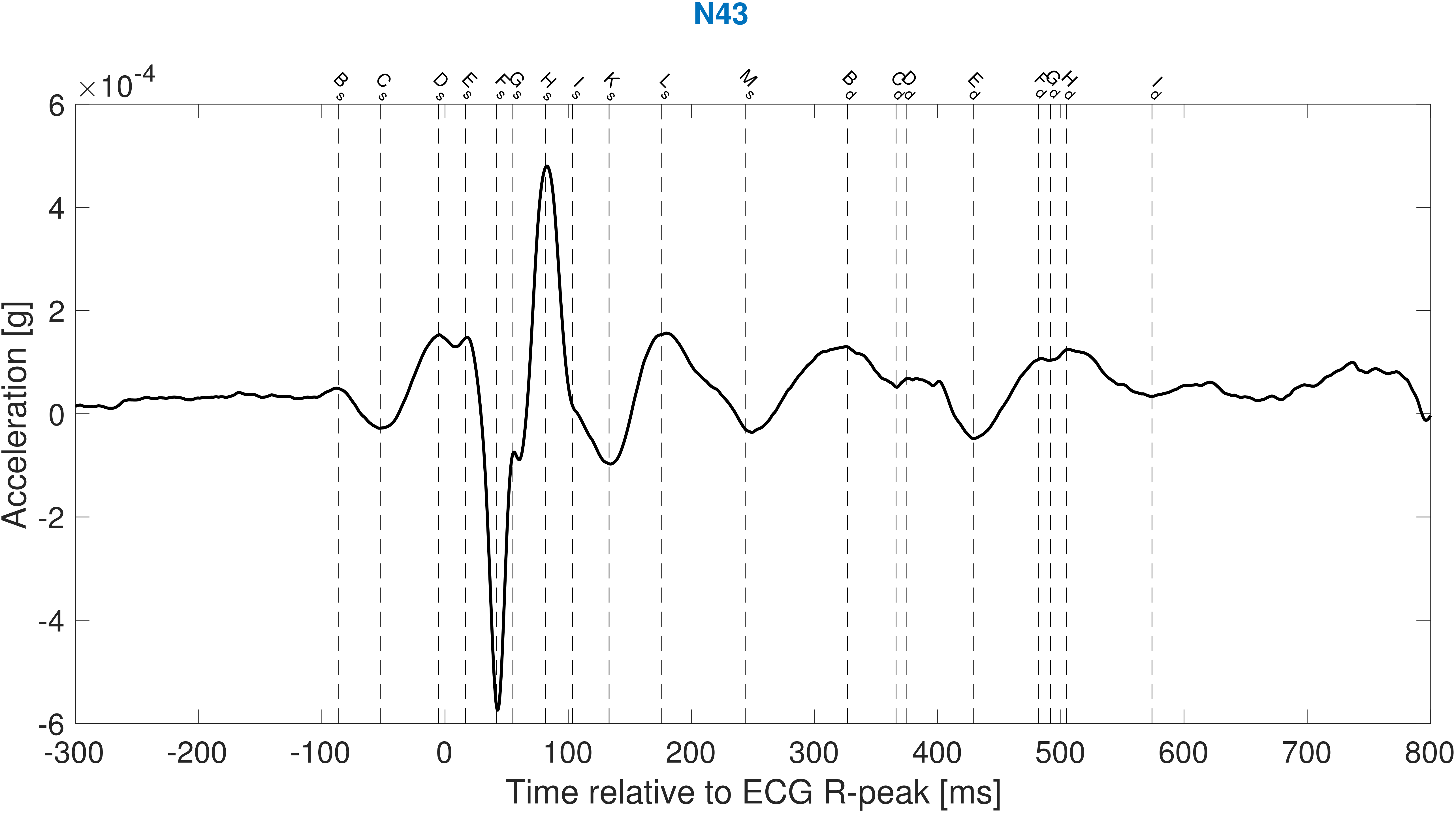

N43
×10^-4
Acceleration [g]
Time relative to ECG R-peak [ms]
6
4
2
0
-2
-4
-6
-300
-200
-100
0
100
200
300
400
500
600
700
800

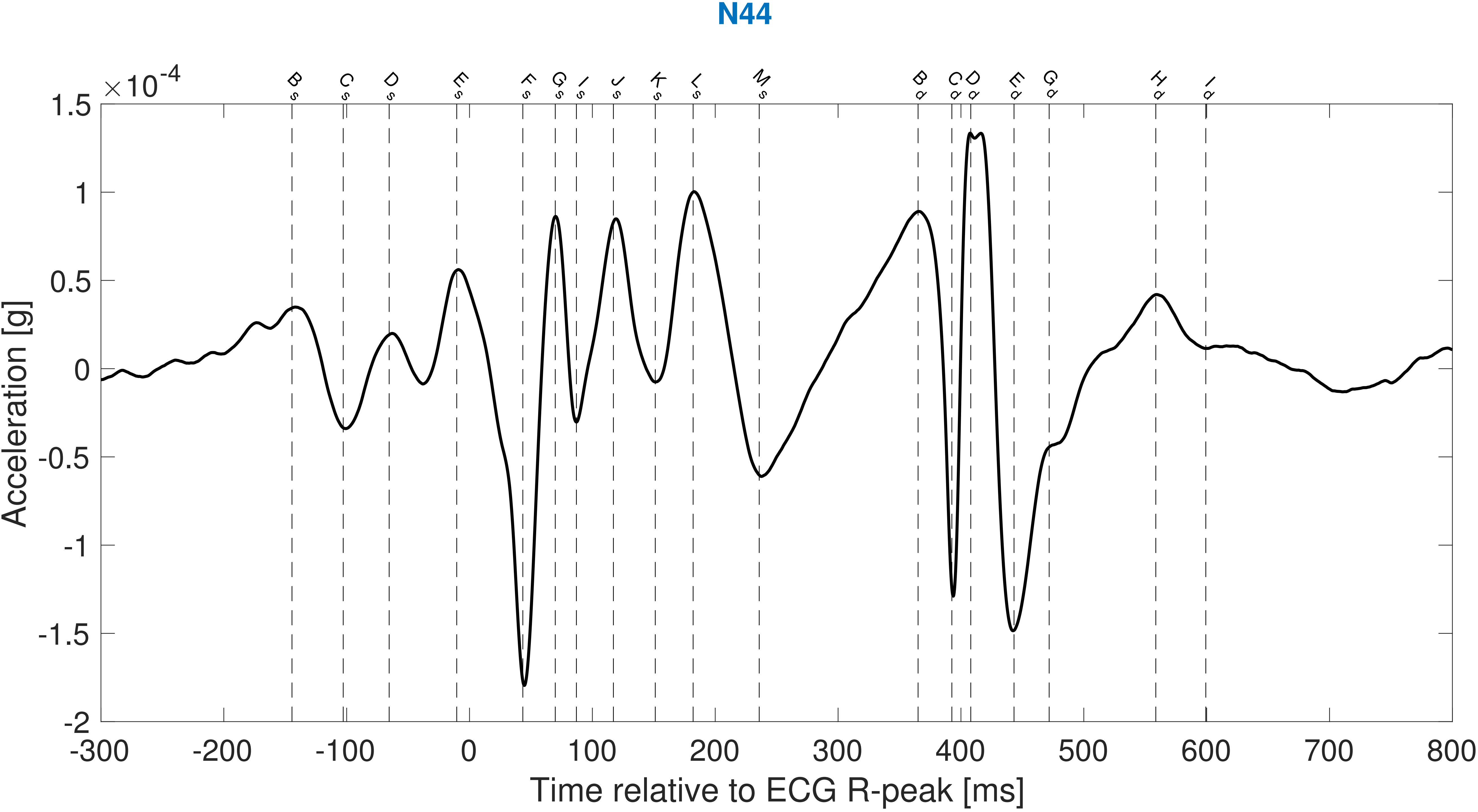
N44
×10^-4
Acceleration [g]
Time relative to ECG R-peak [ms]
1.5
1
0.5
0
-0.5
-1
-1.5
-2
-300
-200
-100
0
100
200
300
400
500
600
700
800
B s
C s
D s
E s
F s
G s
I s
J s
K s
L s
M s
B d
C d
D d
E d
G d
H d
I d
